# Supplementary material for: Protocol for a multicentre cross-sectional, longitudinal ambulatory clinical trial in rheumatoid arthritis and Parkinson’s disease patients analysing the relation between the gut microbiome, fasting and immune status in Germany (ExpoBiome)
Source: BMJ Open. 2023 Aug 18;13(8):e071380. doi: 10.1136/bmjopen-2022-071380 (PMC10441058; doi:10.1136/bmjopen-2022-071380)
Supplement: Author's manuscript [file bmjopen-2022-071380.draft_revisions.pdf]

BMJ Open is committed to open peer review. As part of this commitment we make the peer review history of every article we publish publicly available.

When an article is published we post the peer reviewers' comments and the authors' responses online. We also post the versions of the paper that were used during peer review. These are the versions that the peer review comments apply to.

The versions of the paper that follow are the versions that were submitted during the peer review process. They are not the versions of record or the final published versions. They should not be cited or distributed as the published version of this manuscript.

BMJ Open is an open access journal and the full, final, typeset and author-corrected version of record of the manuscript is available on our site with no access controls, subscription charges or pay-per-view fees (http://bmjopen.bmj.com).

If you have any questions on BMJ Open's open peer review process please email info.bmjopen@bmj.com

BMJ Open

Protocol for a multicentre cross-sectional, longitudinal study in rheumatoid arthritis and Parkinson's disease patients analysing the relation between the gut microbiome, fasting and immune status (ExpoBiome)

Journal:	*BMJ Open*
Manuscript ID	bmjopen-2022-071380
Article Type:	Protocol
Date Submitted by the Author:	23-Dec-2022
Complete List of Authors:	Hansen, Bérénice; LCSB<br>Laczny, Cédric C.; LCSB<br>Aho, Velma T.E.; LCSB<br>Frachet-Bour, Audrey; LCSB<br>Habier, Janine; LCSB<br>Ostaszewski, Marek; LCSB<br>Michalsen, Andreas; Charité Universitätsmedizin Berlin, Institute for Social Medicine, Epidemiology and Health Economics; Immanuel Hospital Berlin-Wannsee Branch, Department of Internal and Integrative Medicine<br>Hanslian, Etienne; Charite Universitatsmedizin Berlin, Institute for Social Medicine, Epidemiology and Health Economics; Immanuel Hospital Berlin-Wannsee Branch<br>Koppold-Liebscher, Daniela; Charité Universitätsmedizin Berlin, Institute for Social Medicine, Epidemiology and Health Economics; Immanuel Hospital Berlin-Wannsee Branch<br>Hartmann, Anika; Charité Universitätsmedizin Berlin, Institute of Social Medicine, Epidemiology and Health Economics; Charité Universitätsmedizin Berlin, Department of Dermatology, Venereology and Allergology<br>Steckhan, Nico; Charité Universitätsmedizin Berlin, Institute for Social Medicine, Epidemiology and Health Economics; University of Potsdam, Digital Health - Connected Healthcare, Hasso Plattner Institute<br>Mollenhauer, Brit; University Medical Center Göttingen; Paracelsus-Kliniken Deutschland GmbH<br>Schade, Sebastian; University Medical Center Göttingen; Paracelsus-Kliniken Deutschland GmbH,<br>Roomp, Kirsten; LCSB<br>Schneider, Jochen; LCSB; Saarland University Hospital and Saarland University Faculty of Medicine, Department of Internal Medicine and Psychiatry<br>Wilmes, Paul; LCSB; University of Luxembourg, Department of Life Sciences and Medicine
Keywords:	IMMUNOLOGY, Rheumatology < INTERNAL MEDICINE, MICROBIOLOGY, Parkinson-s disease < NEUROLOGY, NUTRITION & DIETETICS, Clinical trials < THERAPEUTICS

SCHOLARONE™
Manuscripts

Protocol for a multicentre cross-sectional, longitudinal study in rheumatoid arthritis and Parkinson's disease patients analysing the relation between the gut microbiome, fasting and immune status (ExpoBiome)

Bérénice Hansen[1], Cédric C. Laczny[1], Velma T.E. Aho[1], Audrey Frachet-Bour[1], Janine Habier[1], Marek Ostaszewski[1], Andreas Michalsen[4,5], Etienne Hanslian[4,5], Daniela A. Koppold[4,5,8], Anika Hartmann[4,10], Nico Steckhan[4,9], Brit Mollenhauer[6,7], Sebastian Schade[6,7], Kirsten Roomp[1], Jochen G. Schneider[1,3+], Paul Wilmes[1,2+]

[1]Luxembourg Centre for Systems Biomedicine, University of Luxembourg, Campus Belval, 7, avenue des Hauts-Fourneaux, L-4362 Esch-sur-Alzette, Luxembourg

[2]Department of Life Sciences and Medicine, University of Luxembourg, 7, avenue des Hauts-Fourneaux, L-4362 Esch-sur-Alzette, Luxembourg

[3]Department of Internal Medicine and Psychiatry, Saarland University Medical Center, D- 66421 Homburg Saar, Germany

[4]Institute for Social Medicine, Epidemiology and Health Economics, Charité – Universitätsmedizin Berlin, corporate member of Freie Universität Berlin, Humboldt-Universität zu Berlin, and Berlin Institute of Health

[5]Department of Internal and Integrative Medicine, Immanuel Hospital Berlin, Germany

[6]Paracelsus-Elena-Klinik, Kassel, Germany

[7]University Medical Center Göttingen, Germany

[8]Department of Pediatrics, Division of Oncology and Hematology, Charité – Universitätsmedizin Berlin, Corporate Member of Freie Universität Berlin, Humboldt-Universität zu Berlin and Berlin Institute of Health, Berlin, Germany

[9]Digital Health - Connected Healthcare, Hasso Plattner Institute, University of Potsdam, Potsdam, Germany

[10]Department of Dermatology, Venereology and Allergology, Charité—Universitätsmedizin Berlin, Corporate Member of Freie Universität Berlin and Humboldt-Universität zu Berlin, Berlin, Germany

[+]contributed equally

*Correspondence to:

Jochen Schneider (jochen.schneider@uni.lu)
Paul Wilmes (paul.wilmes@uni.lu)

Luxembourg Centre for Systems Biomedicine,
University of Luxembourg, Campus Belval,
7, avenue des Hauts-Fourneaux,
L-4362 Esch-sur-Alzette, Luxembourg

Word count: 4143

For peer review only

Abstract

Introduction

Chronic inflammatory diseases such as rheumatoid arthritis (RA) and neurodegenerative disorders like Parkinson's disease (PD) have recently been associated with a decreased diversity in the gut microbiome, emerging as key driver of various diseases. The specific interactions between gut-borne microorganisms and host pathophysiology remain largely unclear so far. The microbiome can be modulated by interventions comprising modulated nutrition and food intake.

The aim of our clinical study is to examine (1) the effects of prolonged fasting and time-restricted eating (TRE) on the outcome parameters and the immunophenotypes of RA and PD with (2) special consideration of microbial taxa and molecules associated with the changes expected in (1) and (3) to identify factors that impact the disease course and treatment by in depth screening of microorganisms and molecules in personalised HuMiX gut-on-chip models, ideally to find novel targets for anti-inflammatory therapy.

Methods and Analysis

This trial is an open-label, multicentre, controlled clinical trial consisting of a cross-sectional and a longitudinal study. A total of 180 patients is recruited. For the cross-sectional study, patients with PD, patients with RA and healthy controls are recruited at two different, specialized clinical sites. For the longitudinal part, patients with PD and RA undergo 5-7 days of prolonged fasting (PF) followed by TRE (16:8) for a period of 12 months. One baseline visit takes place before the PF intervention and 10 follow-up visits will follow over a period of 12 months.

Ethics and dissemination

Ethical approval was obtained to plan and conduct the trial from the institutional review board of the Charité-Universitätsmedizin Berlin (EA1/204/19), the ethics committee of the state medical association (Landesärztekammer) of Hessen (2021-2230-zvBO) and the Ethics Review Panel (ERP) of the University of Luxembourg (ERP 21-001-A ExpoBiome). The results of this study will be disseminated through peer-reviewed publications and scientific presentations.

Trial registration number at clinicaltrials.gov:

NCT04847011

Key words: Microbiome, fasting therapy, intermittent fasting, time restricted eating, chronic disease, rheumatoid arthritis, Parkinson's disease, nutrition, chronic diseases, ExpoBiome, inflammation, gut on a chip, HuMiX, immunophenotype

Strengths and limitations of the study

- The participants of the longitudinal study will be closely monitored for 12 months and routine blood parameters as well as anthropometric data and questionnaires will be precisely documented
- This study will identify novel microbiome-derived common and disease-associated molecules involved in immune system modulation in two major chronic diseases: RA and PD.
- This study aims at also identifying novel targeted pathways to control chronic inflammatory conditions in the future
- A limitation is the heterogeneity of the cohorts regarding age and sex, which is due to the prevalence of the diseases: RA is more common in women, while PD is more common in men and has a later disease onset.
- A bias exists in choosing RA and PD as chronic disorders to study immunophenotypes although generalisable results are targeted

Introduction (1018)

The human microbiome is emerging as a key driver of various diseases through its complex of distinct yet connected biomolecules (referred to as the "*expobiome*")[1, 2]. The expobiome is comprised of a diverse set of nucleic acids, polypeptides and metabolites which, in the gut alone, are present in substantial concentrations[1]. However, the specific interactions between gut-borne microorganisms and host (patho)physiology remain largely unknown. Although host genetics shape the composition of the gut microbiome, the latter is particularly influenced by non-genetic factors such as lifestyle and diet[3, 4]. Therefore, the microbiome is a plausible target to modify health outcomes.

Individuals suffering from chronic diseases, including autoimmune, metabolic, and neurodegenerative diseases as well as cancer, often present alterations from a gut microbiome composition associated with health. These shifts are typically characterised by an overgrowth of one or several microbial species with likely adverse effects including pathobionts, as well as a decrease in beneficial taxa[5]. Such imbalances are referred to as dysbiosis. Although structural microbiome changes are clearly detectable, the mechanistic or functional consequences of dysbiosis are still largely unknown. They may, however, result in dysregulated interactions with the immune system[6]. Considering the intricacy of the immune system, the question arises whether the observed microbiome changes are cause or consequence of disease. This implies that, in addition to the genetic predisposition of the host, the gut microbiome needs to be considered as a potential pathogenic factor or major driver in disease onset and course[3, 4].

RA and PD are two specific examples representing dysregulated microbiome-immune system interactions [7, 8]. RA is a multifactorial, chronic and systemic autoimmune disease, primarily affecting the lining of the synovial joints with a higher risk and younger age for disease onset in women and a global prevalence of 1%[9, 10]. The exact disease pathogenesis is still unclear and no cure for RA currently exists. In addition to the common local articular symptoms of

For peer review only

RA, systemic comorbidities can affect the vasculature, metabolism and bones[11]. Besides various environmental risk factors e.g. smoking and a Western diet, the host microbiome is associated with the pathophysiology of the disease[12]. The diversity of the gut microbiome has been reported to be decreased in individuals with RA, compared with the general population, and is correlated with disease duration and autoantibody levels[13]. Studies in murine models also report that autoimmune arthritis is strongly attenuated under germ-free conditions[14]. The introduction of specific bacteria, e.g. segmented filamentous bacteria, into germ-free animals or oral infection with *Porphyromonas gingivalis* drive autoimmune arthritis through activation of T helper cells[14]. Several different taxa, including *Prevotella copri*, *Lactobacillus* spp. and *Colinsella* spp. are enriched in the gut microbiome of patients with RA and correlate positively with disease markers e.g. immunoglobulins IgA and IgG , while other taxa like *Haemophilus* spp. and *Faecalibacterium* spp. are typically found at lower abundances in patients with RA compared to healthy individuals[13, 15, 16]. Alterations of the gut microbiome may therefore have an important impact on RA pathophysiology[12].

PD affects 0.4-2% of the population above 65 years worldwide and is the second most common progressive neurodegenerative disease with men being 1.5 times more likely to be affected than women[17]. The cardinal symptoms are motor deficiencies such as tremor and rigidity, but also include a wide range of non-motor symptoms, such as hyposmia, depression, insomnia or cognitive impairment, severely impacting patients' quality of life[18]. Aggregations of the protein α-synuclein in the dopaminergic substantia nigra represent the main neuropathological manifestations[19]. Recent literature suggests that neuroinflammation plays a key role in early phases of PD-like neurodegeneration, with microglia as the main cellular effectors[20]. Dysbiosis of the gut microbiome has been associated with the characteristic motor deficits and pathophysiologic changes in the enteric and central nervous systems in animal studies. Increased relative abundances of the genera *Akkermansia, Bifidobacterium, Lactobacillus,* and *Methanobrevibacter* and decreased abundances in *Faecalibacterium* and *Roseburia* have been reported[21, 22]. The PD-associated loss of dopaminergic neurons involves mechanisms of inflammatory and autoimmune responses. The majority of patients with PD suffer from gastrointestinal symptoms, e.g. constipation and irritable bowel syndrome (IBS) -like symptoms[23]. Recently it has been suggested that the gut-brain axis, e.g. by-products produced by the gut microbiome, may contribute to the production of α-synuclein aggregates in the enteric nervous system[24]. In addition, increased intestinal permeability[25] and enteric inflammation occur in PD and substantiate a role of peripheral inflammation in the initiation and the progression of the disease[26].

One factor with known major impact on the gut microbiome and on chronic diseases is diet[7]. Dietary approaches, including fasting, have been used as therapeutic interventions at least since the 5th century BCE[27, 28]. However, the available evidence from robust comparable clinical studies is lacking. While PF of 7–10 days has been associated with improvements in RA[29, 30], the mechanism behind the decrease in inflammatory processes during caloric deprivation remains unclear. The improvements can typically only be maintained for a limited period of time, and the symptoms can reappear after reintroduction of the patients' standard diet. In mouse models of PD, intermittent fasting (IF) has led to several improvements including decreased excitotoxicity, reduced neurodegeneration and protection against autonomic dysfunction, motor and cognitive decline[31].

For peer review only

IF and PF may have potent immunomodulatory effects which are partially mediated by the gut microbiome and the fasting induced alterations of the latter[32]. These microbial shifts include upregulation of *Akkermansia muciniphila*, *Bacteroides fragilis*, other *Bacteroides* spp., Proteobacteria, and butyric acid producing *Lachnospiraceae,* but also *Odoribacter*, which is negatively associated with blood pressure[33, 34]. Interestingly, an overall decrease of the Firmicutes/Bacteroidetes ratio could be observed, a high ratio is commonly associated with several pathologies, including RA [35].

To our knowledge no clinical trials have been investigating the connection between IF or PF and PD in humans so far[31]. Our study aims to elucidate the causal relationship between the gut microbiome and the immune system. To do so, we will use analyses of the molecular basis of human-microbiome interactions enabled by high throughput methodologies such as the combination of metagenomics, metatranscriptomics and metaproteomics. Moreover, we are aiming at identifying new genes, proteins, metabolites and host pathways facilitating the development of novel diagnostic and therapeutic tools[36, 37].

Methods and Analysis (2256)

Study objectives

The first objective of the study is to define specific gut microbiome-derived molecules in RA and PD, compared to healthy individuals, and relate this information to the immunophenotypes of the individuals. The second objective is to identify and track common and disease-specific molecular signatures to predict the outcome of a gut microbiome-targeted therapeutic intervention, here fasting, on inflammation-driven symptoms in RA and PD. The third objective of the study is to identify and validate microbiome-derived effector molecules which downregulate pro-inflammatory innate and adaptive immune pathways.

Study design

The ExpoBiome cohort consists of 180 adult individuals, meeting the exclusion and inclusion criteria (Table 1), for the cross-sectional study (objectives 1 and 3) and 60 adult individuals for the longitudinal study (objectives 2 and 3). There are five different arms in total: (1) RA – cross-sectional arm [60 patients], (2) PD – cross-sectional arm [60 patients], and (3) healthy controls – cross-sectional arm [60 patients], (4) RA – longitudinal arm [30 patients], (5) PD – longitudinal arm [30 patients] (**Error! Reference source not found.**).

At the first visit (T0), patients answer several questionnaires, and blood, urine, saliva and stool samples are obtained (Table 2).
The longitudinal arms (1) and (2) undergo a 5–7-day PF with a dietary energy supply of max. 350-400 kcal per day with vegetable or grain broths as well as fresh vegetable juices[28, 33]. After the PF, the longitudinal arms follow a dietary regimen including the concept of TRE for a period of 12 months following the 16:8 pattern[38]. This means that food intake is allowed ad libitum for 8 h, followed by 16 h of fasting where no food should be consumed. The intake of non-caloric beverages, e.g. water, unsweetened tea or coffee is, however, allowed. The

For peer review only

participants attend one follow-up visit (T2) during the PF and 9 follow-up visits during the 12 months of TRE (**Error! Reference source not found.**).

Patient and Public Involvement

Feedback of patients during former clinical trials at the study centre in Berlin was integrated in the planning and design of the fasting intervention of this study. Patients are not involved in the conduct, reporting, or dissemination plans of this research.

Recruitment and randomisation

Patients are recruited by the specialised sites via different sources, e.g. by direct referral from either a physician at the Immanuel Hospital Berlin and the outpatient department of the Institute of Social Medicine, Epidemiology and Health Economics at Charité-Universitätsmedizin Berlin, or the Paracelsus-Elena Clinic in Kassel, or by non-personal advertising strategies (e.g. flyers or social media).

Participants meeting all the inclusion and no exclusion criteria (Table 1) are assigned to their respective groups (RA, PD or healthy control) (**Error! Reference source not found.**) for the cross-sectional study after written informed consent. Half of the patients from the RA group and half of the patients from the PD group is selected to take part in the longitudinal part of the study, including the fasting intervention according to their availability for all 11 visits and their willingness to follow TRE over 12 months. This study is an open-label trial, as blinding is not feasible in fasting interventions.

Table 1: Inclusion and exclusion criteria

Inclusion criteria	Exclusion criteria
• age 18-79<br>• one of the following diagnoses: rheumatoid arthritis (first diagnosis >6 weeks ago), Parkinson's disease OR healthy volunteer<br>• control ("healthy") individuals must be without any evidence of active known or treated RA, without any evidence of active, known or treated central nervous system disease, and without a known family history of idiopathic PD<br>• control individuals should match the RA or PD individuals as closely as possible (sex, age, education)<br>• present written declaration of consent<br>• ability to understand the patient information and willingness to sign the consent form	• gout or proven bacterial arthritis<br>• participation in another study<br>• existing/current eating disorder (bulimia nervosa, anorexia nervosa) within the past 5 years<br>• severe internal disease (e.g. kidney deficiency with creatinine > 2mg/dl)<br>• existing vegan diet or fasting during the last six months<br>• presence or suspicion of atypical PD (e.g. early dementia, early autonomous dysfunction)<br>• diagnosis of chronic inflammatory bowel diseases, celiac disease or colorectal cancer according to the guidelines of the German Society of Gastroenterology<br>• use of anti-psychotic drugs<br>• antibiotic use during the previous 12 months

• consent to specimen collection and specimen use	• start of novel therapy with disease-modifying anti-rheumatic drugs<br>• pregnancy or breastfeeding women<br>• contraindication for additional blood draws (e.g. haemoglobin <10)<br>• BMI < 18.5<br>• Psychiatric illness that limits understanding of the examination protocol (unable to consent)

Fasting dietary counselling

The fasting group is closely monitored by nutritionists trained in fasting therapy, backed up by physicians experienced in fasting, from the Charité – Universitätsmedizin Berlin and the Paracelsus-Elena Clinic to ensure a uniform implementation of the fasting guidelines and the well-being of the study participants. The monitoring consists of several in person and virtual meetings which held individually or in group settings. Five meetings including the visits T0 and T2 during the fasting week as well as a group meeting after PF to ensure a well-managed start to the TRE phase take place. Group sessions are standardised using a pre-set deck of slides to be discussed during the group meetings with only minor disease-related differences between the PD and RA groups. All longitudinal participants receive a study-specific script with information on fasting procedures.

Medication

The medical treatments of the patients are monitored and documented with every clinical visit. The fasting intervention might necessitate temporary adjustments of several medications e.g. anti-diabetic and anti-hypertensive drugs [28].

Data collection

Sample and data collection is performed at the two clinical sites, Charité – Universitätsmedizin Berlin and Paracelsus-Elena Clinic (Table 2).

Table 2: Sampling procedures.

a) Biochemical samples and procedures

Blood (123 mL at T0, 23 mL at T2-T11)
Stool collection (2 mL at T0 and T3-T11)
Saliva collection (3.5 mL at T0-T11)
Midstream urine (50 mL at T0 -T11)

b) Questionnaires

Disease specific
PD:

For peer review only

- Disease Activity Score (DAS-28) [39]
- Parkinson's Disease Sleep Scale-2 (PDSS-2) [40]
- Parkinson's Disease Questionnaire-39 (PDQ-39)[41]
- Simplified Disease Index Score (SDAI) [42]
- Funktionsfragebogen Hannover (FFbH-R) [43]
- Movement Disorder Society Unified PD Rating Scale (MDS-UPDRS)[44]
- Non-Motor Symptoms Questionnaire (NMSQ)[45]
- Non-Motor Symptoms Scale (NMSS)[46]

RA:

- Disease Activity Score (DAS-28) [42]
- Non-Motor Symptoms Questionnaire (NMSQ) [45]
- Funktionsfragebogen Hannover (FFbH-R) [43]

Dietary behaviour and lifestyle

- Fasting experience, expectation, and intervention
- Lifestyle
- 24H-Food-recall
- Food Frequency Questionnaire (FFQ)

General health and well-being

- Health Assessment Questionnaire (HAQ)[47]
- Bristol Stool Scale[48]
- Quality of Life questionnnaire (WHO-5)[49]
- Hospital Anxiety and Depression Scale (HADS)[50]
- Profile of Mood States[51]

For peer review only

Anthropometric data and questionnaires

The electronic data capture system REDCap[52], a secure web-based application, is used to record all individual specific data. All data is stored on a secure server infrastructure at the host institution in Luxembourg. Weight, height, body mass index (BMI), heart rate and blood pressure in sitting and standing position as well as waist-hip-ratio is determined at every visit.

Dietary behaviour, sociodemographic measurements (age, sex, education level, employment status, marital status), family history, current and previous illness and co-morbidities, and current medications, as well as disease-specific data, questionnaires about the well-being of the patients and data on the behavioural factors are collected at baseline, T6 (week 3), T9 (month 6) and T11 (month 12) (Table 2). Questionnaires (24h-Food Recall, Bristol Stool Scale) are answered at all visits by the study participants. Data storage, analysis and exchange are done only in pseudonymised fashion. The nutritional data is analysed using the Nutrilog 3.20 software (Nutrilog SAS, Marans).

Blood samples and parameters

Blood samples are collected at each visit, and immediately used for peripheral blood mononuclear cell (PBMC) isolation (T0), analysis by the study laboratory and centrifugation to freeze plasma samples at -80°C (T0-T11). A clinical standard laboratory report is generated after every visit for each study participant (Table 3). In addition to the routine blood parameters, anti-citrullinated protein antibody (ACPA), zonulin, fatty-acid binding protein 2 (FABP2), and calprotectin are measured. Aliquots are securely stored to account for novel observations qnd testing of hypotheses.

Table 3: Routine blood parameters measured at each timepoint (T0 for cross-sectional study, T0-T11 for longitudinal study)

Haematology - EDTA-blood	Clinical Chemistry - Serum
Basophils, %	Albumin
Basophils, abs.	ALT, 37°C
Eosinophils, %	Alkaline Phosphatase, 37°C
Eosinophils, abs.	AST, 37°C
Erythrocytes	Bilirubin, total
Haematocrit	Cholinesterase
Haemoglobin	Cholesterol
HbA1c	Creatinine
Leucocytes	hs-CRP
Lymphocytes, %	Glucose, serum
Lymphocytes, abs.	Gamma-GT, 37°C
MCH	HDL-Cholesterol
MCHC	LDL-Cholesterol
MCV	Potassium
Monocytes, %	Sodium
Monocytes, abs.	Total Protein
Neutrophils, %	Triglycerides

For peer review only

Neutrophils, abs.	Uric Acid
Platelets	Urea/BUN
RDW	**Proteins - Serum**
Reticulocytes	Rheumatoid factor H 35.9
Reticulocytes	**Hormones - Serum**
Reticulocytes, abs.	Insulin
	TSH (basal)

Stool, urine and saliva samples

The samples listed in Table 2 are collected at each visit, except for stool samples on T2 (fasting week) and immediately frozen and stored at -80°C. Stool characteristics are recorded at the time of the sampling. Faecal samples represent the main sample type for resolving the dynamic processes driven by microbiome in the gut. Also, as the gut microbiome is prone to diurnal fluctuations, the stool samples are, as far as possible, collected in the morning.

Methods applied to samples

Biomolecular extractions

The collected stool samples undergo a biomolecular extraction procedure to allow isolation of concomitant DNA, RNA, proteins, peptides and metabolites from single, unique faecal water samples[53, 54]. Faecal water is recovered following centrifugation and filtration, at low-speed or low-flow, respectively, to avoid cell lysis. Nucleic acids are preserved by the addition of ribonuclease inhibitors and isolated by silica-column-based techniques. This protocol involves the use of a robotic platform, ensuring a higher level of standardisation and reproducibility[2].

Coupled metagenomic and metatranscriptomic analyses

Prior to sequencing library preparation, internal standards are introduced to obtain quantitative sequencing data[55]. Contamination-free metagenomic (MG) and metatranscriptomic (MT) data is generated, processed and analysed using the Integrated Meta-omics Pipeline (IMP)[36], which incorporates pre-processing, assembly, gene annotation, mapping of reads, single nucleotide polymorphism calling, data normalisation as well as analyses of community structure and function in a fully reproducible software framework based on Docker. The MG and MT data is specifically screened for enrichments in genes and pathways with known immunogenic properties[56]. The extracellular biomolecules are linked to specific microbial populations based on the intracellular metagenomic data [57]. In addition, the sequencing data is mapped against genomes of food components[58]. The quantitative data is also related to microbial population sizes to determine the contribution of the resolved microbial populations in stool to the extracellular DNA and RNA complements[59].

For peer review only

Metaproteomics

For the metaproteomic analyses, filtration is used to separate extracellular peptides from the obtained (poly)peptides. The resulting smaller fractions are then desalted and analysed without proteolytic digestion via liquid chromatography (LC) and mass spectrometry (MS) on an EasyNano-LC coupled online to a QExactive-Plus mass spectrometer (ThermoScientific, USA). The identification of ribosomal peptides is done with an integrated catalogue of MG and MT data, while the non-ribosomal peptides are identified using different tools, i.e., MyriMatch, DirecTag as well as CycloBranch[36, 60, 61]. The metaproteomic data also allows identification of extracellular (poly)peptides with possible pathogenic functions including protein misfolding and molecular mimicry[62, 63].

Metabolomics

Metabolomic data is analysed using a combination of targeted and untargeted approaches [54, 58, 64]. This highlights the major metabolite classes produced by the gut microbiome with an effect on human physiology including organic acids, short-chain fatty acids, lipids, branched-chain fatty acids, branched-chain amino acids, vitamins, bile acids and neurotransmitters. Besides external compound calibration series for quantification and quality control samples to ensure data normalisation and data acquisition quality assessment, the metabolite extraction fluid is fortified with multiple internal standards to improve method precision and accuracy[65, 66]. The data is compared to in-house databases and public mass spectral libraries to identify known metabolites. The metabolomic data complements the metagenomic and metatranscriptomic data and thus allows further establishments of conclusive links to metabolic properties in the gut.

Deep immune profiling

Deep immune profiling is done using a recently established and optimised panel of metal-labelled antibodies together with cytometry coupled to mass spectrometry (MS), the Maxpar Direct Immune Profiling System (MDIPA). This approach allows the simultaneous quantification of 38 parameters on single cells. Whole blood is stained with the MDIPA kit and stabilised with Proteomic stabiliser Prot-1 (501351694, Smart Tube Inc., Las Vegas) before storage at -80°C. The quantified immune cells included in the MDIPA panel are CD3+, CD4+, CD8+, monocytes, dendritic cells, granulocytes, MAIT, T cells, NK and B cells[67]. Cytokine expression profiles is analysed on blood plasma using the Human Luminex performance Cytokine Panel (R&D Systems Europe, Abingdon), measuring CCL3, CCL4, CCL5, GM-CSF, IL-1β, IL-2, IL-4, IL-5, IL-6, IL-8, IL-10, IL-12p70, IL-13, IL-15, IL-18, IL-21, IL-27, IL-33, IFN-β, Galectin-1, IFN-γ and TNF-α [56].

Gut-on-a-chip models

PBMCs isolated from T0 blood samples are co-cultured with gut-derived microbes under physiologically representative conditions using the gut-on-a-chip model HuMiX[68]. This model of the human gastrointestinal interface allows the investigation of the interactions between immune, epithelial and bacterial cells and specifically the response to fasting in personalised in vitro models.

For peer review only - http://bmjopen.bmj.com/site/about/guidelines.xhtml

The Expobiome Map

The Expobiome Map (https://expobiome.lcsb.uni.lu) illustrates the diverse complex of microbial immunogenic molecules, including nucleic acids, (poly)peptides, structural molecules, and metabolites. The interactions between this “expobiome” and human immune pathways are encoded in the context of chronic diseases[1]. The ExpoBiome Map is visualised using the MINERVA Platform[69]. Clicking on different elements on the map reveals factors they affect and are affected by, allowing an easier navigation through the complex relationships between individual microbiome components in relation to human disease. The multi-omics data generated in the present study will be integrated with the Map.

Exploratory analysis of novel host-microbiome interactions

Unknown non-ribosomal peptides or metabolite features are associated through correlation with transcripts, proteins and metabolites. Extracellular DNA fragments, as well as transcripts, proteins and ribosomal peptides are linked to their genomic context by using IMP[36]. The data generated by the project will be connected and collated to existing, publicly available datasets.

Outcome parameters

Primary Outcome

The primary endpoint of the study is the characterisation of the gut microbiome. The evaluation includes both between-group and within-group differences in the longitudinal study arms with the fasting intervention.

Secondary Outcome Measures

Secondary outcomes include the identification of common and disease-specific molecular signatures and the characterisation of microbiome-derived effector molecules impacting the innate and adaptive immune pathways. Furthermore, several additional parameters mentioned in *Anthropometric data and questionnaires* are assessed over a period of 12 months.

Sample size and power calculation

A power calculation using pilot metatranscriptomic data based on faecal extracellular RNA samples was performed to determine the number of subjects to be recruited for the ExpoBiome project. The obtained relative abundances of genera were used for the calculation of the required sample size per group. The power calculation was based on the algorithm as described by Tusher, Tibshirani, and Chu[70]. To achieve a power of 90% (at $\alpha = 0.05$), a total of 50 individuals per group (RA, PD, healthy controls) must be analysed. Considering any possible dropouts, 20% additional subjects are recruited, resulting in a total number of 180 individuals, i.e., 60 per group.

For peer review only

Adverse events

There are no major risks expected for participants. Minor common adverse effects of PF might include headaches, nausea, insomnia, back pain, dyspepsia and fatigue[71]. Any occurring adverse events are recorded at each visit in REDCap[52]. Serious adverse events are communicated to the study coordinator and principal investigator within 24 h of their report.

Data management, monitoring, analysis and evaluation of data

The study participants receive a study ID (pseudonym) which is used for all collected data. Self-administered questionnaires are directly recorded in REDCap. Participant files are kept for at least 10 years at the respective clinical sites.

Weekly meetings between the study team, the different clinical partners and the principal investigator, ensure a close monitoring of the data. Any occurring adverse events or other issues are thus handled immediately.

Different statistical tests are performed according to the nature of the data. A premature termination of the study is not envisaged; therefore, no interim analysis is done. Different correlation measures are applied, including Spearman correlation, mutual information on discretised data, distance correlation, maximum information criterion, local similarity analysis and the bioenv approach. Comparison across all omic levels allows identification of common and disease-specific signatures. Multivariate machine learning is used to link different data features to observed patterns.

The longitudinal part of the study continues for a period of 12 months. After finalisation of this period, there is no follow-up of the participants. Interesting findings will be further validated using the existing sample set and analyses may be performed on additionally collected samples.

Discussion (869)

The impact and importance of the gut microbiome on human physiology and its potential modifications by nutrition and dietary patterns, have been underestimated for centuries[72].

More recently, physical as well as mental well-being has been related to the gut microbiome composition and new associations to human health are emerging almost every day. The gut microbiome is responsible for the fermentation of indigestible food components, the synthesis of numerous metabolites including vitamins, the removal of toxic compounds and pathogens, the maintenance and strengthening of the intestinal barrier and immune system function amongst others[21].

Globally we observe a gradual shift to a Western diet, typically low in micronutrients, mono- and polyunsaturated fats and linoleic acid but high in saturated fat, refined carbohydrates, salt and processed food. This shift is associated with an emergence of non-communicable diseases (NCDs) such as obesity, cardiovascular diseases, type 2 diabetes mellitus, cancer, liver and gastrointestinal diseases, bone and joint diseases, and degenerative diseases of the brain[73]. Not surprisingly, obesity and diabetes were some of the first diseases to be linked to the gut microbiome composition[74]. Psychological disorders like anxiety and depression have also been associated with the gut microbiome[75], emphasizing the importance of the bacterial community in various facets of health.

For peer review only

Fasting has been used as an intervention to promote health or to heal diseases since the beginning of civilisations and has spread independently among different regions, cultures and religions worldwide[76]. It is believed to have already been established as a treatment method by Hippocrates in the 5th century BCE and has been used ever since by numerous medical schools to treat acute and chronic diseases[27, 77]. Various practices of caloric restriction through fasting have repeatedly shown remarkable health benefits[78, 79]. Maifeld *et al.* for instance found that a 5-day fast followed by a modified Dietary Approach to Stop Hypertension (DASH), with additional emphasis on plant-based and Mediterranean diets, reduced systolic blood pressure, BMI, and the need for antihypertensive medications at three months post intervention compared to DASH alone. Bacterial taxa and genes associated with short-chain fatty acid production were altered in the gut, e.g. many Clostridial Firmicutes shifted significantly in abundance, with an initial decrease in butyrate producers such as *Faecalibacterium prausnitzii* [34].

Furthermore, Choi *et al.* demonstrated that cycles of a fasting-mimicking diet suppress autoimmunity and stimulate remyelination via oligodendrocyte regeneration in a murine experimental autoimmune encephalomyelitis (EAE) model[80]. Jordan *et al.* described a reduction in monocyte metabolic and inflammatory activity after a short-term fast and conclude that fasting attenuates chronic inflammatory diseases without compromising monocyte capacity for mobilisation during acute infectious inflammation and tissue repair[81].

Diet as a tool to prevent disease, considering both the quantity and the quality of food, remains underappreciated in routine clinical practice as well as in research. Reasons may include missing standardised therapeutic protocols, the interindividual variability in the response to fasting, lack of knowledge about possible adverse effects, and difficulties in the interpretation of underlying mechanisms seen in clinical trials, but also in the comparably low potential for achieving economic revenue or scientific impact[8].

Modern experimental approaches and computational integration allow a multi-layer analysis of digestive processes in low caloric settings including the gut microbiome[58]. These technological developments also permit a closer investigation of the link between the immune system and severe caloric restriction.

Fasting-induced changes in the gut microbiome are associated with host energy metabolism[33], with a shift in the gut microbiome after as little as 24h[72]. Although the changes in gut microbiome composition induced by a 10-day PF of healthy individuals return to baseline after 3 months, the resilience of the initial gut microbiome composition might not be the same in unhealthy individuals[33]. Currently available knowledge is insufficient to define a healthy gut microbiome due to high interindividual variability. However, several characteristics such as increased diversity in microbial taxa and their gene richness, a high amount of butyrate producers and resilience of the microbial community are often considered as beneficial[82]. Thus, a dysbiotic gut microbiome is likely to be less resilient and therefore more susceptible to dietary interventions, enabling a possible reversion to a healthy status[72].

For peer review only

A potential mechanism underlying the observed beneficial effects induced by dietary interventions might be a direct gut microbiome-immune system interaction by pattern recognition. The microbiome can regulate the intestinal innate immune system by modulating toll-like receptor (TLR) expression on immunosensor cell surface through microbe-associated molecular patterns (MAMPs), which can consequently trigger cytokine production and up-regulation of molecules on antigen presenting cells, leading to activation of T cells[83]. Therefore, a change in gut microbiome composition can lead to different outcomes in immune signaling pathways and either favor or suppress inflammation and autoimmunity.

The current evidence highlights the immensely important connection between the environment and health, including dietary habits, microbiome and the immune system. Combining the newly generated data of this study with knowledge from disciplines such as nutrigenomics could potentially lead to more extended and deepened utilisation of personalised nutrition. Taking the gut microbiome into account as a key factor in disease for nutritional recommendations might revolutionise the current guidelines and allow substantially more efficient proposals. These approaches might eventually be broadly applicable and constitute an efficient antidote against the multiple burden of malnutrition and over-alimentation and the prominent NCDs.

Trial status

The recruitment for the ExpoBiome study started in April 2021 and is currently ongoing. All study participants should be recruited by the end of 2022.

Acknowledgements

We thank Dr. Catharina Delebinski, Melanie Dell'Oro, Grit Langhans, Ursula Reuß, Maik Schröder and Nadine Sylvester for their support during the study.

Author contributions:

- Study design and protocol: Bérénice Hansen, Cédric C. Laczny, Jochen G. Schneider, Paul Wilmes
- Interventional concept: Etienne Hanslian, Daniela Liebscher, Andreas Michalsen, Brit Mollenhauer, Sebastian Schade, Jochen G. Schneider, Paul Wilmes
- Procured funding: Paul Wilmes
- Statistical planning, sample size calculation and randomisation: Cédric C. Laczny, Jochen G. Schneider, Paul Wilmes
- Initial draft of the manuscript and coordinated the editing process: Bérénice Hansen
- Contributed equally with edits, comments and feedback, read and approved the final manuscript: all authors

Funding statement: This project has received funding from the European Research Council (ERC) under the European Union's Horizon 2020 research and innovation program (grant agreement No. 863664).

For peer review only

Competing interests statement

None declared.

Supplements

We used the SPIRIT checklist when writing our report[84].

References

1. Wilmes, P., et al., *The gut microbiome molecular complex in human health and disease.* Cell Host Microbe, 2022. **30**(9): p. 1201-1206.
2. De Saedeleer, B., et al., *Systematic characterization of human gut microbiome-secreted molecules by integrated multi-omics.* ISME Communications, 2021. **1**(1): p. 82.
3. Greenhalgh, K., et al., *The human gut microbiome in health: establishment and resilience of microbiota over a lifetime.* Environ Microbiol, 2016. **18**(7): p. 2103-16.
4. Hall, A.B., A.C. Tolonen, and R.J. Xavier, *Human genetic variation and the gut microbiome in disease.* Nature Reviews Genetics, 2017. **18**(11): p. 690-699.
5. Baldini, F., et al., *Parkinson's disease-associated alterations of the gut microbiome predict disease-relevant changes in metabolic functions.* BMC Biol, 2020. **18**(1): p. 62.
6. Yoo, J.Y., et al., *Gut Microbiota and Immune System Interactions.* Microorganisms, 2020. **8**(10).
7. Sonnenburg, J.L. and F. Bäckhed, *Diet–microbiota interactions as moderators of human metabolism.* Nature, 2016. **535**(7610): p. 56-64.
8. Zmora, N., J. Suez, and E. Elinav, *You are what you eat: diet, health and the gut microbiota.* Nat Rev Gastroenterol Hepatol, 2019. **16**(1): p. 35-56.
9. Guo, Q., et al., *Rheumatoid arthritis: pathological mechanisms and modern pharmacologic therapies.* Bone Res, 2018. **6**: p. 15.
10. Healthline, V.L. *Rheumatoid Arthritis by the Numbers: Facts, Statistics, and You*. 2021.
11. Scherer, H.U., T. Häupl, and G.R. Burmester, *The etiology of rheumatoid arthritis.* Journal of Autoimmunity, 2020. **110**: p. 102400.
12. Bodkhe, R., B. Balakrishnan, and V. Taneja, *The role of microbiome in rheumatoid arthritis treatment.* Ther Adv Musculoskelet Dis, 2019. **11**: p. 1759720x19844632.
13. Chen, J., et al., *An expansion of rare lineage intestinal microbes characterizes rheumatoid arthritis.* Genome Medicine, 2016. **8**(1): p. 43.
14. Wu, H.J., et al., *Gut-residing segmented filamentous bacteria drive autoimmune arthritis via T helper 17 cells.* Immunity, 2010. **32**(6): p. 815-27.
15. Scher, J.U., et al., *Expansion of intestinal Prevotella copri correlates with enhanced susceptibility to arthritis.* eLife, 2013. **2**: p. e01202.
16. Zhang, X., et al., *The oral and gut microbiomes are perturbed in rheumatoid arthritis and partly normalized after treatment.* Nature Medicine, 2015. **21**(8): p. 895-905.
17. Lubomski, M., et al., *Parkinson's disease and the gastrointestinal microbiome.* J Neurol, 2020. **267**(9): p. 2507-2523.
18. Opara, J., et al., *Motor assessment in Parkinson`s disease.* Ann Agric Environ Med, 2017. **24**(3): p. 411-415.

For peer review only

19. Tysnes, O.B. and A. Storstein, *Epidemiology of Parkinson's disease.* J Neural Transm (Vienna), 2017. **124**(8): p. 901-905.

20. Garcia, P., et al., *Neurodegeneration and neuroinflammation are linked, but independent of alpha-synuclein inclusions, in a seeding/spreading mouse model of Parkinson's disease.* Glia, 2022. **70**(5): p. 935-960.

21. Heintz-Buschart, A. and P. Wilmes, *Human Gut Microbiome: Function Matters.* Trends Microbiol, 2018. **26**(7): p. 563-574.

22. Romano, S., et al., *Meta-analysis of the Parkinson's disease gut microbiome suggests alterations linked to intestinal inflammation.* npj Parkinson's Disease, 2021. **7**(1): p. 27.

23. Mertsalmi, T.H., et al., *More than constipation - bowel symptoms in Parkinson's disease and their connection to gut microbiota.* European journal of neurology, 2017. **24**(11): p. 1375-1383.

24. Dogra, N., R.J. Mani, and D.P. Katare, *The Gut-Brain Axis: Two Ways Signaling in Parkinson's Disease.* Cell Mol Neurobiol, 2022. **42**(2): p. 315-332.

25. Forsyth, C.B., et al., *Increased intestinal permeability correlates with sigmoid mucosa alpha-synuclein staining and endotoxin exposure markers in early Parkinson's disease.* PLoS One, 2011. **6**(12): p. e28032.

26. Devos, D., et al., *Colonic inflammation in Parkinson's disease.* Neurobiol Dis, 2013. **50**: p. 42-8.

27. Britannica, *The Editors of Encyclopaedia. "fasting".* Encyclopedia Britannica,, Accessed 3 October 2022. https://www.britannica.com/topic/fasting.

28. Hartmann, A.M., et al., *Efficacy of therapeutic fasting and plant-based diet in patients with rheumatoid arthritis (NutriFast): study protocol for a randomised controlled clinical trial.* BMJ Open, 2021. **11**(8): p. e047758.

29. Hartmann, A.M., et al., *To eat or not to eat—an exploratory randomized controlled trial on fasting and plant-based diet in rheumatoid arthritis (NutriFast-Study).* Frontiers in Nutrition, 2022. **9**.

30. Sköldstam, L., *Fasting and vegan diet in rheumatoid arthritis.* Scand J Rheumatol, 1986. **15**(2): p. 219-21.

31. Neth, B.J., et al., *The Role of Intermittent Fasting in Parkinson's Disease.* Front Neurol, 2021. **12**: p. 682184.

32. Cignarella, F., et al., *Intermittent Fasting Confers Protection in CNS Autoimmunity by Altering the Gut Microbiota.* Cell Metab, 2018. **27**(6): p. 1222-1235.e6.

33. Mesnage, R., et al., *Changes in human gut microbiota composition are linked to the energy metabolic switch during 10 d of Buchinger fasting.* J Nutr Sci, 2019. **8**: p. e36.

34. Maifeld, A., et al., *Fasting alters the gut microbiome reducing blood pressure and body weight in metabolic syndrome patients.* Nature Communications, 2021. **12**(1): p. 1970.

35. Magne, F., et al., *The Firmicutes/Bacteroidetes Ratio: A Relevant Marker of Gut Dysbiosis in Obese Patients?* Nutrients, 2020. **12**(5).

36. Narayanasamy, S., et al., *IMP: a pipeline for reproducible reference-independent integrated metagenomic and metatranscriptomic analyses.* Genome Biology, 2016. **17**(1): p. 260.

37. Wilmes, P., A. Heintz-Buschart, and P.L. Bond, *A decade of metaproteomics: where we stand and what the future holds.* Proteomics, 2015. **15**(20): p. 3409-17.

For peer review only

38. Gabel, K., et al., *Effects of 8-hour time restricted feeding on body weight and metabolic disease risk factors in obese adults: A pilot study.* Nutr Healthy Aging, 2018. **4**(4): p. 345-353.
39. Wells G, B.J., Teng J, et al, *Validation of the 28-joint Disease Activity Score (DAS28) and European League Against Rheumatism response criteria based on C-reactive protein against disease progression in patients with rheumatoid arthritis, and comparison with the DAS28 based on erythrocyte sedimentation rate.* Annals of the Rheumatic Diseases 2009. **68**: p. 954-960.
40. Trenkwalder, C., et al., *Parkinson's disease sleep scale--validation of the revised version PDSS-2.* Mov Disord, 2011. **26**(4): p. 644-52.
41. Bushnell, D.M. and M.L. Martin, *Quality of life and Parkinson's disease: translation and validation of the US Parkinson's Disease Questionnaire (PDQ-39).* Qual Life Res, 1999. **8**(4): p. 345-50.
42. Smolen, J.S., et al., *A simplified disease activity index for rheumatoid arthritis for use in clinical practice.* Rheumatology (Oxford), 2003. **42**(2): p. 244-57.
43. Raspe, H.H., Hagedorn, U., Kohlmann, T., & Mattussek, S., *Der Funktionsfragebogen Hannover (FFbH): Ein Instrument zur Funktionsdiagnostik bei polyartikulären Gelenkerkrankungen*, in *Ergebnisse sozialwissenschaftlicher Evaluation eines Modellversuchs (pp. 164-182)*. 1990, Schattauer Verlag.
44. Goetz, C.G., et al., *Movement Disorder Society-sponsored revision of the Unified Parkinson's Disease Rating Scale (MDS-UPDRS): Process, format, and clinimetric testing plan.* Mov Disord, 2007. **22**(1): p. 41-7.
45. Chaudhuri, K.R., et al., *International multicenter pilot study of the first comprehensive self-completed nonmotor symptoms questionnaire for Parkinson's disease: the NMSQuest study.* Mov Disord, 2006. **21**(7): p. 916-23.
46. Chaudhuri, K.R., et al., *The metric properties of a novel non-motor symptoms scale for Parkinson's disease: Results from an international pilot study.* Mov Disord, 2007. **22**(13): p. 1901-11.
47. Wolfe, F., *A brief clinical health assessment instrument clinhaq.* Arthritis and Rheumatism, 1989. **32**(4 Suppl): p. S99-S99.
48. Lewis, S.J. and K.W. Heaton, *Stool form scale as a useful guide to intestinal transit time.* Scand J Gastroenterol, 1997. **32**(9): p. 920-4.
49. Topp, C.W., et al., *The WHO-5 Well-Being Index: a systematic review of the literature.* Psychother Psychosom, 2015. **84**(3): p. 167-76.
50. Zigmond, A.S. and R.P. Snaith, *The hospital anxiety and depression scale.* Acta Psychiatr Scand, 1983. **67**(6): p. 361-70.
51. McNair DM, L.M., Droppleman LF, *Edits Manual for the Profile of Mood States (Poms). Rev ed.* . San Diego: Educational and Industrial Testing Service, 1992.
52. Harris, P.A., et al., *Research electronic data capture (REDCap)--a metadata-driven methodology and workflow process for providing translational research informatics support.* J Biomed Inform, 2009. **42**(2): p. 377-81.
53. Wilmes, P., Roume, H., Hiller, K. & Cordes, T., *Method and kit for the isolation of genomic DNA, RNA, proteins and metabolites from a single biological sample.*, in *World Intellectual Property Organization*, C.D.R.P.-G.L. Université Du Luxembourg, Editor. 2014: Switzerland.
54. Roume, H., et al., *A biomolecular isolation framework for eco-systems biology.* The ISME Journal, 2013. **7**(1): p. 110-121.

For peer review only

55. Locati, M.D., et al., *Improving small RNA-seq by using a synthetic spike-in set for size-range quality control together with a set for data normalization.* Nucleic Acids Res, 2015. **43**(14): p. e89.

56. Wampach, L., et al., *Birth mode is associated with earliest strain-conferred gut microbiome functions and immunostimulatory potential.* Nature Communications, 2018. **9**(1): p. 5091.

57. Albanese, D. and C. Donati, *Strain profiling and epidemiology of bacterial species from metagenomic sequencing.* Nature Communications, 2017. **8**(1): p. 2260.

58. Heintz-Buschart, A., et al., *Integrated multi-omics of the human gut microbiome in a case study of familial type 1 diabetes.* Nature Microbiology, 2016. **2**(1): p. 16180.

59. Vandeputte, D., et al., *Quantitative microbiome profiling links gut community variation to microbial load.* Nature, 2017. **551**(7681): p. 507-511.

60. Tang, H., S. Li, and Y. Ye, *A Graph-Centric Approach for Metagenome-Guided Peptide and Protein Identification in Metaproteomics.* PLoS Comput Biol, 2016. **12**(12): p. e1005224.

61. Tabb, D.L., C.G. Fernando, and M.C. Chambers, *MyriMatch: highly accurate tandem mass spectral peptide identification by multivariate hypergeometric analysis.* J Proteome Res, 2007. **6**(2): p. 654-61.

62. Heintz-Buschart, A., et al., *The nasal and gut microbiome in Parkinson's disease and idiopathic rapid eye movement sleep behavior disorder.* Mov Disord, 2018. **33**(1): p. 88-98.

63. Chen, S.G., et al., *Exposure to the Functional Bacterial Amyloid Protein Curli Enhances Alpha-Synuclein Aggregation in Aged Fischer 344 Rats and Caenorhabditis elegans.* Scientific Reports, 2016. **6**(1): p. 34477.

64. Wilmes, P., et al., *Metabolome-proteome differentiation coupled to microbial divergence.* mBio, 2010. **1**(5).

65. Kim, D.H., et al., *LC-MS-based absolute metabolite quantification: application to metabolic flux measurement in trypanosomes.* Metabolomics, 2015. **11**(6): p. 1721-1732.

66. Lei, Z., D.V. Huhman, and L.W. Sumner, *Mass spectrometry strategies in metabolomics.* J Biol Chem, 2011. **286**(29): p. 25435-42.

67. *Immunophenotyping assessment in a COVID-19 cohort (IMPACC): A prospective longitudinal study.* Sci Immunol, 2021. **6**(62).

68. Shah, P., et al., *A microfluidics-based in vitro model of the gastrointestinal human–microbe interface.* Nature Communications, 2016. **7**(1): p. 11535.

69. Aho, V.T.E., et al., *SnapShot: The Expobiome Map.* Cell Host Microbe, 2022. **30**(9): p. 1340-1340.e1.

70. Tusher, V.G., R. Tibshirani, and G. Chu, *Significance analysis of microarrays applied to the ionizing radiation response.* Proc Natl Acad Sci U S A, 2001. **98**(9): p. 5116-21.

71. Finnell, J.S., et al., *Is fasting safe? A chart review of adverse events during medically supervised, water-only fasting.* BMC Complementary and Alternative Medicine, 2018. **18**(1): p. 67.

72. Leeming, E.R., et al., *Effect of Diet on the Gut Microbiota: Rethinking Intervention Duration.* Nutrients, 2019. **11**(12): p. 2862.

73. Isaza, A., R. Singh, and S. Watanabe, *Functional Foods and Nutraceuticals in Metabolic and Non-communicable Diseases*. 2021.

For peer review only

74. Duranti, S., et al., *Obesity and microbiota: an example of an intricate relationship.* Genes Nutr, 2017. **12**: p. 18.
75. Simpson, C.A., et al., *The gut microbiota in anxiety and depression - A systematic review.* Clin Psychol Rev, 2021. **83**: p. 101943.
76. Kerndt, P.R., et al., *Fasting: the history, pathophysiology and complications.* West J Med, 1982. **137**(5): p. 379-99.
77. Michalsen, A., *Prolonged fasting as a method of mood enhancement in chronic pain syndromes: a review of clinical evidence and mechanisms.* Curr Pain Headache Rep, 2010. **14**(2): p. 80-7.
78. Vargas-Molina, S., et al., *Effects of a low-carbohydrate ketogenic diet on health parameters in resistance-trained women.* Eur J Appl Physiol, 2021. **121**(8): p. 2349-2359.
79. Mattison, J.A., et al., *Caloric restriction improves health and survival of rhesus monkeys.* Nature Communications, 2017. **8**(1): p. 14063.
80. Choi, I.Y., et al., *A Diet Mimicking Fasting Promotes Regeneration and Reduces Autoimmunity and Multiple Sclerosis Symptoms.* Cell Rep, 2016. **15**(10): p. 2136-2146.
81. Jordan, S., et al., *Dietary Intake Regulates the Circulating Inflammatory Monocyte Pool.* Cell, 2019. **178**(5): p. 1102-1114.e17.
82. Dogra, S.K., J. Doré, and S. Damak, *Gut Microbiota Resilience: Definition, Link to Health and Strategies for Intervention.* Front Microbiol, 2020. **11**: p. 572921.
83. Purchiaroni, F., et al., *The role of intestinal microbiota and the immune system.* Eur Rev Med Pharmacol Sci, 2013. **17**(3): p. 323-33.
84. Chan, A.-W., et al., *SPIRIT 2013 explanation and elaboration: guidance for protocols of clinical trials.* BMJ : British Medical Journal, 2013. **346**: p. e7586.

Figure Legends

Figure 1. Study design. This figure illustrates the study design with five different arms in total, two of which continue with the longitudinal part of the study. Visits take place at the clinical sites at each timepoint and include the collection of the displayed samples. This image was generated using Biorender software (http://www.biorender.com). T, timepoint; W, week; D, day; M, month.

For peer review only

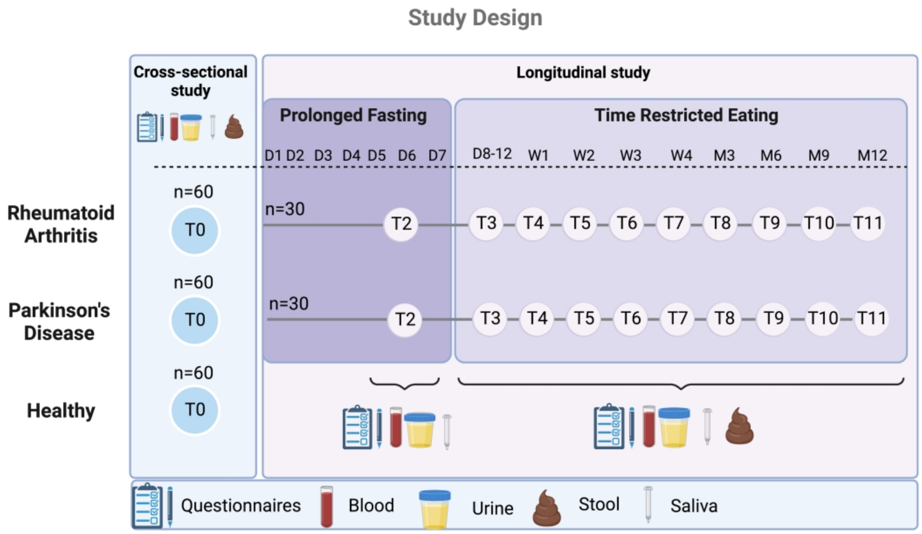

Figure 1. Study design. This figure illustrates the study design with five different arms in total, two of which continue with the longitudinal part of the study. Visits take place at the clinical sites at each timepoint and include the collection of the displayed samples. This image was generated using Biorender software (http://www.biorender.com). T, timepoint; W, week; D, day; M, month.

279x177mm (300 x 300 DPI)

Reporting checklist for protocol of a clinical trial.

Based on the SPIRIT guidelines.

Instructions to authors

Complete this checklist by entering the page numbers from your manuscript where readers will find each of the items listed below.

Your article may not currently address all the items on the checklist. Please modify your text to include the missing information. If you are certain that an item does not apply, please write "n/a" and provide a short explanation.

Upload your completed checklist as an extra file when you submit to a journal.

In your methods section, say that you used the SPIRITreporting guidelines, and cite them as:

Chan A-W, Tetzlaff JM, Gøtzsche PC, Altman DG, Mann H, Berlin J, Dickersin K, Hróbjartsson A, Schulz KF, Parulekar WR, Krleža-Jerić K, Laupacis A, Moher D. SPIRIT 2013 Explanation and Elaboration: Guidance for protocols of clinical trials. BMJ. 2013;346:e7586

		Reporting Item	Page Number
**Administrative information**			
Title	#1	Descriptive title identifying the study design, population, interventions, and, if applicable, trial acronym	1
Trial registration	#2a	Trial identifier and registry name. If not yet registered, name of intended registry	2
Trial registration: data set	#2b	All items from the World Health Organization Trial Registration Data Set	n/a
Protocol version	#3	Date and version identifier	n/a
Funding	#4	Sources and types of financial, material, and other support	14
Roles and responsibilities: contributorship	#5a	Names, affiliations, and roles of protocol contributors	1

For peer review only

Roles and responsibilities: sponsor contact information	#5b	Name and contact information for the trial sponsor	1
Roles and responsibilities: sponsor and funder	#5c	Role of study sponsor and funders, if any, in study design; collection, management, analysis, and interpretation of data; writing of the report; and the decision to submit the report for publication, including whether they will have ultimate authority over any of these activities	1, 14
Roles and responsibilities: committees	#5d	Composition, roles, and responsibilities of the coordinating centre, steering committee, endpoint adjudication committee, data management team, and other individuals or groups overseeing the trial, if applicable (see Item 21a for data monitoring committee)	1, 14
**Introduction**			
Background and rationale	#6a	Description of research question and justification for undertaking the trial, including summary of relevant studies (published and unpublished) examining benefits and harms for each intervention	3
Background and rationale: choice of comparators	#6b	Explanation for choice of comparators	2
Objectives	#7	Specific objectives or hypotheses	5
Trial design	#8	Description of trial design including type of trial (eg, parallel group, crossover, factorial, single group), allocation ratio, and framework (eg, superiority, equivalence, non-inferiority, exploratory)	5
**Methods: Participants, interventions, and outcomes**			
Study setting	#9	Description of study settings (eg, community clinic, academic hospital) and list of countries where data will be collected. Reference to where list of study sites can be obtained	6

For peer review only

Eligibility criteria	#10	Inclusion and exclusion criteria for participants. If applicable, eligibility criteria for study centres and individuals who will perform the interventions (eg, surgeons, psychotherapists)	6
Interventions: description	#11a	Interventions for each group with sufficient detail to allow replication, including how and when they will be administered	5
Interventions: modifications	#11b	Criteria for discontinuing or modifying allocated interventions for a given trial participant (eg, drug dose change in response to harms, participant request, or improving / worsening disease)	5
Interventions: adherance	#11c	Strategies to improve adherence to intervention protocols, and any procedures for monitoring adherence (eg, drug tablet return; laboratory tests)	7
Interventions: concomitant care	#11d	Relevant concomitant care and interventions that are permitted or prohibited during the trial	7
Outcomes	#12	Primary, secondary, and other outcomes, including the specific measurement variable (eg, systolic blood pressure), analysis metric (eg, change from baseline, final value, time to event), method of aggregation (eg, median, proportion), and time point for each outcome. Explanation of the clinical relevance of chosen efficacy and harm outcomes is strongly recommended	8
Participant timeline	#13	Time schedule of enrolment, interventions (including any run-ins and washouts), assessments, and visits for participants. A schematic diagram is highly recommended (see Figure)	5
Sample size	#14	Estimated number of participants needed to achieve study objectives and how it was determined, including clinical and statistical assumptions supporting any sample size calculations	11
Recruitment	#15	Strategies for achieving adequate participant enrolment to reach target sample size	5
**Methods: Assignment of interventions (for controlled trials)**			
Allocation: sequence generation	#16a	Method of generating the allocation sequence (eg, computer-generated random numbers), and list of any factors for stratification. To reduce predictability of a random sequence, details of any planned restriction (eg, blocking) should be	n/a

For peer review only

		provided in a separate document that is unavailable to those who enrol participants or assign interventions	
Allocation concealment mechanism	#16b	Mechanism of implementing the allocation sequence (eg, central telephone; sequentially numbered, opaque, sealed envelopes), describing any steps to conceal the sequence until interventions are assigned	n/a
Allocation: implementation	#16c	Who will generate the allocation sequence, who will enrol participants, and who will assign participants to interventions	n/a
Blinding (masking)	#17a	Who will be blinded after assignment to interventions (eg, trial participants, care providers, outcome assessors, data analysts), and how	n/a
Blinding (masking): emergency unblinding	#17b	If blinded, circumstances under which unblinding is permissible, and procedure for revealing a participant's allocated intervention during the trial	n/a
**Methods: Data collection, management, and analysis**			
Data collection plan	#18a	Plans for assessment and collection of outcome, baseline, and other trial data, including any related processes to promote data quality (eg, duplicate measurements, training of assessors) and a description of study instruments (eg, questionnaires, laboratory tests) along with their reliability and validity, if known. Reference to where data collection forms can be found, if not in the protocol	8
Data collection plan: retention	#18b	Plans to promote participant retention and complete follow-up, including list of any outcome data to be collected for participants who discontinue or deviate from intervention protocols	9
Data management	#19	Plans for data entry, coding, security, and storage, including any related processes to promote data quality (eg, double data entry; range checks for data values). Reference to where details of data management procedures can be found, if not in the protocol	12
Statistics: outcomes	#20a	Statistical methods for analysing primary and secondary outcomes. Reference to where other details of the statistical analysis plan can be found, if not in the protocol	12

For peer review only

Statistics: additional analyses	#20b	Methods for any additional analyses (eg, subgroup and adjusted analyses)	12
Statistics: analysis population and missing data	#20c	Definition of analysis population relating to protocol non-adherence (eg, as randomised analysis), and any statistical methods to handle missing data (eg, multiple imputation)	12
**Methods: Monitoring**			
Data monitoring: formal committee	#21a	Composition of data monitoring committee (DMC); summary of its role and reporting structure; statement of whether it is independent from the sponsor and competing interests; and reference to where further details about its charter can be found, if not in the protocol. Alternatively, an explanation of why a DMC is not needed	12
Data monitoring: interim analysis	#21b	Description of any interim analyses and stopping guidelines, including who will have access to these interim results and make the final decision to terminate the trial	n/a
Harms	#22	Plans for collecting, assessing, reporting, and managing solicited and spontaneously reported adverse events and other unintended effects of trial interventions or trial conduct	12
Auditing	#23	Frequency and procedures for auditing trial conduct, if any, and whether the process will be independent from investigators and the sponsor	n/a
**Ethics and dissemination**			
Research ethics approval	#24	Plans for seeking research ethics committee / institutional review board (REC / IRB) approval	2
Protocol amendments	#25	Plans for communicating important protocol modifications (eg, changes to eligibility criteria, outcomes, analyses) to relevant parties (eg, investigators, REC / IRBs, trial participants, trial registries, journals, regulators)	2
Consent or assent	#26a	Who will obtain informed consent or assent from potential trial participants or authorised surrogates, and how (see Item 32)	6

For peer review only

Consent or assent: ancillary studies	#26b	Additional consent provisions for collection and use of participant data and biological specimens in ancillary studies, if applicable	6
Confidentiality	#27	How personal information about potential and enrolled participants will be collected, shared, and maintained in order to protect confidentiality before, during, and after the trial	12
Declaration of interests	#28	Financial and other competing interests for principal investigators for the overall trial and each study site	14
Data access	#29	Statement of who will have access to the final trial dataset, and disclosure of contractual agreements that limit such access for investigators	12
Ancillary and post trial care	#30	Provisions, if any, for ancillary and post-trial care, and for compensation to those who suffer harm from trial participation	n/a
Dissemination policy: trial results	#31a	Plans for investigators and sponsor to communicate trial results to participants, healthcare professionals, the public, and other relevant groups (eg, via publication, reporting in results databases, or other data sharing arrangements), including any publication restrictions	2
Dissemination policy: authorship	#31b	Authorship eligibility guidelines and any intended use of professional writers	14
Dissemination policy: reproducible research	#31c	Plans, if any, for granting public access to the full protocol, participant-level dataset, and statistical code	n/a
**Appendices**			
Informed consent materials	#32	Model consent form and other related documentation given to participants and authorised surrogatess	6
Biological specimens	#33	Plans for collection, laboratory evaluation, and storage of biological specimens for genetic or molecular analysis in the current trial and for future use in ancillary studies, if applicable	7

The SPIRIT Explanation and Elaboration paper is distributed under the terms of the Creative Commons Attribution License CC-BY-NC. This checklist was completed on 07. November 2022 using https://www.goodreports.org/, a tool made by the EQUATOR Network in collaboration with Penelope.ai

For peer review only

BMJ Open

Protocol for a multicentre cross-sectional, longitudinal clinical trial in rheumatoid arthritis and Parkinson's disease patients analysing the relation between the gut microbiome, fasting and immune status (ExpoBiome)

Journal:	*BMJ Open*
Manuscript ID	bmjopen-2022-071380.R1
Article Type:	Protocol
Date Submitted by the Author:	31-May-2023

Complete List of Authors:	Hansen, Bérénice; LCSB<br>Laczny, Cédric C.; LCSB<br>Aho, Velma T.E.; LCSB<br>Frachet-Bour, Audrey; LCSB<br>Habier, Janine; LCSB<br>Ostaszewski, Marek; LCSB<br>Michalsen, Andreas; Charité Universitätsmedizin Berlin, Institute for Social Medicine, Epidemiology and Health Economics; Immanuel Hospital Berlin-Wannsee Branch, Department of Internal and Integrative Medicine<br>Hanslian, Etienne; Charite Universitatsmedizin Berlin, Institute for Social Medicine, Epidemiology and Health Economics; Immanuel Hospital Berlin-Wannsee Branch<br>Koppold-Liebscher, Daniela; Charité Universitätsmedizin Berlin, Institute for Social Medicine, Epidemiology and Health Economics; Immanuel Hospital Berlin-Wannsee Branch<br>Hartmann, Anika; Charité Universitätsmedizin Berlin, Institute of Social Medicine, Epidemiology and Health Economics; Charité Universitätsmedizin Berlin, Department of Dermatology, Venereology and Allergology<br>Steckhan, Nico; Charité Universitätsmedizin Berlin, Institute for Social Medicine, Epidemiology and Health Economics; University of Potsdam, Digital Health - Connected Healthcare, Hasso Plattner Institute<br>Mollenhauer, Brit; University Medical Center Göttingen; Paracelsus-Kliniken Deutschland GmbH<br>Schade, Sebastian; University Medical Center Göttingen; Paracelsus-Kliniken Deutschland GmbH,<br>Roomp, Kirsten; LCSB<br>Schneider, Jochen; LCSB; Saarland University Hospital and Saarland University Faculty of Medicine, Department of Internal Medicine and Psychiatry<br>Wilmes, Paul; LCSB; University of Luxembourg, Department of Life Sciences and Medicine

**Primary Subject Heading**:	Nutrition and metabolism
Secondary Subject Heading:	Immunology (including allergy), Rheumatology, Pharmacology and therapeutics, Neurology, Evidence based practice

Keywords:	IMMUNOLOGY, Rheumatology < INTERNAL MEDICINE, MICROBIOLOGY, Parkinson-s disease < NEUROLOGY, NUTRITION & DIETETICS, Clinical trials < THERAPEUTICS

SCHOLARONE™
Manuscripts

Protocol for a multicentre cross-sectional, longitudinal clinical trial in rheumatoid arthritis and Parkinson's disease patients analysing the relation between the gut microbiome, fasting and immune status (ExpoBiome)

Bérénice Hansen[1], Cédric C. Laczny[1], Velma T.E. Aho[1], Audrey Frachet-Bour[1], Janine Habier[1], Marek Ostaszewski[1], Andreas Michalsen[4,5], Etienne Hanslian[4,5], Daniela A. Koppold[4,5,8], Anika Hartmann[4,10], Nico Steckhan[4,9], Brit Mollenhauer[6,7], Sebastian Schade[6,7], Kirsten Roomp[1], Jochen G. Schneider[1,3+], Paul Wilmes[1,2+]

[1]Luxembourg Centre for Systems Biomedicine, University of Luxembourg, Campus Belval, 7, avenue des Hauts-Fourneaux, L-4362 Esch-sur-Alzette, Luxembourg

[2]Department of Life Sciences and Medicine, University of Luxembourg, 7, avenue des Hauts-Fourneaux, L-4362 Esch-sur-Alzette, Luxembourg

[3]Department of Internal Medicine and Psychiatry, Saarland University Medical Center, D- 66421 Homburg Saar, Germany

[4]Institute for Social Medicine, Epidemiology and Health Economics, Charité – Universitätsmedizin Berlin, corporate member of Freie Universität Berlin, Humboldt-Universität zu Berlin, and Berlin Institute of Health

[5]Department of Internal and Integrative Medicine, Immanuel Hospital Berlin, Germany

[6]Paracelsus-Elena-Klinik, Kassel, Germany

[7]University Medical Center Göttingen, Germany

[8]Department of Pediatrics, Division of Oncology and Hematology, Charité – Universitätsmedizin Berlin, Corporate Member of Freie Universität Berlin, Humboldt-Universität zu Berlin and Berlin Institute of Health, Berlin, Germany

[9]Digital Health - Connected Healthcare, Hasso Plattner Institute, University of Potsdam, Potsdam, Germany

[10]Department of Dermatology, Venereology and Allergology, Charité—Universitätsmedizin Berlin, Corporate Member of Freie Universität Berlin and Humboldt-Universität zu Berlin, Berlin, Germany

[+]contributed equally

*Correspondence to:

Jochen Schneider (jochen.schneider@uni.lu)
Paul Wilmes (paul.wilmes@uni.lu)

Luxembourg Centre for Systems Biomedicine,
University of Luxembourg, Campus Belval,
7, avenue des Hauts-Fourneaux,
L-4362 Esch-sur-Alzette, Luxembourg

Word count: 3646

For peer review only

Abstract

Introduction

Chronic inflammatory diseases like rheumatoid arthritis (RA) and neurodegenerative disorders like Parkinson's disease (PD) have recently been associated with a decreased diversity in the gut microbiome, emerging as key driver of various diseases. The specific interactions between gut-borne microorganisms and host pathophysiology remain largely unclear. The microbiome can be modulated by interventions comprising nutrition.

The aim of our clinical study is to (1) examine effects of prolonged fasting and time-restricted eating (TRE) on the outcome parameters and the immunophenotypes of RA and PD with (2) special consideration of microbial taxa and molecules associated with changes expected in (1) and (3) identify factors impacting the disease course and treatment by in depth screening of microorganisms and molecules in personalised HuMiX gut-on-chip models, to find novel targets for anti-inflammatory therapy.

Methods and Analysis

This trial is an open-label, multicentre, controlled clinical trial consisting of a cross-sectional and a longitudinal study. A total of 180 patients is recruited. For the cross-sectional study, 60 patients with PD, 60 patients with RA and 60 healthy controls are recruited at two different, specialized clinical sites. For the longitudinal part, 30 patients with PD and 30 patients with RA undergo 5-7 days of prolonged fasting (PF) followed by TRE (16:8) for a period of 12 months. One baseline visit takes place before the PF intervention and 10 follow-up visits will follow over a period of 12 months (April 2021 to November 2023).

Ethics and dissemination

Ethical approval was obtained to plan and conduct the trial from the institutional review board of the Charité-Universitätsmedizin Berlin (EA1/204/19), the ethics committee of the state medical association (Landesärztekammer) of Hessen (2021-2230-zvBO) and the Ethics Review Panel (ERP) of the University of Luxembourg (ERP 21-001-A ExpoBiome). The results of this study will be disseminated through peer-reviewed publications and scientific presentations.

Trial registration number at clinicaltrials.gov:

NCT04847011

Key words: Microbiome, fasting therapy, intermittent fasting, time restricted eating, chronic disease, rheumatoid arthritis, Parkinson's disease, nutrition, chronic diseases, ExpoBiome, inflammation, gut on a chip, HuMiX, immunophenotype, metagenomics, metatranscriptomics, metaproteomics, metabolomics

Strengths and limitations of the study

- The participants of the longitudinal study will be closely monitored for 12 months and routine blood parameters as well as anthropometric data and questionnaires will be precisely documented.

For peer review only

- This study will identify novel microbiome-derived common and disease-associated molecules involved in immune system modulation in two major chronic diseases: RA and PD.
- This study aims at also identifying novel targeted pathways to control chronic inflammatory conditions in the future.
- A limitation is the heterogeneity of the cohorts regarding age and sex, which is due to the prevalence of the diseases: RA is more common in women, while PD is more common in men and has a later disease onset.
- A bias exists in choosing RA and PD as chronic disorders to study immunophenotypes although generalisable results are targeted.

Introduction (1339)

The human microbiome is emerging as a key driver of various diseases through its complex of distinct yet connected biomolecules (referred to as the "*expobiome*")[1, 2]. The expobiome comprises a diverse set of nucleic acids, polypeptides and metabolites which, in the gut alone, are present in substantial concentrations[1]. However, the specific interactions between gut-borne microorganisms and host (patho)physiology remain largely unknown. Although host genetics shape the composition of the gut microbiome, the latter is particularly influenced by non-genetic factors such as lifestyle and diet[3, 4]. Therefore, the microbiome is a plausible target to modify health outcomes.

Individuals suffering from chronic diseases, including autoimmune, metabolic, and neurodegenerative diseases as well as cancer, often present alterations in their gut microbiome composition. These shifts are typically characterised by an overgrowth of one or several microbial species with likely adverse effects as well as a decrease in beneficial taxa[5]. Such imbalances are referred to as dysbiosis. Although structural microbiome changes are clearly detectable, the mechanistic or functional consequences of dysbiosis are still largely unknown. However, they may result in dysregulated interactions with the immune system[6]. Considering the intricacy of the immune system, the question arises whether the observed microbiome changes are cause or consequence of disease. This implies that, in addition to the genetic predisposition of the host, the gut microbiome needs to be considered a potential pathogenic factor or major driver of disease onset and course[3, 4].

RA and PD are two specific examples representing dysregulated microbiome-immune system interactions [7, 8]. RA is a multifactorial, chronic and systemic autoimmune disease, primarily affecting the lining of the synovial joints with a higher risk and younger age for disease onset in women and a global prevalence of 1%[9, 10]. The exact disease pathogenesis is still unclear and no cure for RA currently exists. In addition to the common local articular symptoms of RA, systemic comorbidities can affect the vasculature, metabolism and bones[11]. Besides various environmental risk factors e.g. smoking and a Western diet, the host microbiome is associated with the pathophysiology of the disease[12]. The diversity of the gut microbiome has been reported to be decreased in individuals with RA, compared with the general population, and is correlated with disease duration, activity and autoantibody levels[13, 14]. Studies in murine models also report that autoimmune arthritis is strongly attenuated under germ-free conditions[15]. The introduction of specific bacteria, e.g. segmented filamentous bacteria, into germ-free animals or oral infection with *Porphyromonas gingivalis* drive autoimmune arthritis through activation of T helper cells[15]. Several different taxa, including *Prevotella copri*, *Lactobacillus* spp. and *Colinsella* spp. are enriched in the gut microbiome of patients with RA and correlate positively with disease markers e.g. immunoglobulins IgA and IgG , while other taxa like *Haemophilus* spp. and *Faecalibacterium* spp. are typically found at lower abundances in

For peer review only

patients with RA compared to healthy individuals[13, 16, 17]. Alterations of the gut microbiome may therefore have an important impact on RA pathophysiology[12].

PD affects 0.4-2% of the population over 65 years worldwide and is the second most common progressive neurodegenerative disease with men being 1.5 times more likely to be affected than women[18]. Cardinal symptoms are motor deficiencies such as tremor and rigidity, but also include a wide range of non-motor symptoms, such as hyposmia, depression, insomnia or cognitive impairment, severely impacting patients' quality of life[19]. Aggregations of the protein α-synuclein in the dopaminergic substantia nigra represent the main neuropathological manifestations[20]. PD-associated loss of dopaminergic neurons involves mechanisms of inflammatory and autoimmune responses with microglial activity as a major driver [21].Dysbiosis of the gut microbiome has been associated with the characteristic motor deficits and pathophysiological changes in the enteric and central nervous systems in animal studies. Increased relative abundances of the genera *Akkermansia, Bifidobacterium, Lactobacillus,* and *Methanobrevibacter* and decreased abundances in *Faecalibacterium* and *Roseburia* have been reported[22, 23]. Two recently published clinical-trials with prebiotic supplementation in PD observed a shift in gut microbiome composition, an increase in short-chain fatty acids (SCFA) and a reduction in non-motor-symptoms [24, 25]. Most patients with PD suffer from gastrointestinal symptoms such as constipation and irritable bowel syndrome (IBS) -like symptoms[26]. The gut-brain axis, e.g. by-products produced by the gut microbiome, may contribute to the production of α-synuclein aggregates in the enteric nervous system[27]. In addition, increased intestinal permeability[28] as driver for enteric inflammation occur in PD and substantiate a role of peripheral inflammation in the initiation and the progression of the disease[29].

One factor with known major impact on the gut microbiome and on chronic diseases is diet[7]. Dietary approaches as fasting have already been used by Hippocrates in the the 5th century BCE and have been applied ever since by numerous medical schools to treat acute and chronic diseases [30-32]. Various practices of caloric restriction through fasting have repeatedly shown remarkable health benefits[33, 34]. Maifeld *et al.* found that a 5-day fast followed by a modified Dietary Approach to Stop Hypertension (DASH), with additional emphasis on plant-based and Mediterranean diets, reduced systolic blood pressure, BMI, and the need for antihypertensive medications at three months post intervention compared with DASH alone [35].

Furthermore, Choi *et al.* demonstrated that cycles of a fasting-mimicking diet suppress autoimmunity and stimulate remyelination via oligodendrocyte regeneration in a murine experimental autoimmune encephalomyelitis (EAE) model[36]. Jordan *et al.* described a reduction in monocyte metabolic and inflammatory activity after a short-term fast and conclude that fasting attenuates chronic inflammatory diseases without compromising monocyte capacity for mobilisation during acute infectious inflammation and tissue repair[37].

These improvements can, however, typically only be maintained for a limited period of time, and the symptoms can reappear after reintroduction of the patients' standard diet. Hence, protocols to sustain these beneficial effects are of utmost importance. In mouse models of PD, intermittent fasting (IF) has led to several improvements including decreased excitotoxicity, reduced neurodegeneration and protection against autonomic dysfunction, motor and cognitive decline[38].

IF and PF may have potent immunomodulatory effects which may partially be mediated by the gut microbiome and the fasting induced alterations of the latter[39]. These microbial shifts include upregulation of *Akkermansia muciniphila*, *Bacteroides fragilis*, other *Bacteroides* spp., Proteobacteria, and butyric acid producing *Lachnospiraceae,* but also *Odoribacter*, which is negatively associated with blood pressure[35, 40]. Interestingly, an overall decrease of the Firmicutes/Bacteroidetes ratio could be observed, a high ratio is commonly associated with several pathologies, including RA [41].

For peer review only

A potential mechanism underlying the observed beneficial effects induced by dietary interventions might be a direct gut microbiome-immune system interaction by pattern recognition. The microbiome can regulate the intestinal innate immune system by modulating toll-like receptor (TLR) expression on immunosensor cell surface through microbe-associated molecular patterns (MAMPs), which can consequently trigger cytokine production and up-regulation of molecules on antigen presenting cells, leading to activation of T cells[42]. Therefore, a change in gut microbiome composition can lead to different outcomes in immune signalling pathways and either favour or suppress inflammation and autoimmunity.

The impact and importance of the gut microbiome on human physiology and its potential modifications by nutrition and dietary patterns, have been underestimated for centuries[43]. Reasons may include missing standardised therapeutic protocols, the interindividual variability in the response to fasting, lack of knowledge about possible adverse effects, and difficulties in the interpretation of underlying mechanisms seen in clinical trials, but also in the comparably low potential for achieving economic revenue or scientific impact[8].

Modern experimental approaches and computational integration allow a multi-layer analysis of digestive processes in low caloric settings including the gut microbiome[44]. These technological developments also permit a closer investigation of the link between the immune system and severe caloric restriction.

To our knowledge no clinical trials have been investigating the connection between IF or PF and PD in humans so far[38]. Our study aims to elucidate the causal relationship between the gut microbiome and the immune system. To do so, we will use analyses of the molecular basis of human-microbiome interactions enabled by high throughput methodologies such as the combination of metagenomics, metatranscriptomics and metaproteomics. Moreover, we are aiming at identifying new genes, proteins, metabolites, and host pathways facilitating the development of novel diagnostic and therapeutic tools[45, 46].

Methods and Analysis (2317)

Study objectives

The first objective of the study is to define specific gut microbiome-derived molecules in RA and PD, compared to healthy individuals, and relate this information to the immunophenotypes of the individuals. The second objective is to identify and track common and disease-specific molecular signatures to predict the outcome of a gut microbiome-targeted therapeutic intervention, here fasting, on inflammation-driven symptoms in RA and PD. The third objective of the study is to identify and validate microbiome-derived effector molecules which downregulate pro-inflammatory innate and adaptive immune pathways.

Study design

The ExpoBiome cohort consists of 180 adult individuals, meeting the exclusion and inclusion criteria (Table 1), for the cross-sectional study (objectives 1 and 3) and 60 adult individuals for the longitudinal study (objectives 2 and 3). There are five different arms in total: (1) RA – cross-sectional arm [60 patients], (2) PD – cross-sectional arm [60 patients], and (3) healthy controls – cross-sectional arm [60 patients], (4) RA – longitudinal arm [30 patients], (5) PD – longitudinal arm [30 patients] (Figure1).

For peer review only

At the first visit (T0), patients answer several questionnaires, and blood, urine, saliva and stool samples are obtained (Table 2).
The longitudinal arms (4) and (5) undergo a 5–7-day PF with a dietary energy supply of max. 350-400 kcal per day with vegetable or grain broths as well as fresh vegetable juices[31, 40]. After the PF, the longitudinal arms follow a dietary regimen including the concept of TRE for a period of 12 months following the 16:8 pattern[47]. This means that food intake is allowed ad libitum for 8 h, followed by 16 h of fasting where no food should be consumed. The intake of non-caloric beverages, e.g. water, unsweetened tea or coffee is, however, allowed. The participants attend one follow-up visit (T2) during the PF and 9 follow-up visits during the 12 months of TRE (Figure 1).

Patient and Public Involvement

Feedback of patients during former clinical trials at the study centre in Berlin was integrated in the planning and design of the fasting intervention of this study. Patients are not involved in the conduct, reporting, or dissemination plans of this research.

Recruitment and randomisation

Patients are recruited by the specialised sites via different sources, e.g. by direct referral from either a physician at the Immanuel Hospital Berlin and the outpatient department of the Institute of Social Medicine, Epidemiology and Health Economics at Charité-Universitätsmedizin Berlin, or the Paracelsus-Elena Clinic in Kassel, or by non-personal advertising strategies (e.g. flyers or social media). Participants meeting all the inclusion and no exclusion criteria (Table 1) are assigned to their respective groups (RA, PD, or healthy control) (Figure 1) for the cross-sectional study after written informed consent. Half of the patients from the RA group and half of the patients from the PD group is selected to take part in the longitudinal part of the study, including the fasting intervention according to their availability for all 11 visits and their willingness to follow TRE over 12 months. This study is an open-label trial, as blinding is not feasible in fasting interventions.

Table 1: Inclusion and exclusion criteria

Inclusion criteria	Exclusion criteria
• Age 18-79<br>• One of the following diagnoses: rheumatoid arthritis (first diagnosis >6 weeks ago), Parkinson's disease OR healthy volunteer<br>• Control ("healthy") individuals must be without any evidence of active known or treated RA, without any evidence of active, known or treated central nervous system disease, and without a known family history of idiopathic PD<br>• Control individuals should match the RA or PD individuals as closely as possible (sex, age, education)<br>• Present written declaration of consent	• Gout or proven bacterial arthritis<br>• Participation in another study<br>• Existing/current eating disorder (bulimia nervosa, anorexia nervosa) within the past 5 years<br>• Severe internal disease (e.g. kidney deficiency with creatinine > 2mg/dl)<br>• Existing vegan diet or fasting during the last six months<br>• Presence or suspicion of atypical PD (e.g. early dementia, early autonomous dysfunction)<br>• Diagnosis of chronic inflammatory bowel diseases, celiac disease or colorectal cancer according to the guidelines of the German Society of Gastroenterology<br>• Use of anti-psychotic drugs

For peer review only

• Ability to understand the patient information and willingness to sign the consent form<br>• Consent to specimen collection and specimen use	• Antibiotic use during the previous 12 months<br>• Start of novel therapy with disease-modifying anti-rheumatic drugs<br>• Pregnancy or breastfeeding women<br>• Contraindication for additional blood draws (e.g. haemoglobin <10)<br>• BMI < 18.5<br>• Psychiatric illness that limits understanding of the examination protocol (unable to consent)

Fasting dietary counselling

The fasting group is closely monitored by nutritionists trained in fasting therapy, backed up by physicians experienced in fasting, from the Charité – Universitätsmedizin Berlin and the Paracelsus-Elena Clinic to ensure a uniform implementation of the fasting guidelines and the well-being of the study participants. The monitoring consists of several in person and virtual meetings which held individually or in group settings. Five meetings including the visits T0 and T2 during the fasting week as well as a group meeting after PF to ensure a well-managed start to the TRE phase take place. Group sessions are standardised using a pre-set deck of slides to be discussed during the group meetings with only minor disease-related differences between the PD and RA groups. All longitudinal participants receive a study-specific script with information on fasting procedures.

Medication

The medical treatments of the patients are monitored and documented with every clinical visit. The fasting intervention might necessitate temporary adjustments of several medications e.g. anti-diabetic and anti-hypertensive drugs as insulin levels and hypertension will be reduced due to lack of food intake [31].

Data collection

Sample and data collection is performed at the two clinical sites, Charité – Universitätsmedizin Berlin and Paracelsus-Elena Clinic (Table 2).

Table 2: Sampling procedures.

a) Biochemical samples and procedures

Blood (123 mL at T0, 23 mL at T2-T11)
Stool collection (2 mL at T0 and T3-T11)
Saliva collection (3.5 mL at T0-T11)
Midstream urine (50 mL at T0 -T11)

b) Questionnaires

Disease specific

For peer review only

PD:<br>• Disease Activity Score (DAS-28) [48]<br>• Parkinson's Disease Sleep Scale-2 (PDSS-2) [49]<br>• Parkinson's Disease Questionnaire-39 (PDQ-39)[50]<br>• Simplified Disease Index Score (SDAI) [51]<br>• Funktionsfragebogen Hannover (FFbH-R) [52]<br>• Movement Disorder Society Unified PD Rating Scale (MDS-UPDRS)[53]<br>• Non-Motor Symptoms Questionnaire (NMSQ)[54]<br>• Non-Motor Symptoms Scale (NMSS)[55]<br>RA:<br>• Disease Activity Score (DAS-28) [51]<br>• Non-Motor Symptoms Questionnaire (NMSQ) [54]<br>• Funktionsfragebogen Hannover (FFbH-R) [52]
Dietary behaviour and lifestyle
• Fasting experience, expectation, and intervention<br>• Lifestyle<br>• 24H-Food-recall<br>• Food Frequency Questionnaire (FFQ)
General health and well-being
• Health Assessment Questionnaire (HAQ)[56]<br>• Bristol Stool Scale[57]<br>• Quality of Life questionnaire (WHO-5)[58]<br>• Hospital Anxiety and Depression Scale (HADS)[59]

For peer review only

• Profile of Mood States[60]

Anthropometric data and questionnaires

The electronic data capture system REDCap[61], a secure web-based application, is used to record all individual specific data. All data is stored on a secure server infrastructure at the host institution in Luxembourg. Weight, height, body mass index (BMI), heart rate and blood pressure in sitting and standing position as well as waist-hip-ratio is determined at every visit. Dietary behaviour, sociodemographic measurements (age, sex, education level, employment status, marital status), family history, current and previous illness and co-morbidities, and current medications, as well as disease-specific data, questionnaires about the well-being of the patients and data on the behavioural factors are collected at baseline, T6 (week 3), T9 (month 6) and T11 (month 12) (Table 2). Questionnaires (24h-Food Recall, Bristol Stool Scale) are answered at all visits by the study participants. Data storage, analysis and exchange are done only in pseudonymised fashion. The nutritional data is analysed using the Nutrilog 3.20 software (Nutrilog SAS, Marans).

Blood samples and parameters

Blood samples are collected at each visit, and immediately used for peripheral blood mononuclear cell (PBMC) isolation (T0), analysis by the study laboratory and centrifugation to freeze plasma samples at -80°C (T0-T11). A clinical standard laboratory report is generated after every visit for each study participant (Table 3). In addition to routine blood parameters, anti-citrullinated protein antibody (ACPA), zonulin, fatty-acid binding protein 2 (FABP2), and calprotectin levels are measured. Aliquots are securely stored to account for novel observations and testing of hypotheses.

Table 3: Routine blood parameters measured at each timepoint (T0 for cross-sectional study, T0-T11 for longitudinal study)

Haematology – EDTA-blood	Clinical Chemistry – Serum
Basophils, %	Albumin
Basophils, abs.	ALT, 37°C
Eosinophils, %	Alkaline Phosphatase, 37°C
Eosinophils, abs.	AST, 37°C
Erythrocytes	Bilirubin, total
Haematocrit	Cholinesterase
Haemoglobin	Cholesterol
HbA1c	Creatinine
Leucocytes	hs-CRP
Lymphocytes, %	Glucose, serum
Lymphocytes, abs.	Gamma-GT, 37°C
MCH	HDL-Cholesterol
MCHC	LDL-Cholesterol

For peer review only

MCV	Potassium
Monocytes, %	Sodium
Monocytes, abs.	Total Protein
Neutrophils, %	Triglycerides
Neutrophils, abs.	Uric Acid
Platelets	Urea/BUN
RDW	**Proteins – Serum**
Reticulocytes	Rheumatoid factor H 35.9
Reticulocytes	**Hormones – Serum**
Reticulocytes, abs.	Insulin
	TSH (basal)

Stool, urine and saliva samples

The samples listed in Table 2 are collected at each visit, except for stool samples on T2 (fasting week) and immediately frozen and stored at -80°C. Stool characteristics are recorded at the time of the sampling. Faecal samples represent the main sample type for resolving the dynamic processes driven by microbiome in the gut. Also, as the gut microbiome is prone to diurnal fluctuations, the stool samples are collected in the morning, as far as possible.

Methods applied to samples

Biomolecular extractions

The collected stool samples undergo a biomolecular extraction procedure to allow isolation of concomitant DNA, RNA, proteins, peptides and metabolites from single, unique faecal water samples; this process involves cryo-milling the samples in liquid nitrogen, disassociating metabolites from membrane and cell wall components in a solvent mixture of methanol, chloroform and water and lastly proteins and RNA extraction by a methanol/chloroform and phenol buffer [62, 63]. Faecal water is recovered following centrifugation and filtration, at low-speed or low-flow, respectively, to avoid cell lysis. Nucleic acids are preserved by the addition of ribonuclease inhibitors and isolated by silica-column-based techniques. This protocol involves the use of a robotic platform, ensuring a higher level of standardisation and reproducibility[2].

Coupled metagenomic and metatranscriptomic analyses

Prior to sequencing library preparation, internal standards are introduced to obtain quantitative sequencing data[64]. Contamination-free metagenomic (MG) and metatranscriptomic (MT) data is generated, processed and analysed using the Integrated Meta-omics Pipeline (IMP)[45], which incorporates pre-processing, assembly, gene annotation, mapping of reads, single nucleotide polymorphism calling, data normalisation as well as analyses of community structure and function in a fully reproducible software framework based on Docker. The MG and MT data is specifically screened for enrichments in genes and pathways with known immunogenic properties[65]. The extracellular biomolecules are linked to specific microbial populations based on the intracellular metagenomic data [66]. In addition, the sequencing data is mapped against genomes of food components[44]. The quantitative data is also related to microbial population sizes to determine the

For peer review only

contribution of the resolved microbial populations in stool to the extracellular DNA and RNA complements[67].

Metaproteomics

For the metaproteomic analyses, filtration is used to separate extracellular peptides from the obtained (poly)peptides. The resulting smaller fractions are then desalted and analysed without proteolytic digestion via liquid chromatography (LC) and mass spectrometry (MS) on an EasyNano-LC coupled online to a QExactive-Plus mass spectrometer (ThermoScientific, Waltham, USA). The identification of ribosomal peptides is done with an integrated catalogue of MG and MT data, while the non-ribosomal peptides are identified using different tools, i.e., MyriMatch, DirecTag as well as CycloBranch[45, 68, 69]. The metaproteomic data also allows identification of extracellular (poly)peptides with possible pathogenic functions including protein misfolding and molecular mimicry[70, 71].

Metabolomics

Metabolomic data is analysed using a combination of targeted and untargeted approaches [44, 63, 72]. This highlights the major metabolite classes produced by the gut microbiome with an effect on human physiology including organic acids, SCFA, lipids, branched-chain fatty acids, branched-chain amino acids, vitamins, bile acids and neurotransmitters. Besides external compound calibration series for quantification and quality control samples to ensure data normalisation and data acquisition quality assessment, the metabolite extraction fluid is fortified with multiple internal standards to improve method precision and accuracy[73, 74]. The data is compared to in-house databases and public mass spectral libraries to identify known metabolites. The metabolomic data complements the metagenomic and metatranscriptomic data and thus allows further establishments of conclusive links to metabolic properties in the gut.

Deep immune profiling

Deep immune profiling is done using a recently established and optimised panel of metal-labelled antibodies together with cytometry coupled to mass spectrometry (MS), the Maxpar Direct Immune Profiling System (MDIPA). This approach allows the simultaneous quantification of 38 parameters on single cells. Whole blood is stained with the MDIPA kit and stabilised with Proteomic stabiliser Prot-1 (501351694, Smart Tube Inc., Las Vegas) before storage at -80°C. The quantified immune cells included in the MDIPA panel are CD3+, CD4+, CD8+, monocytes, dendritic cells, granulocytes, MAIT, T cells, NK and B cells[75]. Cytokine expression profiles is analysed on blood plasma using the Human Luminex performance Cytokine Panel (R&D Systems Europe, Abingdon), measuring CCL3, CCL4, CCL5, GM-CSF, IL-1β, IL-2, IL-4, IL-5, IL-6, IL-8, IL-10, IL-12p70, IL-13, IL-15, IL-18, IL-21, IL-27, IL-33, IFN-β, Galectin-1, IFN-γ and TNF-α [65].

Gut-on-a-chip models

PBMCs isolated from T0 blood samples are co-cultured with gut-derived microbes under physiologically representative conditions using the gut-on-a-chip model HuMiX[76]. This model of the human gastrointestinal interface allows the investigation of the interactions between immune, epithelial and bacterial cells and specifically the response to fasting in personalised in vitro models.

The Expobiome Map

The Expobiome Map (https://expobiome.lcsb.uni.lu) illustrates the diverse complex of microbial immunogenic molecules, including nucleic acids, (poly)peptides, structural molecules, and metabolites. The interactions between this "expobiome" and human immune pathways are encoded

For peer review only

in the context of chronic diseases[1]. The ExpoBiome Map is visualised using the MINERVA Platform[77]. Clicking on different elements on the map reveals factors they affect and are affected by, allowing an easier navigation through the complex relationships between individual microbiome components in relation to human disease. The multi-omics data generated in the present study will be integrated with the Map.

Exploratory analysis of novel host-microbiome interactions

Unknown non-ribosomal peptides or metabolite features are associated through correlation with transcripts, proteins and metabolites. Extracellular DNA fragments, as well as transcripts, proteins and ribosomal peptides are linked to their genomic context by using IMP[45]. The data generated by the project will be connected and collated to existing, publicly available datasets.

Outcome parameters

Primary Outcome

The primary endpoint of the study is the characterisation of the gut microbiome. The evaluation includes both between-group and within-group differences in the longitudinal study arms with the fasting intervention.

Secondary Outcome Measures

Secondary outcomes include the identification of common and disease-specific molecular signatures and the characterisation of microbiome-derived effector molecules impacting the innate and adaptive immune pathways. Furthermore, several additional parameters mentioned in *Anthropometric data and questionnaires* are assessed over a period of 12 months.

Sample size and power calculation

A power calculation using pilot metatranscriptomic data based on faecal extracellular RNA samples was performed to determine the number of subjects to be recruited for the ExpoBiome project. The obtained relative abundances of genera were used for the calculation of the required sample size per group. The power calculation was based on the algorithm as described by Tusher, Tibshirani, and Chu[78]. To achieve a power of 90% (at $\alpha = 0.05$), a total of 50 individuals per group (RA, PD, healthy controls) must be analysed. Considering any possible dropouts, 20% additional subjects are recruited, resulting in a total number of 180 individuals, i.e., 60 per group.

Adverse events

There are no major risks expected for participants. Minor common adverse effects of PF might include headaches, nausea, insomnia, back pain, dyspepsia and fatigue[79]. Any occurring adverse events are recorded at each visit in REDCap[61]. Serious adverse events are communicated to the study coordinator and principal investigator within 24 h of their report.

Data management, monitoring, analysis and evaluation of data

The study participants receive a study ID (pseudonym) which is used for all collected data. Self-administered questionnaires are directly recorded in REDCap. Participant files are kept for at least 10 years at the respective clinical sites.
Weekly meetings between the study team, the different clinical partners and the principal investigator, ensure a close monitoring of the data. Any occurring adverse events or other issues are thus handled immediately.

For peer review only

Different statistical tests are performed according to the nature of the data. A premature termination of the study is not envisaged; therefore, no interim analysis is done. Different correlation measures are applied, including Spearman correlation, mutual information on discretised data, distance correlation, maximum information criterion, local similarity analysis and the bioenv approach. Comparison across all omic levels allows identification of common and disease-specific signatures. Multivariate machine learning is used to link different data features to observed patterns.
The longitudinal part of the study continues for a period of 12 months. After finalisation of this period, there is no follow-up of the participants. Interesting findings will be further validated using the existing sample set and analyses may be performed on additionally collected samples.
The SPIRIT - checklist (Standard Protocol Items: Recommendations for Interventional Trials) was used to write this protocol [80].

Trial status

The recruitment for the ExpoBiome study started in April 2021 and is currently ongoing. All study participants should be recruited by the end of 2022. The sample collection will take place from April 2021 to November 2023.

Acknowledgements

We thank Dr. Catharina Delebinski, Melanie Dell'Oro, Grit Langhans, Ursula Reuß, Maik Schröder and Nadine Sylvester for their support during the study.

Author contributions:

Study design and protocol were done by Bérénice Hansen, Cédric C. Laczny, Jochen G. Schneider, Paul Wilmes; the interventional concept was drawn by Etienne Hanslian, Daniela Liebscher, Andreas Michalsen, Anika Hartmann, Brit Mollenhauer, Sebastian Schade, Nico Steckhan, Jochen G. Schneider, Paul Wilmes; the clinical trial was designed and is conducted by Etienne Hanslian, Daniela Liebscher, Andreas Michalsen, Anika Hartmann, Brit Mollenhauer, Sebastian Schade; the procured funding was provided by Paul Wilmes; the planning of high-throughput applications, statistical planning, sample size calculation and randomisation were defined by Cédric C. Laczny, Jochen G. Schneider, Paul Wilmes, Kirsten Roomp; the initial draft of the manuscript and coordination of the editing process were performed by Bérénice Hansen; the protocol preparation has been done by Bérénice Hansen, Audrey Frachet-Bour, Janine Habier; the planning of the data analysis was done by Cédric C. Laczny, Jochen G. Schneider, Paul Wilmes, Kirsten Roomp, Velma T.E. Aho, Marek Ostaszewski; all authors contributed equally with edits, comments and feedback, read and approved the final manuscript.

Funding statement: This project has received funding from the European Research Council (ERC) under the European Union's Horizon 2020 research and innovation program (grant agreement No. 863664).

Competing interests statement

None declared.

For peer review only

Supplements

The SPIRIT checklist was used to write our report[80].

References

1. Wilmes, P., et al., *The gut microbiome molecular complex in human health and disease.* Cell Host Microbe, 2022. **30**(9): p. 1201-1206.
2. De Saedeleer, B., et al., *Systematic characterization of human gut microbiome-secreted molecules by integrated multi-omics.* ISME Communications, 2021. **1**(1): p. 82.
3. Greenhalgh, K., et al., *The human gut microbiome in health: establishment and resilience of microbiota over a lifetime.* Environ Microbiol, 2016. **18**(7): p. 2103-16.
4. Hall, A.B., A.C. Tolonen, and R.J. Xavier, *Human genetic variation and the gut microbiome in disease.* Nature Reviews Genetics, 2017. **18**(11): p. 690-699.
5. Baldini, F., et al., *Parkinson's disease-associated alterations of the gut microbiome predict disease-relevant changes in metabolic functions.* BMC Biol, 2020. **18**(1): p. 62.
6. Yoo, J.Y., et al., *Gut Microbiota and Immune System Interactions.* Microorganisms, 2020. **8**(10).
7. Sonnenburg, J.L. and F. Bäckhed, *Diet–microbiota interactions as moderators of human metabolism.* Nature, 2016. **535**(7610): p. 56-64.
8. Zmora, N., J. Suez, and E. Elinav, *You are what you eat: diet, health and the gut microbiota.* Nat Rev Gastroenterol Hepatol, 2019. **16**(1): p. 35-56.
9. Guo, Q., et al., *Rheumatoid arthritis: pathological mechanisms and modern pharmacologic therapies.* Bone Res, 2018. **6**: p. 15.
10. Healthline, V.L. *Rheumatoid Arthritis by the Numbers: Facts, Statistics, and You*. 2021.
11. Scherer, H.U., T. Häupl, and G.R. Burmester, *The etiology of rheumatoid arthritis.* Journal of Autoimmunity, 2020. **110**: p. 102400.
12. Bodkhe, R., B. Balakrishnan, and V. Taneja, *The role of microbiome in rheumatoid arthritis treatment.* Ther Adv Musculoskelet Dis, 2019. **11**: p. 1759720x19844632.
13. Chen, J., et al., *An expansion of rare lineage intestinal microbes characterizes rheumatoid arthritis.* Genome Medicine, 2016. **8**(1): p. 43.
14. Kitamura, K., et al., *Oral and Intestinal Bacterial Substances Associated with Disease Activities in Patients with Rheumatoid Arthritis: A Cross-Sectional Clinical Study.* J Immunol Res, 2022. **2022**: p. 6839356.
15. Wu, H.J., et al., *Gut-residing segmented filamentous bacteria drive autoimmune arthritis via T helper 17 cells.* Immunity, 2010. **32**(6): p. 815-27.
16. Scher, J.U., et al., *Expansion of intestinal Prevotella copri correlates with enhanced susceptibility to arthritis.* eLife, 2013. **2**: p. e01202.
17. Zhang, X., et al., *The oral and gut microbiomes are perturbed in rheumatoid arthritis and partly normalized after treatment.* Nature Medicine, 2015. **21**(8): p. 895-905.
18. Lubomski, M., et al., *Parkinson's disease and the gastrointestinal microbiome.* J Neurol, 2020. **267**(9): p. 2507-2523.
19. Opara, J., et al., *Motor assessment in Parkinson`s disease.* Ann Agric Environ Med, 2017. **24**(3): p. 411-415.

For peer review only

20. Tysnes, O.B. and A. Storstein, *Epidemiology of Parkinson's disease.* J Neural Transm (Vienna), 2017. **124**(8): p. 901-905.
21. Garcia, P., et al., *Neurodegeneration and neuroinflammation are linked, but independent of alpha-synuclein inclusions, in a seeding/spreading mouse model of Parkinson's disease.* Glia, 2022. **70**(5): p. 935-960.
22. Heintz-Buschart, A. and P. Wilmes, *Human Gut Microbiome: Function Matters.* Trends Microbiol, 2018. **26**(7): p. 563-574.
23. Romano, S., et al., *Meta-analysis of the Parkinson's disease gut microbiome suggests alterations linked to intestinal inflammation.* npj Parkinson's Disease, 2021. **7**(1): p. 27.
24. Becker, A., et al., *Effects of Resistant Starch on Symptoms, Fecal Markers, and Gut Microbiota in Parkinson's Disease - The RESISTA-PD Trial.* Genomics Proteomics Bioinformatics, 2022. **20**(2): p. 274-287.
25. Hall, D.A., et al., *An open label, non-randomized study assessing a prebiotic fiber intervention in a small cohort of Parkinson's disease participants.* Nat Commun, 2023. **14**(1): p. 926.
26. Mertsalmi, T.H., et al., *More than constipation - bowel symptoms in Parkinson's disease and their connection to gut microbiota.* European journal of neurology, 2017. **24**(11): p. 1375-1383.
27. Dogra, N., R.J. Mani, and D.P. Katare, *The Gut-Brain Axis: Two Ways Signaling in Parkinson's Disease.* Cell Mol Neurobiol, 2022. **42**(2): p. 315-332.
28. Forsyth, C.B., et al., *Increased intestinal permeability correlates with sigmoid mucosa alpha-synuclein staining and endotoxin exposure markers in early Parkinson's disease.* PLoS One, 2011. **6**(12): p. e28032.
29. Devos, D., et al., *Colonic inflammation in Parkinson's disease.* Neurobiol Dis, 2013. **50**: p. 42-8.
30. Britannica, *The Editors of Encyclopaedia. "fasting".* Encyclopedia Britannica,, Accessed 3 October 2022. https://www.britannica.com/topic/fasting.
31. Hartmann, A.M., et al., *Efficacy of therapeutic fasting and plant-based diet in patients with rheumatoid arthritis (NutriFast): study protocol for a randomised controlled clinical trial.* BMJ Open, 2021. **11**(8): p. e047758.
32. Michalsen, A., *Prolonged fasting as a method of mood enhancement in chronic pain syndromes: a review of clinical evidence and mechanisms.* Curr Pain Headache Rep, 2010. **14**(2): p. 80-7.
33. Vargas-Molina, S., et al., *Effects of a low-carbohydrate ketogenic diet on health parameters in resistance-trained women.* Eur J Appl Physiol, 2021. **121**(8): p. 2349-2359.
34. Mattison, J.A., et al., *Caloric restriction improves health and survival of rhesus monkeys.* Nature Communications, 2017. **8**(1): p. 14063.
35. Maifeld, A., et al., *Fasting alters the gut microbiome reducing blood pressure and body weight in metabolic syndrome patients.* Nature Communications, 2021. **12**(1): p. 1970.
36. Choi, I.Y., et al., *A Diet Mimicking Fasting Promotes Regeneration and Reduces Autoimmunity and Multiple Sclerosis Symptoms.* Cell Rep, 2016. **15**(10): p. 2136-2146.
37. Jordan, S., et al., *Dietary Intake Regulates the Circulating Inflammatory Monocyte Pool.* Cell, 2019. **178**(5): p. 1102-1114.e17.

For peer review only

38. Neth, B.J., et al., *The Role of Intermittent Fasting in Parkinson's Disease.* Front Neurol, 2021. **12**: p. 682184.
39. Cignarella, F., et al., *Intermittent Fasting Confers Protection in CNS Autoimmunity by Altering the Gut Microbiota.* Cell Metab, 2018. **27**(6): p. 1222-1235.e6.
40. Mesnage, R., et al., *Changes in human gut microbiota composition are linked to the energy metabolic switch during 10 d of Buchinger fasting.* J Nutr Sci, 2019. **8**: p. e36.
41. Magne, F., et al., *The Firmicutes/Bacteroidetes Ratio: A Relevant Marker of Gut Dysbiosis in Obese Patients?* Nutrients, 2020. **12**(5).
42. Purchiaroni, F., et al., *The role of intestinal microbiota and the immune system.* Eur Rev Med Pharmacol Sci, 2013. **17**(3): p. 323-33.
43. Leeming, E.R., et al., *Effect of Diet on the Gut Microbiota: Rethinking Intervention Duration.* Nutrients, 2019. **11**(12): p. 2862.
44. Heintz-Buschart, A., et al., *Integrated multi-omics of the human gut microbiome in a case study of familial type 1 diabetes.* Nature Microbiology, 2016. **2**(1): p. 16180.
45. Narayanasamy, S., et al., *IMP: a pipeline for reproducible reference-independent integrated metagenomic and metatranscriptomic analyses.* Genome Biology, 2016. **17**(1): p. 260.
46. Wilmes, P., A. Heintz-Buschart, and P.L. Bond, *A decade of metaproteomics: where we stand and what the future holds.* Proteomics, 2015. **15**(20): p. 3409-17.
47. Gabel, K., et al., *Effects of 8-hour time restricted feeding on body weight and metabolic disease risk factors in obese adults: A pilot study.* Nutr Healthy Aging, 2018. **4**(4): p. 345-353.
48. Wells G, B.J., Teng J, et al, *Validation of the 28-joint Disease Activity Score (DAS28) and European League Against Rheumatism response criteria based on C-reactive protein against disease progression in patients with rheumatoid arthritis, and comparison with the DAS28 based on erythrocyte sedimentation rate.* Annals of the Rheumatic Diseases 2009. **68**: p. 954-960.
49. Trenkwalder, C., et al., *Parkinson's disease sleep scale--validation of the revised version PDSS-2.* Mov Disord, 2011. **26**(4): p. 644-52.
50. Bushnell, D.M. and M.L. Martin, *Quality of life and Parkinson's disease: translation and validation of the US Parkinson's Disease Questionnaire (PDQ-39).* Qual Life Res, 1999. **8**(4): p. 345-50.
51. Smolen, J.S., et al., *A simplified disease activity index for rheumatoid arthritis for use in clinical practice.* Rheumatology (Oxford), 2003. **42**(2): p. 244-57.
52. Raspe, H.H., Hagedorn, U., Kohlmann, T., & Mattussek, S., *Der Funktionsfragebogen Hannover (FFbH): Ein Instrument zur Funktionsdiagnostik bei polyartikulären Gelenkerkrankungen*, in *Ergebnisse sozialwissenschaftlicher Evaluation eines Modellversuchs (pp. 164-182)*. 1990, Schattauer Verlag.
53. Goetz, C.G., et al., *Movement Disorder Society-sponsored revision of the Unified Parkinson's Disease Rating Scale (MDS-UPDRS): Process, format, and clinimetric testing plan.* Mov Disord, 2007. **22**(1): p. 41-7.
54. Chaudhuri, K.R., et al., *International multicenter pilot study of the first comprehensive self-completed nonmotor symptoms questionnaire for Parkinson's disease: the NMSQuest study.* Mov Disord, 2006. **21**(7): p. 916-23.
55. Chaudhuri, K.R., et al., *The metric properties of a novel non-motor symptoms scale for Parkinson's disease: Results from an international pilot study.* Mov Disord, 2007. **22**(13): p. 1901-11.

For peer review only

56. Wolfe, F., *A brief clinical health assessment instrument clinhaq.* Arthritis and Rheumatism, 1989. **32**(4 Suppl): p. S99-S99.

57. Lewis, S.J. and K.W. Heaton, *Stool form scale as a useful guide to intestinal transit time.* Scand J Gastroenterol, 1997. **32**(9): p. 920-4.

58. Topp, C.W., et al., *The WHO-5 Well-Being Index: a systematic review of the literature.* Psychother Psychosom, 2015. **84**(3): p. 167-76.

59. Zigmond, A.S. and R.P. Snaith, *The hospital anxiety and depression scale.* Acta Psychiatr Scand, 1983. **67**(6): p. 361-70.

60. McNair DM, L.M., Droppleman LF, *Edits Manual for the Profile of Mood States (Poms). Rev ed.* . San Diego: Educational and Industrial Testing Service, 1992.

61. Harris, P.A., et al., *Research electronic data capture (REDCap)--a metadata-driven methodology and workflow process for providing translational research informatics support.* J Biomed Inform, 2009. **42**(2): p. 377-81.

62. Wilmes, P., Roume, H., Hiller, K. & Cordes, T., *Method and kit for the isolation of genomic DNA, RNA, proteins and metabolites from a single biological sample.*, in *World Intellectual Property Organization*, C.D.R.P.-G.L. Université Du Luxembourg, Editor. 2014: Switzerland.

63. Roume, H., et al., *A biomolecular isolation framework for eco-systems biology.* The ISME Journal, 2013. **7**(1): p. 110-121.

64. Locati, M.D., et al., *Improving small RNA-seq by using a synthetic spike-in set for size-range quality control together with a set for data normalization.* Nucleic Acids Res, 2015. **43**(14): p. e89.

65. Wampach, L., et al., *Birth mode is associated with earliest strain-conferred gut microbiome functions and immunostimulatory potential.* Nature Communications, 2018. **9**(1): p. 5091.

66. Albanese, D. and C. Donati, *Strain profiling and epidemiology of bacterial species from metagenomic sequencing.* Nature Communications, 2017. **8**(1): p. 2260.

67. Vandeputte, D., et al., *Quantitative microbiome profiling links gut community variation to microbial load.* Nature, 2017. **551**(7681): p. 507-511.

68. Tang, H., S. Li, and Y. Ye, *A Graph-Centric Approach for Metagenome-Guided Peptide and Protein Identification in Metaproteomics.* PLoS Comput Biol, 2016. **12**(12): p. e1005224.

69. Tabb, D.L., C.G. Fernando, and M.C. Chambers, *MyriMatch: highly accurate tandem mass spectral peptide identification by multivariate hypergeometric analysis.* J Proteome Res, 2007. **6**(2): p. 654-61.

70. Heintz-Buschart, A., et al., *The nasal and gut microbiome in Parkinson's disease and idiopathic rapid eye movement sleep behavior disorder.* Mov Disord, 2018. **33**(1): p. 88-98.

71. Chen, S.G., et al., *Exposure to the Functional Bacterial Amyloid Protein Curli Enhances Alpha-Synuclein Aggregation in Aged Fischer 344 Rats and Caenorhabditis elegans.* Scientific Reports, 2016. **6**(1): p. 34477.

72. Wilmes, P., et al., *Metabolome-proteome differentiation coupled to microbial divergence.* mBio, 2010. **1**(5).

73. Kim, D.H., et al., *LC-MS-based absolute metabolite quantification: application to metabolic flux measurement in trypanosomes.* Metabolomics, 2015. **11**(6): p. 1721-1732.

For peer review only

For peer review only - http://bmjopen.bmj.com/site/about/guidelines.xhtml

74. Lei, Z., D.V. Huhman, and L.W. Sumner, *Mass spectrometry strategies in metabolomics.* J Biol Chem, 2011. **286**(29): p. 25435-42.
75. *Immunophenotyping assessment in a COVID-19 cohort (IMPACC): A prospective longitudinal study.* Sci Immunol, 2021. **6**(62).
76. Shah, P., et al., *A microfluidics-based in vitro model of the gastrointestinal human–microbe interface.* Nature Communications, 2016. **7**(1): p. 11535.
77. Aho, V.T.E., et al., *SnapShot: The Expobiome Map.* Cell Host Microbe, 2022. **30**(9): p. 1340-1340.e1.
78. Tusher, V.G., R. Tibshirani, and G. Chu, *Significance analysis of microarrays applied to the ionizing radiation response.* Proc Natl Acad Sci U S A, 2001. **98**(9): p. 5116-21.
79. Finnell, J.S., et al., *Is fasting safe? A chart review of adverse events during medically supervised, water-only fasting.* BMC Complementary and Alternative Medicine, 2018. **18**(1): p. 67.
80. Chan, A.-W., et al., *SPIRIT 2013 explanation and elaboration: guidance for protocols of clinical trials.* BMJ : British Medical Journal, 2013. **346**: p. e7586.

Figure Legends

Figure 1. Study design. This figure illustrates the study design with five different arms in total, two of which continue with the longitudinal part of the study. Visits take place at the clinical sites at each timepoint and include the collection of the displayed samples. This image was generated using Biorender software (http://www.biorender.com). T, timepoint; W, week; D, day; M, month.

For peer review only

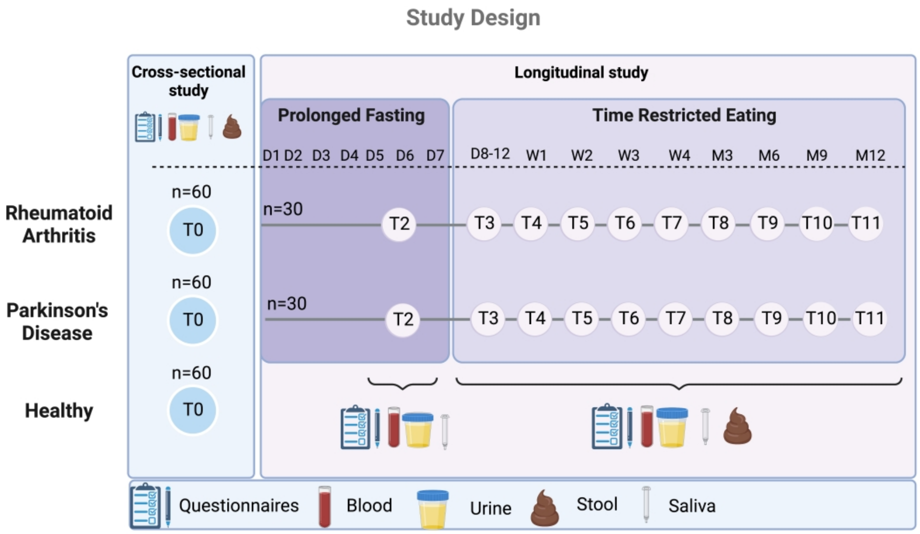

Figure 1. Study design. This figure illustrates the study design with five different arms in total, two of which continue with the longitudinal part of the study. Visits take place at the clinical sites at each timepoint and include the collection of the displayed samples. This image was generated using Biorender software (http://www.biorender.com). T, timepoint; W, week; D, day; M, month.

279x177mm (600 x 600 DPI)

Reporting checklist for protocol of a clinical trial.

Based on the SPIRIT guidelines.

Instructions to authors

Complete this checklist by entering the page numbers from your manuscript where readers will find each of the items listed below.

Your article may not currently address all the items on the checklist. Please modify your text to include the missing information. If you are certain that an item does not apply, please write "n/a" and provide a short explanation.

Upload your completed checklist as an extra file when you submit to a journal.

In your methods section, say that you used the SPIRITreporting guidelines, and cite them as:

Chan A-W, Tetzlaff JM, Gøtzsche PC, Altman DG, Mann H, Berlin J, Dickersin K, Hróbjartsson A, Schulz KF, Parulekar WR, Krleža-Jerić K, Laupacis A, Moher D. SPIRIT 2013 Explanation and Elaboration: Guidance for protocols of clinical trials. BMJ. 2013;346:e7586

		Reporting Item	Page Number
**Administrative information**			
Title	#1	Descriptive title identifying the study design, population, interventions, and, if applicable, trial acronym	1
Trial registration	#2a	Trial identifier and registry name. If not yet registered, name of intended registry	2
Trial registration: data set	#2b	All items from the World Health Organization Trial Registration Data Set	n/a
Protocol version	#3	Date and version identifier	n/a
Funding	#4	Sources and types of financial, material, and other support	12
Roles and responsibilities: contributorship	#5a	Names, affiliations, and roles of protocol contributors	1

For peer review only

Roles and responsibilities: sponsor contact information	#5b	Name and contact information for the trial sponsor	1
Roles and responsibilities: sponsor and funder	#5c	Role of study sponsor and funders, if any, in study design; collection, management, analysis, and interpretation of data; writing of the report; and the decision to submit the report for publication, including whether they will have ultimate authority over any of these activities	1, 12
Roles and responsibilities: committees	#5d	Composition, roles, and responsibilities of the coordinating centre, steering committee, endpoint adjudication committee, data management team, and other individuals or groups overseeing the trial, if applicable (see Item 21a for data monitoring committee)	1, 12
**Introduction**			
Background and rationale	#6a	Description of research question and justification for undertaking the trial, including summary of relevant studies (published and unpublished) examining benefits and harms for each intervention	5
Background and rationale: choice of comparators	#6b	Explanation for choice of comparators	5
Objectives	#7	Specific objectives or hypotheses	5
Trial design	#8	Description of trial design including type of trial (eg, parallel group, crossover, factorial, single group), allocation ratio, and framework (eg, superiority, equivalence, non-inferiority, exploratory)	5
**Methods: Participants, interventions, and outcomes**			
Study setting	#9	Description of study settings (eg, community clinic, academic hospital) and list of countries where data will be collected. Reference to where list of study sites can be obtained	6

For peer review only

Eligibility criteria	#10	Inclusion and exclusion criteria for participants. If applicable, eligibility criteria for study centres and individuals who will perform the interventions (eg, surgeons, psychotherapists)	6
Interventions: description	#11a	Interventions for each group with sufficient detail to allow replication, including how and when they will be administered	5,7
Interventions: modifications	#11b	Criteria for discontinuing or modifying allocated interventions for a given trial participant (eg, drug dose change in response to harms, participant request, or improving / worsening disease)	5,7
Interventions: adherance	#11c	Strategies to improve adherence to intervention protocols, and any procedures for monitoring adherence (eg, drug tablet return; laboratory tests)	7
Interventions: concomitant care	#11d	Relevant concomitant care and interventions that are permitted or prohibited during the trial	6
Outcomes	#12	Primary, secondary, and other outcomes, including the specific measurement variable (eg, systolic blood pressure), analysis metric (eg, change from baseline, final value, time to event), method of aggregation (eg, median, proportion), and time point for each outcome. Explanation of the clinical relevance of chosen efficacy and harm outcomes is strongly recommended	11
Participant timeline	#13	Time schedule of enrolment, interventions (including any run-ins and washouts), assessments, and visits for participants. A schematic diagram is highly recommended (see Figure)	5
Sample size	#14	Estimated number of participants needed to achieve study objectives and how it was determined, including clinical and statistical assumptions supporting any sample size calculations	11
Recruitment	#15	Strategies for achieving adequate participant enrolment to reach target sample size	5
**Methods: Assignment of interventions (for controlled trials)**			
Allocation: sequence generation	#16a	Method of generating the allocation sequence (eg, computer-generated random numbers), and list of any factors for stratification. To reduce predictability of a random sequence, details of any planned restriction (eg, blocking) should be	n/a

For peer review only

		provided in a separate document that is unavailable to those who enrol participants or assign interventions	
Allocation concealment mechanism	#16b	Mechanism of implementing the allocation sequence (eg, central telephone; sequentially numbered, opaque, sealed envelopes), describing any steps to conceal the sequence until interventions are assigned	n/a
Allocation: implementation	#16c	Who will generate the allocation sequence, who will enrol participants, and who will assign participants to interventions	n/a
Blinding (masking)	#17a	Who will be blinded after assignment to interventions (eg, trial participants, care providers, outcome assessors, data analysts), and how	n/a
Blinding (masking): emergency unblinding	#17b	If blinded, circumstances under which unblinding is permissible, and procedure for revealing a participant's allocated intervention during the trial	n/a
**Methods: Data collection, management, and analysis**			
Data collection plan	#18a	Plans for assessment and collection of outcome, baseline, and other trial data, including any related processes to promote data quality (eg, duplicate measurements, training of assessors) and a description of study instruments (eg, questionnaires, laboratory tests) along with their reliability and validity, if known. Reference to where data collection forms can be found, if not in the protocol	12
Data collection plan: retention	#18b	Plans to promote participant retention and complete follow-up, including list of any outcome data to be collected for participants who discontinue or deviate from intervention protocols	12
Data management	#19	Plans for data entry, coding, security, and storage, including any related processes to promote data quality (eg, double data entry; range checks for data values). Reference to where details of data management procedures can be found, if not in the protocol	12
Statistics: outcomes	#20a	Statistical methods for analysing primary and secondary outcomes. Reference to where other details of the statistical analysis plan can be found, if not in the protocol	12

For peer review only

Statistics: additional analyses	#20b	Methods for any additional analyses (eg, subgroup and adjusted analyses)	12
Statistics: analysis population and missing data	#20c	Definition of analysis population relating to protocol non-adherence (eg, as randomised analysis), and any statistical methods to handle missing data (eg, multiple imputation)	12
**Methods: Monitoring**			
Data monitoring: formal committee	#21a	Composition of data monitoring committee (DMC); summary of its role and reporting structure; statement of whether it is independent from the sponsor and competing interests; and reference to where further details about its charter can be found, if not in the protocol. Alternatively, an explanation of why a DMC is not needed	12
Data monitoring: interim analysis	#21b	Description of any interim analyses and stopping guidelines, including who will have access to these interim results and make the final decision to terminate the trial	n/a
Harms	#22	Plans for collecting, assessing, reporting, and managing solicited and spontaneously reported adverse events and other unintended effects of trial interventions or trial conduct	12
Auditing	#23	Frequency and procedures for auditing trial conduct, if any, and whether the process will be independent from investigators and the sponsor	n/a
**Ethics and dissemination**			
Research ethics approval	#24	Plans for seeking research ethics committee / institutional review board (REC / IRB) approval	2
Protocol amendments	#25	Plans for communicating important protocol modifications (eg, changes to eligibility criteria, outcomes, analyses) to relevant parties (eg, investigators, REC / IRBs, trial participants, trial registries, journals, regulators)	2
Consent or assent	#26a	Who will obtain informed consent or assent from potential trial participants or authorised surrogates, and how (see Item 32)	6

For peer review only

Consent or assent: ancillary studies	#26b	Additional consent provisions for collection and use of participant data and biological specimens in ancillary studies, if applicable	6
Confidentiality	#27	How personal information about potential and enrolled participants will be collected, shared, and maintained in order to protect confidentiality before, during, and after the trial	12
Declaration of interests	#28	Financial and other competing interests for principal investigators for the overall trial and each study site	14
Data access	#29	Statement of who will have access to the final trial dataset, and disclosure of contractual agreements that limit such access for investigators	12
Ancillary and post trial care	#30	Provisions, if any, for ancillary and post-trial care, and for compensation to those who suffer harm from trial participation	n/a
Dissemination policy: trial results	#31a	Plans for investigators and sponsor to communicate trial results to participants, healthcare professionals, the public, and other relevant groups (eg, via publication, reporting in results databases, or other data sharing arrangements), including any publication restrictions	2
Dissemination policy: authorship	#31b	Authorship eligibility guidelines and any intended use of professional writers	14
Dissemination policy: reproducible research	#31c	Plans, if any, for granting public access to the full protocol, participant-level dataset, and statistical code	n/a
**Appendices**			
Informed consent materials	#32	Model consent form and other related documentation given to participants and authorised surrogatess	6
Biological specimens	#33	Plans for collection, laboratory evaluation, and storage of biological specimens for genetic or molecular analysis in the current trial and for future use in ancillary studies, if applicable	7

The SPIRIT Explanation and Elaboration paper is distributed under the terms of the Creative Commons Attribution License CC-BY-NC. This checklist was completed on 07. November 2022 using https://www.goodreports.org/, a tool made by the EQUATOR Network in collaboration with Penelope.ai

For peer review only

BMJ Open

Protocol for a multicentre cross-sectional, longitudinal ambulatory clinical trial in rheumatoid arthritis and Parkinson's disease patients analysing the relation between the gut microbiome, fasting and immune status (ExpoBiome)

Journal:	*BMJ Open*
Manuscript ID	bmjopen-2022-071380.R2
Article Type:	Protocol
Date Submitted by the Author:	12-Jul-2023
Complete List of Authors:	Hansen, Bérénice; LCSB<br>Laczny, Cédric C.; LCSB<br>Aho, Velma T.E.; LCSB<br>Frachet-Bour, Audrey; LCSB<br>Habier, Janine; LCSB<br>Ostaszewski, Marek; LCSB<br>Michalsen, Andreas; Charité Universitätsmedizin Berlin, Institute for Social Medicine, Epidemiology and Health Economics; Immanuel Hospital Berlin-Wannsee Branch, Department of Internal and Integrative Medicine<br>Hanslian, Etienne; Charite Universitatsmedizin Berlin, Institute for Social Medicine, Epidemiology and Health Economics; Immanuel Hospital Berlin-Wannsee Branch<br>Koppold-Liebscher, Daniela; Charité Universitätsmedizin Berlin, Institute for Social Medicine, Epidemiology and Health Economics; Immanuel Hospital Berlin-Wannsee Branch<br>Hartmann, Anika; Charité Universitätsmedizin Berlin, Institute of Social Medicine, Epidemiology and Health Economics; Charité Universitätsmedizin Berlin, Department of Dermatology, Venereology and Allergology<br>Steckhan, Nico; Charité Universitätsmedizin Berlin, Institute for Social Medicine, Epidemiology and Health Economics; University of Potsdam, Digital Health - Connected Healthcare, Hasso Plattner Institute<br>Mollenhauer, Brit; University Medical Center Göttingen; Paracelsus-Kliniken Deutschland GmbH<br>Schade, Sebastian; University Medical Center Göttingen; Paracelsus-Kliniken Deutschland GmbH,<br>Roomp, Kirsten; LCSB<br>Schneider, Jochen; LCSB; Saarland University Hospital and Saarland University Faculty of Medicine, Department of Internal Medicine and Psychiatry<br>Wilmes, Paul; LCSB; University of Luxembourg, Department of Life Sciences and Medicine
**Primary Subject Heading**:	Nutrition and metabolism
Secondary Subject Heading:	Immunology (including allergy), Rheumatology, Pharmacology and

For peer review only - http://bmjopen.bmj.com/site/about/guidelines.xhtml

	therapeutics, Neurology, Evidence based practice
Keywords:	IMMUNOLOGY, Rheumatology < INTERNAL MEDICINE, MICROBIOLOGY, Parkinson-s disease < NEUROLOGY, NUTRITION & DIETETICS, Clinical trials < THERAPEUTICS

SCHOLARONE™
Manuscripts

Protocol for a multicentre cross-sectional, longitudinal ambulatory clinical trial in rheumatoid arthritis and Parkinson's disease patients analysing the relation between the gut microbiome, fasting and immune status (ExpoBiome)

Bérénice Hansen[1], Cédric C. Laczny[1], Velma T.E. Aho[1], Audrey Frachet-Bour[1], Janine Habier[1], Marek Ostaszewski[1], Andreas Michalsen[4,5], Etienne Hanslian[4,5], Daniela A. Koppold[4,5,8], Anika Hartmann[4,10], Nico Steckhan[4,9], Brit Mollenhauer[6,7], Sebastian Schade[6,7], Kirsten Roomp[1], Jochen G. Schneider[1,3+], Paul Wilmes[1,2+]

[1]Luxembourg Centre for Systems Biomedicine, University of Luxembourg, Campus Belval, 7, avenue des Hauts-Fourneaux, L-4362 Esch-sur-Alzette, Luxembourg

[2]Department of Life Sciences and Medicine, University of Luxembourg, 7, avenue des Hauts-Fourneaux, L-4362 Esch-sur-Alzette, Luxembourg

[3]Department of Internal Medicine and Psychiatry, Saarland University Medical Center, D- 66421 Homburg Saar, Germany

[4]Institute for Social Medicine, Epidemiology and Health Economics, Charité – Universitätsmedizin Berlin, corporate member of Freie Universität Berlin, Humboldt-Universität zu Berlin, and Berlin Institute of Health

[5]Department of Internal and Integrative Medicine, Immanuel Hospital Berlin, Germany

[6]Paracelsus-Elena-Klinik, Kassel, Germany

[7]University Medical Center Göttingen, Germany

[8]Department of Pediatrics, Division of Oncology and Hematology, Charité – Universitätsmedizin Berlin, Corporate Member of Freie Universität Berlin, Humboldt-Universität zu Berlin and Berlin Institute of Health, Berlin, Germany

[9]Digital Health - Connected Healthcare, Hasso Plattner Institute, University of Potsdam, Potsdam, Germany

[10]Department of Dermatology, Venereology and Allergology, Charité—Universitätsmedizin Berlin, Corporate Member of Freie Universität Berlin and Humboldt-Universität zu Berlin, Berlin, Germany

[+]contributed equally

*Correspondence to:

Jochen Schneider (jochen.schneider@uni.lu)
Paul Wilmes (paul.wilmes@uni.lu)

Luxembourg Centre for Systems Biomedicine,
University of Luxembourg, Campus Belval,
7, avenue des Hauts-Fourneaux,
L-4362 Esch-sur-Alzette, Luxembourg

Word count: 4461

For peer review only

Abstract

Introduction

Chronic inflammatory diseases like rheumatoid arthritis (RA) and neurodegenerative disorders like Parkinson's disease (PD) have recently been associated with a decreased diversity in the gut microbiome, emerging as key driver of various diseases. The specific interactions between gut-borne microorganisms and host pathophysiology remain largely unclear. The microbiome can be modulated by interventions comprising nutrition.

The aim of our clinical study is to (1) examine effects of prolonged fasting and time-restricted eating (TRE) on the outcome parameters and the immunophenotypes of RA and PD with (2) special consideration of microbial taxa and molecules associated with changes expected in (1), and (3) identify factors impacting the disease course and treatment by in-depth screening of microorganisms and molecules in personalised HuMiX gut-on-chip models, to identify novel targets for anti-inflammatory therapy.

Methods and Analysis

This trial is an open-label, multicentre, controlled clinical trial consisting of a cross-sectional and a longitudinal study. A total of 180 patients is recruited. For the cross-sectional study, 60 patients with PD, 60 patients with RA and 60 healthy controls are recruited at two different, specialized clinical sites. For the longitudinal part, 30 patients with PD and 30 patients with RA undergo 5-7 days of prolonged fasting (PF) followed by TRE (16:8) for a period of 12 months. One baseline visit takes place before the PF intervention and 10 follow-up visits will follow over a period of 12 months (April 2021 to November 2023).

Ethics and dissemination

Ethical approval was obtained to plan and conduct the trial from the institutional review board of the Charité-Universitätsmedizin Berlin (EA1/204/19), the ethics committee of the state medical association (Landesärztekammer) of Hessen (2021-2230-zvBO) and the Ethics Review Panel (ERP) of the University of Luxembourg (ERP 21-001-A ExpoBiome). The results of this study will be disseminated through peer-reviewed publications, scientific presentations and social media.

Trial registration number at clinicaltrials.gov:

NCT04847011

Key words: Microbiome, fasting therapy, intermittent fasting, time restricted eating, chronic disease, rheumatoid arthritis, Parkinson's disease, nutrition, chronic diseases, ExpoBiome, inflammation, gut on a chip, HuMiX, immunophenotype, metagenomics, metatranscriptomics, metaproteomics, metabolomics

Strengths and limitations of the study

- The participants of the longitudinal study will be closely monitored for 12 months and routine blood parameters as well as anthropometric data and questionnaires will be precisely documented.

For peer review only

- This study will identify novel microbiome-derived common and disease-associated molecules involved in immune system modulation in two major chronic diseases: RA and PD.
- This study aims at also identifying novel targeted pathways to control chronic inflammatory conditions in the future.
- A limitation is the heterogeneity of the cohorts regarding age and sex, which is due to the prevalence of the diseases: RA is more common in women, while PD is more common in men and has a later disease onset.
- A bias exists in choosing RA and PD as chronic disorders to study immunophenotypes although generalisable results are targeted.

Introduction (1339)

The human microbiome is emerging as a key driver of various diseases through its complex of distinct yet connected biomolecules (referred to as the "*expobiome*")[1, 2]. The expobiome comprises a diverse set of nucleic acids, polypeptides and metabolites which, in the gut alone, are present in substantial concentrations[1]. However, the specific interactions between gut-borne microorganisms and host (patho)physiology remain largely unknown. Although host genetics shape the composition of the gut microbiome, the latter is particularly influenced by non-genetic factors such as lifestyle and diet[3, 4]. Therefore, the microbiome is a plausible target to modify health outcomes.

Individuals suffering from chronic diseases, including autoimmune, metabolic, and neurodegenerative diseases as well as cancer, often present alterations in their gut microbiome composition. These shifts are typically characterised by an overgrowth of one or several microbial species with likely adverse effects as well as a decrease in beneficial taxa[5]. Such imbalances are referred to as dysbiosis. Although structural microbiome changes are clearly detectable, the mechanistic or functional consequences of dysbiosis are still largely unknown. However, they may result in dysregulated interactions with the immune system[6]. Considering the intricacy of the immune system, the question arises whether the observed microbiome changes are cause or consequence of disease. This implies that, in addition to the genetic predisposition of the host, the gut microbiome needs to be considered a potential pathogenic factor or major driver of disease onset and course[3, 4].

RA and PD are two specific examples representing dysregulated microbiome-immune system interactions [7, 8]. RA is a multifactorial, chronic, and systemic autoimmune disease, primarily affecting the lining of the synovial joints with a higher risk and younger age for disease onset in women and a global prevalence of 1%[9, 10]. The exact disease pathogenesis is still unclear and no cure for RA currently exists. In addition to the common local articular symptoms of RA, systemic comorbidities can affect the vasculature, metabolism and bones[11]. Besides various environmental risk factors e.g. smoking and a Western diet, the host microbiome is associated with the pathophysiology of the disease[12]. The diversity of the gut microbiome has been reported to be decreased in individuals with RA, compared with the general population, and is correlated with disease duration, activity, and autoantibody levels[13, 14]. Studies in murine models also report that autoimmune arthritis is strongly attenuated under germ-free conditions[15]. The introduction of specific bacteria, e.g. segmented filamentous bacteria, into germ-free animals or oral infection with *Porphyromonas gingivalis* drive autoimmune arthritis through activation of T helper cells[15]. Several different taxa, including *Prevotella copri*, *Lactobacillus* spp. and *Colinsella* spp. are enriched in the gut microbiome of patients with RA and correlate positively with disease markers e.g. immunoglobulins IgA and IgG , while other taxa like *Haemophilus* spp. and *Faecalibacterium* spp. are typically found at lower abundances in

For peer review only

patients with RA compared to healthy individuals[13, 16, 17]. Alterations of the gut microbiome may therefore have an important impact on RA pathophysiology[12].

PD affects 0.4-2% of the population over 65 years worldwide and is the second most common progressive neurodegenerative disease with men being 1.5 times more likely to be affected than women[18]. Cardinal symptoms are motor deficiencies such as tremor and rigidity, but also include a wide range of non-motor symptoms, such as hyposmia, depression, insomnia or cognitive impairment, severely impacting patients' quality of life[19]. Aggregations of the protein α-synuclein in the dopaminergic substantia nigra represent the main neuropathological manifestations[20]. PD-associated loss of dopaminergic neurons involves mechanisms of inflammatory and autoimmune responses with microglial activity as a major driver [21].Dysbiosis of the gut microbiome has been associated with the characteristic motor deficits and pathophysiological changes in the enteric and central nervous systems in animal studies. Increased relative abundances of the genera *Akkermansia, Bifidobacterium, Lactobacillus,* and *Methanobrevibacter* and decreased abundances in *Faecalibacterium* and *Roseburia* have been reported[22, 23]. Two recently published clinical-trials with prebiotic supplementation in PD observed a shift in gut microbiome composition, an increase in short-chain fatty acids (SCFA) and a reduction in non-motor-symptoms [24, 25]. Most patients with PD suffer from gastrointestinal symptoms such as constipation and irritable bowel syndrome (IBS) -like symptoms[26]. The gut-brain axis, e.g. by-products produced by the gut microbiome, may contribute to the production of α-synuclein aggregates in the enteric nervous system[27]. In addition, increased intestinal permeability[28] as driver for enteric inflammation occur in PD and substantiate a role of peripheral inflammation in the initiation and the progression of the disease[29].

One factor with known major impact on the gut microbiome and on chronic diseases is diet[7]. Dietary approaches as fasting have already been used by Hippocrates in the 5th century BCE and have been applied ever since by numerous medical schools to treat acute and chronic diseases [30-32]. Various practices of caloric restriction through fasting have repeatedly shown remarkable health benefits[33, 34]. Maifeld *et al.* found that a 5-day fast followed by a modified Dietary Approach to Stop Hypertension (DASH), with additional emphasis on plant-based and Mediterranean diets, reduced systolic blood pressure, BMI, and the need for antihypertensive medications at three months post intervention compared with DASH alone [35].

Furthermore, Choi *et al.* demonstrated that cycles of a fasting-mimicking diet suppress autoimmunity and stimulate remyelination via oligodendrocyte regeneration in a murine experimental autoimmune encephalomyelitis (EAE) model[36]. Jordan *et al.* described a reduction in monocyte metabolic and inflammatory activity after a short-term fast and conclude that fasting attenuates chronic inflammatory diseases without compromising monocyte capacity for mobilisation during acute infectious inflammation and tissue repair[37].

These improvements can, however, typically only be maintained for a limited period of time, and the symptoms can reappear after reintroduction of the patients' standard diet. Hence, protocols to sustain these beneficial effects are of utmost importance. In mouse models of PD, intermittent fasting (IF) has led to several improvements including decreased excitotoxicity, reduced neurodegeneration and protection against autonomic dysfunction, motor and cognitive decline[38].

IF and PF may have potent immunomodulatory effects which may partially be mediated by the gut microbiome and the fasting induced alterations of the latter[39]. These microbial shifts include upregulation of *Akkermansia muciniphila*, *Bacteroides fragilis*, other *Bacteroides* spp., Proteobacteria, and butyric acid producing *Lachnospiraceae,* but also *Odoribacter*, which is negatively associated with blood pressure[35, 40]. Interestingly, an overall decrease of the Firmicutes/Bacteroidetes ratio could be observed, a high ratio is commonly associated with several pathologies, including RA [41].

For peer review only

A potential mechanism underlying the observed beneficial effects induced by dietary interventions might be a direct gut microbiome-immune system interaction by pattern recognition. The microbiome can regulate the intestinal innate immune system by modulating toll-like receptor (TLR) expression on immunosensor cell surface through microbe-associated molecular patterns (MAMPs), which can consequently trigger cytokine production and up-regulation of molecules on antigen presenting cells, leading to activation of T cells[42]. Therefore, a change in gut microbiome composition can lead to different outcomes in immune signalling pathways and either favour or suppress inflammation and autoimmunity.

The impact and importance of the gut microbiome on human physiology and its potential modifications by nutrition and dietary patterns, have been underestimated for centuries[43]. Reasons may include missing standardised therapeutic protocols, the interindividual variability in the response to fasting, lack of knowledge about possible adverse effects, and difficulties in the interpretation of underlying mechanisms seen in clinical trials, but also in the comparably low potential for achieving economic revenue or scientific impact[8].

Modern experimental approaches and computational integration allow a multi-layer analysis of digestive processes in low caloric settings including the gut microbiome[44]. These technological developments also permit a closer investigation of the link between the immune system and severe caloric restriction.

To our knowledge no clinical trials have been investigating the connection between IF or PF and PD in humans so far[38]. Our study aims to elucidate the causal relationship between the gut microbiome and the immune system. To do so, we will use analyses of the molecular basis of human-microbiome interactions enabled by high throughput methodologies such as the combination of metagenomics, metatranscriptomics and metaproteomics. Moreover, we are aiming at identifying new genes, proteins, metabolites, and host pathways facilitating the development of novel diagnostic and therapeutic tools[45, 46].

Methods and Analysis (3122)

Study objectives

The first objective of the study is to define specific gut microbiome-derived molecules in RA and PD, compared to healthy individuals, and relate this information to the immunophenotypes of the individuals. The second objective is to identify and track common and disease-specific molecular signatures to predict the outcome of a gut microbiome-targeted therapeutic intervention, here fasting, on inflammation-driven symptoms in RA and PD. The third objective of the study is to identify and validate microbiome-derived effector molecules which downregulate pro-inflammatory innate and adaptive immune pathways.

Study design

The ExpoBiome cohort consists of 180 adult individuals, meeting the exclusion and inclusion criteria (Table 1), for the cross-sectional study (objectives 1 and 3) and 60 adult individuals for the longitudinal study (objectives 2 and 3). There are five different arms in total: (1) RA – cross-sectional arm [60 patients], (2) PD – cross-sectional arm [60 patients], and (3) healthy controls – cross-sectional arm [60 patients], (4) RA – longitudinal arm [30 patients], (5) PD – longitudinal arm [30 patients] (Figure1).

For peer review only

At the first visit (T0), patients answer several questionnaires, and blood, urine, saliva, and stool samples are obtained (Table 2).

The longitudinal arms (4) and (5) undergo a 5–7-day PF with a dietary energy supply of max. 350-400 kcal per day with vegetable or grain broths as well as fresh vegetable juices[31, 40]. After the PF, the longitudinal arms follow a dietary regimen including the concept of TRE for a period of 12 months following the 16:8 pattern[47]. This means that food intake is allowed ad libitum for 8 h, followed by 16 h of fasting where no food should be consumed. The intake of non-caloric beverages, e.g., water, unsweetened tea or coffee is, however, allowed. The participants attend one follow-up visit (T2) during the PF and 9 follow-up visits during the 12 months of TRE (Figure 1).

Patient and Public Involvement

Feedback of patients during former clinical trials at the study centre in Berlin was integrated in the planning and design of the fasting intervention of this study. Patients are not involved in the conduct, reporting, or dissemination plans of this research.

Recruitment and randomisation

Patients are recruited by the specialised sites via different sources, e.g., by direct referral from either a physician at the Immanuel Hospital Berlin and the outpatient department of the Institute of Social Medicine, Epidemiology and Health Economics at Charité-Universitätsmedizin Berlin, or the Paracelsus-Elena Clinic in Kassel, or by non-personal advertising strategies (e.g. flyers or social media).

For PD, the patients are screened by an experienced movement disorders specialist for featuring at least two of resting tremor, bradykinesia, and rigidity according to the United Kingdom Parkinson's Disease Society Brain Bank criteria[48]. Additionally, patients must show evidence of a dopaminergic deficit, either with DaTScan imaging or with a clear response to dopaminergic drugs. Motor and non-motor symptoms are assessed with the MDS-UPDRS (part I – IV) including the Hoehn and Yahr (severity) scale[49]. Additional PD-specific scales as Parkinson's Disease Sleep Scale-2, Parkinson's Disease Questionnaire-39, Non-Motor Symptoms Questionnaire and Non-Motor Symptoms Scale are used.

For patients with RA, the diagnosis has been made prior to the study by an experienced rheumatologist according to the European League Against Rheumatism (EULAR) criteria[50]. All clinical stages of RA will be included. We excluded patients with a BMI <18.5, as this indicates underweight, and fasting is not recommended. We did, however, not include an upper limit as fasting might be especially beneficial for patients with a BMI >24.9 and more than 60% of patients with RA are classified as overweight or obese[51]. For comorbidities we excluded mainly diseases which are known to interfere with the gut microbiome and might be potential confounders.

The chosen exclusion criteria will optimize the pairing process of healthy controls and patients with either RA or PD. However, as we have two diseases with different anthropometric characteristics (including age, gender, BMI) and only one control group, adding additional inclusion and exclusion criteria in the recruitment process would compromise on optimized matching. Furthermore, for the longitudinal part of the study, each patient will serve as his/her own control over time. Participants meeting all the inclusion and no exclusion criteria (Table 1) are assigned to their respective groups (RA, PD, or healthy control) (Figure 1) for the cross-sectional study after written informed consent.

Half of the patients from the RA group and half of the patients from the PD group is selected to take part in the longitudinal part of the study, including the fasting intervention according to their availability for all 11 visits and their willingness to follow TRE over 12 months. This study is an open-label trial, as blinding is not feasible in fasting interventions.

Table 1: Inclusion and exclusion criteria

Inclusion criteria	Exclusion criteria

For peer review only

• Age 18-79<br>• One of the following diagnoses: rheumatoid arthritis (first diagnosis >6 weeks ago), Parkinson's disease OR healthy volunteer<br>• Control ("healthy") individuals must be without any evidence of active known or treated RA, without any evidence of active, known or treated central nervous system disease, and without a known family history of idiopathic PD<br>• Control individuals should match the RA or PD individuals as closely as possible (sex, age, education)<br>• Present written declaration of consent<br>• Ability to understand the patient information and willingness to sign the consent form<br>• Consent to specimen collection and specimen use	• Gout or proven bacterial arthritis<br>• Participation in another study<br>• Existing/current eating disorder (bulimia nervosa, anorexia nervosa) within the past 5 years<br>• Severe internal disease (e.g. kidney deficiency with creatinine > 2mg/dl)<br>• Existing vegan diet or fasting during the last six months<br>• Presence or suspicion of atypical PD (e.g. early dementia, early autonomous dysfunction)<br>• Diagnosis of chronic inflammatory bowel diseases, celiac disease or colorectal cancer according to the guidelines of the German Society of Gastroenterology<br>• Use of anti-psychotic drugs<br>• Antibiotic use during the previous 12 months<br>• Start of novel therapy with disease-modifying anti-rheumatic drugs<br>• Pregnancy or breastfeeding women<br>• Contraindication for additional blood draws (e.g. haemoglobin <10)<br>• BMI < 18.5<br>• Psychiatric illness that limits understanding of the examination protocol (unable to consent)

Fasting dietary counselling

The fasting group is closely monitored by nutritionists trained in fasting therapy, backed up by physicians experienced in fasting, from the Charité – Universitätsmedizin Berlin and the Paracelsus-Elena Clinic to ensure a uniform implementation of the fasting guidelines and the well-being of the study participants. The monitoring consists of several in person and virtual meetings which held individually or in group settings. Five meetings including the visits T0 and T2 during the fasting week as well as a group meeting after PF to ensure a well-managed start to the TRE phase take place. Group sessions are standardised using a pre-set deck of slides to be discussed during the group meetings with only minor disease-related differences between the PD and RA groups. All longitudinal participants receive a study-specific script with information on fasting procedures. Although the adherence of the patients cannot be profoundly controlled in the ambulatory setting, the blood samples will allow us to have additional insight into the nutritional habits as well as the fasting state of the patients on the day of the visit (blood glucose levels).

For peer review only

Medication

The medical treatments of the patients are monitored and documented with every clinical visit. The fasting intervention might necessitate temporary adjustments of several medications e.g., anti-diabetic and anti-hypertensive drugs as insulin levels and hypertension will be reduced due to lack of food intake [31].

Data collection

Sample and data collection is performed at the two clinical sites, Charité – Universitätsmedizin Berlin and Paracelsus-Elena Clinic (Table 2).

Table 2: Sampling procedures.

a) Biochemical samples and procedures

Blood (123 mL at T0, 23 mL at T2-T11)
Stool collection (2 mL at T0 and T3-T11)
Saliva collection (3.5 mL at T0-T11)
Midstream urine (50 mL at T0 -T11)

b) Questionnaires

Disease specific
PD:<br>• Disease Activity Score (DAS-28) [52]<br>• Parkinson's Disease Sleep Scale-2 (PDSS-2) [53]<br>• Parkinson's Disease Questionnaire-39 (PDQ-39)[54]<br>• Simplified Disease Index Score (SDAI) [55]<br>• Funktionsfragebogen Hannover (FFbH-R) [56]<br>• Movement Disorder Society Unified PD Rating Scale (MDS-UPDRS)[57]<br>• Non-Motor Symptoms Questionnaire (NMSQ)[58]<br>• Non-Motor Symptoms Scale (NMSS)[59]<br>RA:<br>• Disease Activity Score (DAS-28) [55]

For peer review only

• Non-Motor Symptoms Questionnaire (NMSQ) [58]<br>• Funktionsfragebogen Hannover (FFbH-R) [56]
Dietary behaviour and lifestyle
• Fasting experience, expectation, and intervention<br>• Lifestyle<br>• 24H-Food-recall<br>• Food Frequency Questionnaire (FFQ)
General health and well-being
• Health Assessment Questionnaire (HAQ)[60]<br>• Bristol Stool Scale[61]<br>• Quality of Life questionnaire (WHO-5)[62]<br>• Hospital Anxiety and Depression Scale (HADS)[63]<br>• Profile of Mood States[64]

Anthropometric data and questionnaires

The electronic data capture system REDCap[65], a secure web-based application, is used to record all individual specific data. All data is stored on a secure server infrastructure at the host institution in Luxembourg. Weight, height, body mass index (BMI), heart rate and blood pressure in sitting and standing position as well as waist-hip-ratio is determined at every visit. Dietary behaviour, sociodemographic measurements (age, sex, education level, employment status, marital status), family history, current and previous illness and co-morbidities, and current medications, as well as disease-specific data, questionnaires about the well-being of the patients and data on the behavioural factors are collected at baseline, T6 (week 3), T9 (month 6) and T11 (month 12) (Table 2). Questionnaires (24h-Food Recall, Bristol Stool Scale) are answered at all visits by the study participants. Data storage, analysis and exchange are done only in pseudonymised fashion. The nutritional data is analysed using the Nutrilog 3.20 software (Nutrilog SAS, Marans).

Blood samples and parameters

Blood samples are collected at each visit, and immediately used for peripheral blood mononuclear cell (PBMC) isolation (T0), analysis by the study laboratory and centrifugation to freeze plasma samples at -80°C (T0-T11). A clinical standard laboratory report is generated after every visit for each study participant (Table 3). In addition to routine blood parameters, anti-citrullinated protein antibody

For peer review only

(ACPA), zonulin, fatty-acid binding protein 2 (FABP2), and calprotectin levels are measured. Aliquots are securely stored to account for novel observations and testing of hypotheses.

Table 3: Routine blood parameters measured at each timepoint (T0 for cross-sectional study, T0-T11 for longitudinal study)

Haematology – EDTA-blood	Clinical Chemistry – Serum
Basophils, %	Albumin
Basophils, abs.	ALT, 37°C
Eosinophils, %	Alkaline Phosphatase, 37°C
Eosinophils, abs.	AST, 37°C
Erythrocytes	Bilirubin, total
Haematocrit	Cholinesterase
Haemoglobin	Cholesterol
HbA1c	Creatinine
Leucocytes	hs-CRP
Lymphocytes, %	Glucose, serum
Lymphocytes, abs.	Gamma-GT, 37°C
MCH	HDL-Cholesterol
MCHC	LDL-Cholesterol
MCV	Potassium
Monocytes, %	Sodium
Monocytes, abs.	Total Protein
Neutrophils, %	Triglycerides
Neutrophils, abs.	Uric Acid
Platelets	Urea/BUN
RDW	**Proteins – Serum**
Reticulocytes	Rheumatoid factor H 35.9
Reticulocytes	**Hormones – Serum**
Reticulocytes, abs.	Insulin
	TSH (basal)

Stool, urine and saliva samples

The samples listed in Table 2 are collected at each visit, except for stool samples on T2 (fasting week) and immediately frozen and stored at -80°C. Stool characteristics are recorded at the time of the sampling. Faecal samples represent the main sample type for resolving the dynamic processes driven by microbiome in the gut. Also, as the gut microbiome is prone to diurnal fluctuations, the stool samples are collected in the morning, as far as possible.

For peer review only

Methods applied to samples

Biomolecular extractions

The collected stool samples undergo a biomolecular extraction procedure to allow isolation of concomitant DNA, RNA, proteins, peptides and metabolites from single, unique faecal water samples; this process involves cryo-milling the samples in liquid nitrogen, disassociating metabolites from membrane and cell wall components in a solvent mixture of methanol, chloroform and water and lastly proteins and RNA extraction by a methanol/chloroform and phenol buffer [66, 67]. Faecal water is recovered following centrifugation and filtration, at low-speed or low-flow, respectively, to avoid cell lysis. Nucleic acids are preserved by the addition of ribonuclease inhibitors and isolated by silica-column-based techniques. This protocol involves the use of a robotic platform, ensuring a higher level of standardisation and reproducibility[2].

Coupled metagenomic and metatranscriptomic analyses

Prior to sequencing library preparation, internal standards are introduced to obtain quantitative sequencing data[68]. Contamination-free metagenomic (MG) and metatranscriptomic (MT) data is generated, processed and analysed using the Integrated Meta-omics Pipeline (IMP)[45], which incorporates pre-processing, assembly, gene annotation, mapping of reads, single nucleotide polymorphism calling, data normalisation as well as analyses of community structure and function in a fully reproducible software framework based on Docker. The MG and MT data is specifically screened for enrichments in genes and pathways with known immunogenic properties[69]. The extracellular biomolecules are linked to specific microbial populations based on the intracellular metagenomic data [70]. In addition, the sequencing data is mapped against genomes of food components[44]. The quantitative data is also related to microbial population sizes to determine the contribution of the resolved microbial populations in stool to the extracellular DNA and RNA complements[71].

Metaproteomics

For the metaproteomic analyses, filtration is used to separate extracellular peptides from the obtained (poly)peptides. The resulting smaller fractions are then desalted and analysed without proteolytic digestion via liquid chromatography (LC) and mass spectrometry (MS) on an EasyNano-LC coupled online to a QExactive-Plus mass spectrometer (ThermoScientific, Waltham, USA). The identification of ribosomal peptides is done with an integrated catalogue of MG and MT data, while the non-ribosomal peptides are identified using different tools, i.e., MyriMatch, DirecTag as well as CycloBranch[45, 72, 73]. The metaproteomic data also allows identification of extracellular (poly)peptides with possible pathogenic functions including protein misfolding and molecular mimicry[74, 75].

Metabolomics

Metabolomic data is analysed using a combination of targeted and untargeted approaches [44, 67, 76]. This highlights the major metabolite classes produced by the gut microbiome with an effect on human physiology including organic acids, SCFA, lipids, branched-chain fatty acids, branched-chain amino acids, vitamins, bile acids and neurotransmitters. Besides external compound calibration series for quantification and quality control samples to ensure data normalisation and data acquisition quality assessment, the metabolite extraction fluid is fortified with multiple internal standards to improve method precision and accuracy[77, 78]. The data is compared to in-house databases and public mass spectral libraries to identify known metabolites. The metabolomic data complements the

For peer review only

metagenomic and metatranscriptomic data and thus allows further establishments of conclusive links to metabolic properties in the gut.

Deep immune profiling

Deep immune profiling is done using a recently established and optimised panel of metal-labelled antibodies together with cytometry coupled to mass spectrometry (MS), the Maxpar Direct Immune Profiling System (MDIPA). This approach allows the simultaneous quantification of 38 parameters on single cells. Whole blood is stained with the MDIPA kit and stabilised with Proteomic stabiliser Prot-1 (501351694, Smart Tube Inc., Las Vegas) before storage at -80°C. The quantified immune cells included in the MDIPA panel are CD3+, CD4+, CD8+, monocytes, dendritic cells, granulocytes, MAIT, T cells, NK and B cells[79]. Cytokine expression profiles is analysed on blood plasma using the Human Luminex performance Cytokine Panel (R&D Systems Europe, Abingdon), measuring CCL3, CCL4, CCL5, GM-CSF, IL-1β, IL-2, IL-4, IL-5, IL-6, IL-8, IL-10, IL-12p70, IL-13, IL-15, IL-18, IL-21, IL-27, IL-33, IFN-β, Galectin-1, IFN-γ and TNF-α [69].

Gut-on-a-chip models

PBMCs isolated from T0 blood samples are co-cultured with gut-derived microbes under physiologically representative conditions using the gut-on-a-chip model HuMiX[80]. This model of the human gastrointestinal interface allows the investigation of the interactions between immune, epithelial and bacterial cells and specifically the response to fasting in personalised in vitro models.

The Expobiome Map

The Expobiome Map (https://expobiome.lcsb.uni.lu) illustrates the diverse complex of microbial immunogenic molecules, including nucleic acids, (poly)peptides, structural molecules, and metabolites. The interactions between this "expobiome" and human immune pathways are encoded in the context of chronic diseases[1]. The ExpoBiome Map is visualised using the MINERVA Platform[81]. Clicking on different elements on the map reveals factors they affect and are affected by, allowing an easier navigation through the complex relationships between individual microbiome components in relation to human disease. The multi-omics data generated in the present study will be integrated with the Map.

Exploratory analysis of novel host-microbiome interactions

Unknown non-ribosomal peptides or metabolite features are associated through correlation with transcripts, proteins, and metabolites. Extracellular DNA fragments, as well as transcripts, proteins and ribosomal peptides are linked to their genomic context by using IMP[45]. The data generated by the project will be connected and collated to existing, publicly available datasets.

Outcome parameters

Primary Outcome

The primary endpoint of the study is the characterisation of the gut microbiome. The evaluation includes both between-group and within-group differences in the longitudinal study arms with the fasting intervention.

Secondary Outcome Measures

Secondary outcomes include the identification of common and disease-specific molecular signatures and the characterisation of microbiome-derived effector molecules impacting the innate and adaptive

For peer review only

immune pathways. Furthermore, several additional parameters mentioned in *Anthropometric data and questionnaires* are assessed over a period of 12 months.

Sample size and power calculation

A power calculation using pilot metatranscriptomic data based on faecal extracellular RNA samples was performed to determine the number of subjects to be recruited for the ExpoBiome project. The obtained relative abundances of genera were used for the calculation of the required sample size per group. The power calculation was based on the algorithm as described by Tusher, Tibshirani, and Chu[82]. To achieve a power of 90% (at $\alpha = 0.05$), a total of 50 individuals per group (RA, PD, healthy controls) must be analysed. Considering any possible dropouts, 20% additional subjects are recruited, resulting in a total number of 180 individuals, i.e., 60 per group. For the longitudinal part, a subset of 60 adult individuals (30 patients with Parkinson's disease and 30 patients with rheumatoid arthritis) are selected, based on their ability and willingness to participate in the longitudinal part of the study (12 months follow-up). The selected number of participants for the longitudinal study is based on feasibility due to the complexity and high costs of the clinical trial. The total number of subjects in the longitudinal study can be smaller, as each individual serves as their own control.

Adverse events

There are no major risks expected for participants. Minor common adverse effects of PF might include headaches, nausea, insomnia, back pain, dyspepsia and fatigue[83]. Any occurring adverse events are recorded at each visit in REDCap[65]. Serious adverse events are communicated to the study coordinator and principal investigator within 24 h of their report.

Data management, monitoring, analysis, and evaluation of data

The study participants receive a study ID (pseudonym) which is used for all collected data. Self-administered questionnaires are directly recorded in REDCap. Participant files are kept for at least 10 years at the respective clinical sites.

Weekly meetings between the study team, the different clinical partners, and the principal investigator, ensure a close monitoring of the data. Any occurring adverse events or other issues are thus handled immediately.

Different statistical tests are performed according to the nature of the data. A premature termination of the study is not envisaged; therefore, no interim analysis is done. Different correlation measures are applied, including Spearman correlation, mutual information on discretised data, distance correlation, maximum information criterion, local similarity analysis and the bioenv approach. Comparison across all omic levels allows identification of common and disease-specific signatures. Multivariate machine learning is used to link different data features to observed patterns. For additional confounding factors, especially in the cross-sectional study, multivariate statistical analysis will be performed. These factors will be accounted for by including confounders in the analysis, e.g., as covariate in the statistical models.

The longitudinal part of the study continues for a period of 12 months. After finalisation of this period, there is no follow-up of the participants. Interesting findings will be further validated using the existing sample set and analyses may be performed on additionally collected samples.

The SPIRIT - checklist (Standard Protocol Items: Recommendations for Interventional Trials) was used to write this protocol [84].

Trial status

The recruitment for the ExpoBiome study started in April 2021 and is currently ongoing. All study participants should be recruited by the end of 2022. The sample collection will take place from April 2021 to November 2023.

Acknowledgements

We thank Dr. Catharina Delebinski, Melanie Dell'Oro, Grit Langhans, Ursula Reuß, Maik Schröder and Nadine Sylvester for their support during the study.

Author contributions:

Study design and protocol were done by Bérénice Hansen, Cédric C. Laczny, Jochen G. Schneider, Paul Wilmes; the interventional concept was drawn by Etienne Hanslian, Daniela Liebscher, Andreas Michalsen, Anika Hartmann, Brit Mollenhauer, Sebastian Schade, Nico Steckhan, Jochen G. Schneider, Paul Wilmes; the clinical trial was designed and is conducted by Etienne Hanslian, Daniela Liebscher, Andreas Michalsen, Anika Hartmann, Brit Mollenhauer, Sebastian Schade; the procured funding was provided by Paul Wilmes; the planning of high-throughput applications, statistical planning, sample size calculation and randomisation were defined by Cédric C. Laczny, Jochen G. Schneider, Paul Wilmes, Kirsten Roomp; the initial draft of the manuscript and coordination of the editing process were performed by Bérénice Hansen; the protocol preparation has been done by Bérénice Hansen, Audrey Frachet-Bour, Janine Habier; the planning of the data analysis was done by Cédric C. Laczny, Jochen G. Schneider, Paul Wilmes, Kirsten Roomp, Velma T.E. Aho, Marek Ostaszewski; all authors contributed equally with edits, comments and feedback, read and approved the final manuscript.

Ethics and dissemination

Ethical approval was obtained to plan and conduct the trial from the institutional review board of the Charité-Universitätsmedizin Berlin (EA1/204/19), the ethics committee of the state medical association (Landesärztekammer) of Hessen (2021-2230-zvBO) and the Ethics Review Panel (ERP) of the University of Luxembourg (ERP 21-001-A ExpoBiome). The results of this study will be disseminated through peer-reviewed publications, scientific presentations, as well as press releases and social media postings (Twitter, LinkedIn). Study participants will be contacted and informed by the respective clinical sites about the outcome and results of the study, once the data analysis has been completed (dissemination phase).

Funding statement: This project has received funding from the European Research Council (ERC) under the European Union's Horizon 2020 research and innovation program (grant agreement No. 863664).

Competing interests statement

None declared.

For peer review only

Supplements

The SPIRIT checklist was used to write our report[84].

References

1. Wilmes, P., et al., *The gut microbiome molecular complex in human health and disease.* Cell Host Microbe, 2022. **30**(9): p. 1201-1206.
2. De Saedeleer, B., et al., *Systematic characterization of human gut microbiome-secreted molecules by integrated multi-omics.* ISME Communications, 2021. **1**(1): p. 82.
3. Greenhalgh, K., et al., *The human gut microbiome in health: establishment and resilience of microbiota over a lifetime.* Environ Microbiol, 2016. **18**(7): p. 2103-16.
4. Hall, A.B., A.C. Tolonen, and R.J. Xavier, *Human genetic variation and the gut microbiome in disease.* Nature Reviews Genetics, 2017. **18**(11): p. 690-699.
5. Baldini, F., et al., *Parkinson's disease-associated alterations of the gut microbiome predict disease-relevant changes in metabolic functions.* BMC Biol, 2020. **18**(1): p. 62.
6. Yoo, J.Y., et al., *Gut Microbiota and Immune System Interactions.* Microorganisms, 2020. **8**(10).
7. Sonnenburg, J.L. and F. Bäckhed, *Diet–microbiota interactions as moderators of human metabolism.* Nature, 2016. **535**(7610): p. 56-64.
8. Zmora, N., J. Suez, and E. Elinav, *You are what you eat: diet, health and the gut microbiota.* Nat Rev Gastroenterol Hepatol, 2019. **16**(1): p. 35-56.
9. Guo, Q., et al., *Rheumatoid arthritis: pathological mechanisms and modern pharmacologic therapies.* Bone Res, 2018. **6**: p. 15.
10. Healthline, V.L. *Rheumatoid Arthritis by the Numbers: Facts, Statistics, and You*. 2021.
11. Scherer, H.U., T. Häupl, and G.R. Burmester, *The etiology of rheumatoid arthritis.* Journal of Autoimmunity, 2020. **110**: p. 102400.
12. Bodkhe, R., B. Balakrishnan, and V. Taneja, *The role of microbiome in rheumatoid arthritis treatment.* Ther Adv Musculoskelet Dis, 2019. **11**: p. 1759720x19844632.
13. Chen, J., et al., *An expansion of rare lineage intestinal microbes characterizes rheumatoid arthritis.* Genome Medicine, 2016. **8**(1): p. 43.
14. Kitamura, K., et al., *Oral and Intestinal Bacterial Substances Associated with Disease Activities in Patients with Rheumatoid Arthritis: A Cross-Sectional Clinical Study.* J Immunol Res, 2022. **2022**: p. 6839356.
15. Wu, H.J., et al., *Gut-residing segmented filamentous bacteria drive autoimmune arthritis via T helper 17 cells.* Immunity, 2010. **32**(6): p. 815-27.
16. Scher, J.U., et al., *Expansion of intestinal Prevotella copri correlates with enhanced susceptibility to arthritis.* eLife, 2013. **2**: p. e01202.
17. Zhang, X., et al., *The oral and gut microbiomes are perturbed in rheumatoid arthritis and partly normalized after treatment.* Nature Medicine, 2015. **21**(8): p. 895-905.
18. Lubomski, M., et al., *Parkinson's disease and the gastrointestinal microbiome.* J Neurol, 2020. **267**(9): p. 2507-2523.
19. Opara, J., et al., *Motor assessment in Parkinson`s disease.* Ann Agric Environ Med, 2017. **24**(3): p. 411-415.

For peer review only

20. Tysnes, O.B. and A. Storstein, *Epidemiology of Parkinson's disease.* J Neural Transm (Vienna), 2017. **124**(8): p. 901-905.
21. Garcia, P., et al., *Neurodegeneration and neuroinflammation are linked, but independent of alpha-synuclein inclusions, in a seeding/spreading mouse model of Parkinson's disease.* Glia, 2022. **70**(5): p. 935-960.
22. Heintz-Buschart, A. and P. Wilmes, *Human Gut Microbiome: Function Matters.* Trends Microbiol, 2018. **26**(7): p. 563-574.
23. Romano, S., et al., *Meta-analysis of the Parkinson's disease gut microbiome suggests alterations linked to intestinal inflammation.* npj Parkinson's Disease, 2021. **7**(1): p. 27.
24. Becker, A., et al., *Effects of Resistant Starch on Symptoms, Fecal Markers, and Gut Microbiota in Parkinson's Disease - The RESISTA-PD Trial.* Genomics Proteomics Bioinformatics, 2022. **20**(2): p. 274-287.
25. Hall, D.A., et al., *An open label, non-randomized study assessing a prebiotic fiber intervention in a small cohort of Parkinson's disease participants.* Nat Commun, 2023. **14**(1): p. 926.
26. Mertsalmi, T.H., et al., *More than constipation - bowel symptoms in Parkinson's disease and their connection to gut microbiota.* European journal of neurology, 2017. **24**(11): p. 1375-1383.
27. Dogra, N., R.J. Mani, and D.P. Katare, *The Gut-Brain Axis: Two Ways Signaling in Parkinson's Disease.* Cell Mol Neurobiol, 2022. **42**(2): p. 315-332.
28. Forsyth, C.B., et al., *Increased intestinal permeability correlates with sigmoid mucosa alpha-synuclein staining and endotoxin exposure markers in early Parkinson's disease.* PLoS One, 2011. **6**(12): p. e28032.
29. Devos, D., et al., *Colonic inflammation in Parkinson's disease.* Neurobiol Dis, 2013. **50**: p. 42-8.
30. Britannica, *The Editors of Encyclopaedia. "fasting".* Encyclopedia Britannica,, Accessed 3 October 2022. https://www.britannica.com/topic/fasting.
31. Hartmann, A.M., et al., *Efficacy of therapeutic fasting and plant-based diet in patients with rheumatoid arthritis (NutriFast): study protocol for a randomised controlled clinical trial.* BMJ Open, 2021. **11**(8): p. e047758.
32. Michalsen, A., *Prolonged fasting as a method of mood enhancement in chronic pain syndromes: a review of clinical evidence and mechanisms.* Curr Pain Headache Rep, 2010. **14**(2): p. 80-7.
33. Vargas-Molina, S., et al., *Effects of a low-carbohydrate ketogenic diet on health parameters in resistance-trained women.* Eur J Appl Physiol, 2021. **121**(8): p. 2349-2359.
34. Mattison, J.A., et al., *Caloric restriction improves health and survival of rhesus monkeys.* Nature Communications, 2017. **8**(1): p. 14063.
35. Maifeld, A., et al., *Fasting alters the gut microbiome reducing blood pressure and body weight in metabolic syndrome patients.* Nature Communications, 2021. **12**(1): p. 1970.
36. Choi, I.Y., et al., *A Diet Mimicking Fasting Promotes Regeneration and Reduces Autoimmunity and Multiple Sclerosis Symptoms.* Cell Rep, 2016. **15**(10): p. 2136-2146.
37. Jordan, S., et al., *Dietary Intake Regulates the Circulating Inflammatory Monocyte Pool.* Cell, 2019. **178**(5): p. 1102-1114.e17.

For peer review only

38. Neth, B.J., et al., *The Role of Intermittent Fasting in Parkinson's Disease.* Front Neurol, 2021. **12**: p. 682184.
39. Cignarella, F., et al., *Intermittent Fasting Confers Protection in CNS Autoimmunity by Altering the Gut Microbiota.* Cell Metab, 2018. **27**(6): p. 1222-1235.e6.
40. Mesnage, R., et al., *Changes in human gut microbiota composition are linked to the energy metabolic switch during 10 d of Buchinger fasting.* J Nutr Sci, 2019. **8**: p. e36.
41. Magne, F., et al., *The Firmicutes/Bacteroidetes Ratio: A Relevant Marker of Gut Dysbiosis in Obese Patients?* Nutrients, 2020. **12**(5).
42. Purchiaroni, F., et al., *The role of intestinal microbiota and the immune system.* Eur Rev Med Pharmacol Sci, 2013. **17**(3): p. 323-33.
43. Leeming, E.R., et al., *Effect of Diet on the Gut Microbiota: Rethinking Intervention Duration.* Nutrients, 2019. **11**(12): p. 2862.
44. Heintz-Buschart, A., et al., *Integrated multi-omics of the human gut microbiome in a case study of familial type 1 diabetes.* Nature Microbiology, 2016. **2**(1): p. 16180.
45. Narayanasamy, S., et al., *IMP: a pipeline for reproducible reference-independent integrated metagenomic and metatranscriptomic analyses.* Genome Biology, 2016. **17**(1): p. 260.
46. Wilmes, P., A. Heintz-Buschart, and P.L. Bond, *A decade of metaproteomics: where we stand and what the future holds.* Proteomics, 2015. **15**(20): p. 3409-17.
47. Gabel, K., et al., *Effects of 8-hour time restricted feeding on body weight and metabolic disease risk factors in obese adults: A pilot study.* Nutr Healthy Aging, 2018. **4**(4): p. 345-353.
48. Hughes, A.J., et al., *A clinicopathologic study of 100 cases of Parkinson's disease.* Arch Neurol, 1993. **50**(2): p. 140-8.
49. Hoehn, M.M. and M.D. Yahr, *Parkinsonism.* onset, progression, and mortality, 1967. **17**(5): p. 427-427.
50. Kay, J. and K.S. Upchurch, *ACR/EULAR 2010 rheumatoid arthritis classification criteria.* Rheumatology (Oxford), 2012. **51 Suppl 6**: p. vi5-9.
51. Feng, X., et al., *Body Mass Index and the Risk of Rheumatoid Arthritis: An Updated Dose-Response Meta-Analysis.* Biomed Res Int, 2019. **2019**: p. 3579081.
52. Wells G, B.J., Teng J, et al, *Validation of the 28-joint Disease Activity Score (DAS28) and European League Against Rheumatism response criteria based on C-reactive protein against disease progression in patients with rheumatoid arthritis, and comparison with the DAS28 based on erythrocyte sedimentation rate.* Annals of the Rheumatic Diseases 2009. **68**: p. 954-960.
53. Trenkwalder, C., et al., *Parkinson's disease sleep scale--validation of the revised version PDSS-2.* Mov Disord, 2011. **26**(4): p. 644-52.
54. Bushnell, D.M. and M.L. Martin, *Quality of life and Parkinson's disease: translation and validation of the US Parkinson's Disease Questionnaire (PDQ-39).* Qual Life Res, 1999. **8**(4): p. 345-50.
55. Smolen, J.S., et al., *A simplified disease activity index for rheumatoid arthritis for use in clinical practice.* Rheumatology (Oxford), 2003. **42**(2): p. 244-57.
56. Raspe, H.H., Hagedorn, U., Kohlmann, T., & Mattussek, S., *Der Funktionsfragebogen Hannover (FFbH): Ein Instrument zur Funktionsdiagnostik bei polyartikulären Gelenkerkrankungen*, in *Ergebnisse sozialwissenschaftlicher Evaluation eines Modellversuchs (pp. 164-182)*. 1990, Schattauer Verlag.

57. Goetz, C.G., et al., *Movement Disorder Society-sponsored revision of the Unified Parkinson's Disease Rating Scale (MDS-UPDRS): Process, format, and clinimetric testing plan.* Mov Disord, 2007. **22**(1): p. 41-7.

58. Chaudhuri, K.R., et al., *International multicenter pilot study of the first comprehensive self-completed nonmotor symptoms questionnaire for Parkinson's disease: the NMSQuest study.* Mov Disord, 2006. **21**(7): p. 916-23.

59. Chaudhuri, K.R., et al., *The metric properties of a novel non-motor symptoms scale for Parkinson's disease: Results from an international pilot study.* Mov Disord, 2007. **22**(13): p. 1901-11.

60. Wolfe, F., *A brief clinical health assessment instrument clinhaq.* Arthritis and Rheumatism, 1989. **32**(4 Suppl): p. S99-S99.

61. Lewis, S.J. and K.W. Heaton, *Stool form scale as a useful guide to intestinal transit time.* Scand J Gastroenterol, 1997. **32**(9): p. 920-4.

62. Topp, C.W., et al., *The WHO-5 Well-Being Index: a systematic review of the literature.* Psychother Psychosom, 2015. **84**(3): p. 167-76.

63. Zigmond, A.S. and R.P. Snaith, *The hospital anxiety and depression scale.* Acta Psychiatr Scand, 1983. **67**(6): p. 361-70.

64. McNair DM, L.M., Droppleman LF, *Edits Manual for the Profile of Mood States (Poms). Rev ed.* . San Diego: Educational and Industrial Testing Service, 1992.

65. Harris, P.A., et al., *Research electronic data capture (REDCap)--a metadata-driven methodology and workflow process for providing translational research informatics support.* J Biomed Inform, 2009. **42**(2): p. 377-81.

66. Wilmes, P., Roume, H., Hiller, K. & Cordes, T., *Method and kit for the isolation of genomic DNA, RNA, proteins and metabolites from a single biological sample.*, in *World Intellectual Property Organization*, C.D.R.P.-G.L. Université Du Luxembourg, Editor. 2014: Switzerland.

67. Roume, H., et al., *A biomolecular isolation framework for eco-systems biology.* The ISME Journal, 2013. **7**(1): p. 110-121.

68. Locati, M.D., et al., *Improving small RNA-seq by using a synthetic spike-in set for size-range quality control together with a set for data normalization.* Nucleic Acids Res, 2015. **43**(14): p. e89.

69. Wampach, L., et al., *Birth mode is associated with earliest strain-conferred gut microbiome functions and immunostimulatory potential.* Nature Communications, 2018. **9**(1): p. 5091.

70. Albanese, D. and C. Donati, *Strain profiling and epidemiology of bacterial species from metagenomic sequencing.* Nature Communications, 2017. **8**(1): p. 2260.

71. Vandeputte, D., et al., *Quantitative microbiome profiling links gut community variation to microbial load.* Nature, 2017. **551**(7681): p. 507-511.

72. Tang, H., S. Li, and Y. Ye, *A Graph-Centric Approach for Metagenome-Guided Peptide and Protein Identification in Metaproteomics.* PLoS Comput Biol, 2016. **12**(12): p. e1005224.

73. Tabb, D.L., C.G. Fernando, and M.C. Chambers, *MyriMatch: highly accurate tandem mass spectral peptide identification by multivariate hypergeometric analysis.* J Proteome Res, 2007. **6**(2): p. 654-61.

74. Heintz-Buschart, A., et al., *The nasal and gut microbiome in Parkinson's disease and idiopathic rapid eye movement sleep behavior disorder.* Mov Disord, 2018. **33**(1): p. 88-98.

For peer review only

75. Chen, S.G., et al., *Exposure to the Functional Bacterial Amyloid Protein Curli Enhances Alpha-Synuclein Aggregation in Aged Fischer 344 Rats and Caenorhabditis elegans.* Scientific Reports, 2016. **6**(1): p. 34477.
76. Wilmes, P., et al., *Metabolome-proteome differentiation coupled to microbial divergence.* mBio, 2010. **1**(5).
77. Kim, D.H., et al., *LC-MS-based absolute metabolite quantification: application to metabolic flux measurement in trypanosomes.* Metabolomics, 2015. **11**(6): p. 1721-1732.
78. Lei, Z., D.V. Huhman, and L.W. Sumner, *Mass spectrometry strategies in metabolomics.* J Biol Chem, 2011. **286**(29): p. 25435-42.
79. *Immunophenotyping assessment in a COVID-19 cohort (IMPACC): A prospective longitudinal study.* Sci Immunol, 2021. **6**(62).
80. Shah, P., et al., *A microfluidics-based in vitro model of the gastrointestinal human–microbe interface.* Nature Communications, 2016. **7**(1): p. 11535.
81. Aho, V.T.E., et al., *SnapShot: The Expobiome Map.* Cell Host Microbe, 2022. **30**(9): p. 1340-1340.e1.
82. Tusher, V.G., R. Tibshirani, and G. Chu, *Significance analysis of microarrays applied to the ionizing radiation response.* Proc Natl Acad Sci U S A, 2001. **98**(9): p. 5116-21.
83. Finnell, J.S., et al., *Is fasting safe? A chart review of adverse events during medically supervised, water-only fasting.* BMC Complementary and Alternative Medicine, 2018. **18**(1): p. 67.
84. Chan, A.-W., et al., *SPIRIT 2013 explanation and elaboration: guidance for protocols of clinical trials.* BMJ : British Medical Journal, 2013. **346**: p. e7586.

Figure Legends

Figure 1. Study design. This figure illustrates the study design with five different arms in total, two of which continue with the longitudinal part of the study. Visits take place at the clinical sites at each timepoint and include the collection of the displayed samples. This image was generated using Biorender software (http://www.biorender.com). T, timepoint; W, week; D, day; M, month.

For peer review only

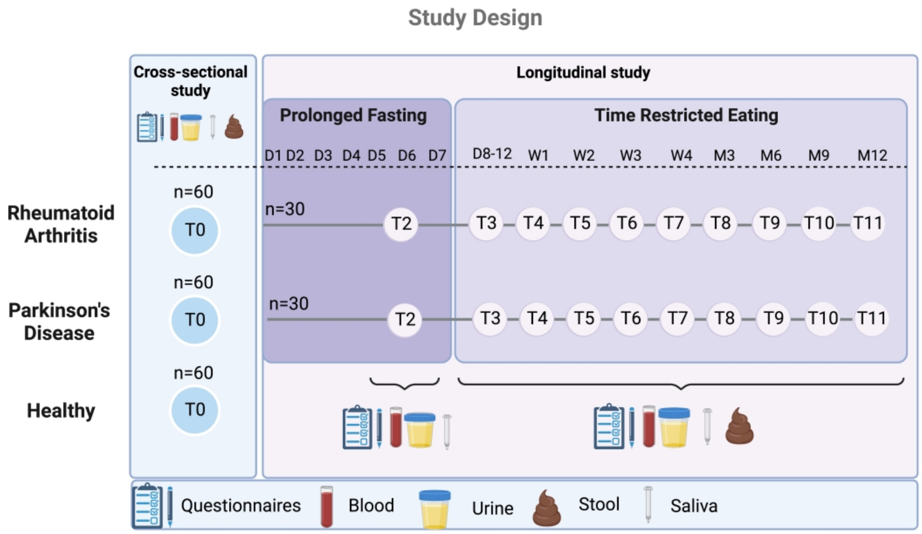

Figure 1. Study design. This figure illustrates the study design with five different arms in total, two of which continue with the longitudinal part of the study. Visits take place at the clinical sites at each timepoint and include the collection of the displayed samples. This image was generated using Biorender software (http://www.biorender.com). T, timepoint; W, week; D, day; M, month.

279x177mm (600 x 600 DPI)

Reporting checklist for protocol of a clinical trial.

Based on the SPIRIT guidelines.

Instructions to authors

Complete this checklist by entering the page numbers from your manuscript where readers will find each of the items listed below.

Your article may not currently address all the items on the checklist. Please modify your text to include the missing information. If you are certain that an item does not apply, please write "n/a" and provide a short explanation.

Upload your completed checklist as an extra file when you submit to a journal.

In your methods section, say that you used the SPIRITreporting guidelines, and cite them as:

Chan A-W, Tetzlaff JM, Gøtzsche PC, Altman DG, Mann H, Berlin J, Dickersin K, Hróbjartsson A, Schulz KF, Parulekar WR, Krleža-Jerić K, Laupacis A, Moher D. SPIRIT 2013 Explanation and Elaboration: Guidance for protocols of clinical trials. BMJ. 2013;346:e7586

		Reporting Item	Page Number
**Administrative information**			
Title	#1	Descriptive title identifying the study design, population, interventions, and, if applicable, trial acronym	1
Trial registration	#2a	Trial identifier and registry name. If not yet registered, name of intended registry	2
Trial registration: data set	#2b	All items from the World Health Organization Trial Registration Data Set	n/a
Protocol version	#3	Date and version identifier	n/a
Funding	#4	Sources and types of financial, material, and other support	13
Roles and responsibilities: contributorship	#5a	Names, affiliations, and roles of protocol contributors	1

For peer review only

Roles and responsibilities: sponsor contact information	#5b	Name and contact information for the trial sponsor	1
Roles and responsibilities: sponsor and funder	#5c	Role of study sponsor and funders, if any, in study design; collection, management, analysis, and interpretation of data; writing of the report; and the decision to submit the report for publication, including whether they will have ultimate authority over any of these activities	1, 13
Roles and responsibilities: committees	#5d	Composition, roles, and responsibilities of the coordinating centre, steering committee, endpoint adjudication committee, data management team, and other individuals or groups overseeing the trial, if applicable (see Item 21a for data monitoring committee)	1, 13
**Introduction**			
Background and rationale	#6a	Description of research question and justification for undertaking the trial, including summary of relevant studies (published and unpublished) examining benefits and harms for each intervention	5
Background and rationale: choice of comparators	#6b	Explanation for choice of comparators	5
Objectives	#7	Specific objectives or hypotheses	5
Trial design	#8	Description of trial design including type of trial (eg, parallel group, crossover, factorial, single group), allocation ratio, and framework (eg, superiority, equivalence, non-inferiority, exploratory)	5
**Methods: Participants, interventions, and outcomes**			
Study setting	#9	Description of study settings (eg, community clinic, academic hospital) and list of countries where data will be collected. Reference to where list of study sites can be obtained	6

For peer review only

Eligibility criteria	#10	Inclusion and exclusion criteria for participants. If applicable, eligibility criteria for study centres and individuals who will perform the interventions (eg, surgeons, psychotherapists)	6
Interventions: description	#11a	Interventions for each group with sufficient detail to allow replication, including how and when they will be administered	5,7
Interventions: modifications	#11b	Criteria for discontinuing or modifying allocated interventions for a given trial participant (eg, drug dose change in response to harms, participant request, or improving / worsening disease)	5,7
Interventions: adherance	#11c	Strategies to improve adherence to intervention protocols, and any procedures for monitoring adherence (eg, drug tablet return; laboratory tests)	7
Interventions: concomitant care	#11d	Relevant concomitant care and interventions that are permitted or prohibited during the trial	6,7
Outcomes	#12	Primary, secondary, and other outcomes, including the specific measurement variable (eg, systolic blood pressure), analysis metric (eg, change from baseline, final value, time to event), method of aggregation (eg, median, proportion), and time point for each outcome. Explanation of the clinical relevance of chosen efficacy and harm outcomes is strongly recommended	10f
Participant timeline	#13	Time schedule of enrolment, interventions (including any run-ins and washouts), assessments, and visits for participants. A schematic diagram is highly recommended (see Figure)	5
Sample size	#14	Estimated number of participants needed to achieve study objectives and how it was determined, including clinical and statistical assumptions supporting any sample size calculations	12
Recruitment	#15	Strategies for achieving adequate participant enrolment to reach target sample size	5
**Methods: Assignment of interventions (for controlled trials)**			
Allocation: sequence generation	#16a	Method of generating the allocation sequence (eg, computer-generated random numbers), and list of any factors for stratification. To reduce predictability of a random sequence, details of any planned restriction (eg, blocking) should be	n/a

For peer review only

		provided in a separate document that is unavailable to those who enrol participants or assign interventions	
Allocation concealment mechanism	#16b	Mechanism of implementing the allocation sequence (eg, central telephone; sequentially numbered, opaque, sealed envelopes), describing any steps to conceal the sequence until interventions are assigned	n/a
Allocation: implementation	#16c	Who will generate the allocation sequence, who will enrol participants, and who will assign participants to interventions	n/a
Blinding (masking)	#17a	Who will be blinded after assignment to interventions (eg, trial participants, care providers, outcome assessors, data analysts), and how	n/a
Blinding (masking): emergency unblinding	#17b	If blinded, circumstances under which unblinding is permissible, and procedure for revealing a participant's allocated intervention during the trial	n/a
**Methods: Data collection, management, and analysis**			
Data collection plan	#18a	Plans for assessment and collection of outcome, baseline, and other trial data, including any related processes to promote data quality (eg, duplicate measurements, training of assessors) and a description of study instruments (eg, questionnaires, laboratory tests) along with their reliability and validity, if known. Reference to where data collection forms can be found, if not in the protocol	10f
Data collection plan: retention	#18b	Plans to promote participant retention and complete follow-up, including list of any outcome data to be collected for participants who discontinue or deviate from intervention protocols	7,12
Data management	#19	Plans for data entry, coding, security, and storage, including any related processes to promote data quality (eg, double data entry; range checks for data values). Reference to where details of data management procedures can be found, if not in the protocol	12
Statistics: outcomes	#20a	Statistical methods for analysing primary and secondary outcomes. Reference to where other details of the statistical analysis plan can be found, if not in the protocol	12f

For peer review only

Statistics: additional analyses	#20b	Methods for any additional analyses (eg, subgroup and adjusted analyses)	12f
Statistics: analysis population and missing data	#20c	Definition of analysis population relating to protocol non-adherence (eg, as randomised analysis), and any statistical methods to handle missing data (eg, multiple imputation)	12f
**Methods: Monitoring**			
Data monitoring: formal committee	#21a	Composition of data monitoring committee (DMC); summary of its role and reporting structure; statement of whether it is independent from the sponsor and competing interests; and reference to where further details about its charter can be found, if not in the protocol. Alternatively, an explanation of why a DMC is not needed	12f
Data monitoring: interim analysis	#21b	Description of any interim analyses and stopping guidelines, including who will have access to these interim results and make the final decision to terminate the trial	n/a
Harms	#22	Plans for collecting, assessing, reporting, and managing solicited and spontaneously reported adverse events and other unintended effects of trial interventions or trial conduct	12
Auditing	#23	Frequency and procedures for auditing trial conduct, if any, and whether the process will be independent from investigators and the sponsor	n/a
**Ethics and dissemination**			
Research ethics approval	#24	Plans for seeking research ethics committee / institutional review board (REC / IRB) approval	2
Protocol amendments	#25	Plans for communicating important protocol modifications (eg, changes to eligibility criteria, outcomes, analyses) to relevant parties (eg, investigators, REC / IRBs, trial participants, trial registries, journals, regulators)	2, 13
Consent or assent	#26a	Who will obtain informed consent or assent from potential trial participants or authorised surrogates, and how (see Item 32)	6

For peer review only

Consent or assent: ancillary studies	#26b	Additional consent provisions for collection and use of participant data and biological specimens in ancillary studies, if applicable	6
Confidentiality	#27	How personal information about potential and enrolled participants will be collected, shared, and maintained in order to protect confidentiality before, during, and after the trial	12
Declaration of interests	#28	Financial and other competing interests for principal investigators for the overall trial and each study site	13
Data access	#29	Statement of who will have access to the final trial dataset, and disclosure of contractual agreements that limit such access for investigators	12f
Ancillary and post trial care	#30	Provisions, if any, for ancillary and post-trial care, and for compensation to those who suffer harm from trial participation	n/a
Dissemination policy: trial results	#31a	Plans for investigators and sponsor to communicate trial results to participants, healthcare professionals, the public, and other relevant groups (eg, via publication, reporting in results databases, or other data sharing arrangements), including any publication restrictions	2, 13
Dissemination policy: authorship	#31b	Authorship eligibility guidelines and any intended use of professional writers	13
Dissemination policy: reproducible research	#31c	Plans, if any, for granting public access to the full protocol, participant-level dataset, and statistical code	n/a
**Appendices**			
Informed consent materials	#32	Model consent form and other related documentation given to participants and authorised surrogatess	6
Biological specimens	#33	Plans for collection, laboratory evaluation, and storage of biological specimens for genetic or molecular analysis in the current trial and for future use in ancillary studies, if applicable	7

The SPIRIT Explanation and Elaboration paper is distributed under the terms of the Creative Commons Attribution License CC-BY-NC. This checklist was completed on 07. November 2022 using https://www.goodreports.org/, a tool made by the EQUATOR Network in collaboration with Penelope.ai

For peer review only

BMJ Open

Protocol for a multicentre cross-sectional, longitudinal ambulatory clinical trial in rheumatoid arthritis and Parkinson's disease patients analysing the relation between the gut microbiome, fasting and immune status in Germany (ExpoBiome)

Journal:	*BMJ Open*
Manuscript ID	bmjopen-2022-071380.R3
Article Type:	Protocol
Date Submitted by the Author:	14-Jul-2023
Complete List of Authors:	Hansen, Bérénice; LCSB<br>Laczny, Cédric C.; LCSB<br>Aho, Velma T.E.; LCSB<br>Frachet-Bour, Audrey; LCSB<br>Habier, Janine; LCSB<br>Ostaszewski, Marek; LCSB<br>Michalsen, Andreas; Charité Universitätsmedizin Berlin, Institute for Social Medicine, Epidemiology and Health Economics; Immanuel Hospital Berlin-Wannsee Branch, Department of Internal and Integrative Medicine<br>Hanslian, Etienne; Charite Universitatsmedizin Berlin, Institute for Social Medicine, Epidemiology and Health Economics; Immanuel Hospital Berlin-Wannsee Branch<br>Koppold-Liebscher, Daniela; Charité Universitätsmedizin Berlin, Institute for Social Medicine, Epidemiology and Health Economics; Immanuel Hospital Berlin-Wannsee Branch<br>Hartmann, Anika; Charité Universitätsmedizin Berlin, Institute of Social Medicine, Epidemiology and Health Economics; Charité Universitätsmedizin Berlin, Department of Dermatology, Venereology and Allergology<br>Steckhan, Nico; Charité Universitätsmedizin Berlin, Institute for Social Medicine, Epidemiology and Health Economics; University of Potsdam, Digital Health - Connected Healthcare, Hasso Plattner Institute<br>Mollenhauer, Brit; University Medical Center Göttingen; Paracelsus-Kliniken Deutschland GmbH<br>Schade, Sebastian; University Medical Center Göttingen; Paracelsus-Kliniken Deutschland GmbH,<br>Roomp, Kirsten; LCSB<br>Schneider, Jochen; LCSB; Saarland University Hospital and Saarland University Faculty of Medicine, Department of Internal Medicine and Psychiatry<br>Wilmes, Paul; LCSB; University of Luxembourg, Department of Life Sciences and Medicine
**Primary Subject Heading**:	Nutrition and metabolism
Secondary Subject Heading:	Immunology (including allergy), Rheumatology, Pharmacology and

For peer review only - http://bmjopen.bmj.com/site/about/guidelines.xhtml

	therapeutics, Neurology, Evidence based practice
Keywords:	IMMUNOLOGY, Rheumatology < INTERNAL MEDICINE, MICROBIOLOGY, Parkinson-s disease < NEUROLOGY, NUTRITION & DIETETICS, Clinical trials < THERAPEUTICS

SCHOLARONE™
Manuscripts

Protocol for a multicentre cross-sectional, longitudinal ambulatory clinical trial in rheumatoid arthritis and Parkinson's disease patients analysing the relation between the gut microbiome, fasting and immune status in Germany (ExpoBiome)

Bérénice Hansen[1], Cédric C. Laczny[1], Velma T.E. Aho[1], Audrey Frachet-Bour[1], Janine Habier[1], Marek Ostaszewski[1], Andreas Michalsen[4,5], Etienne Hanslian[4,5], Daniela A. Koppold[4,5,8], Anika Hartmann[4,10], Nico Steckhan[4,9], Brit Mollenhauer[6,7], Sebastian Schade[6,7], Kirsten Roomp[1], Jochen G. Schneider[1,3+], Paul Wilmes[1,2+]

[1]Luxembourg Centre for Systems Biomedicine, University of Luxembourg, Campus Belval, 7, avenue des Hauts-Fourneaux, L-4362 Esch-sur-Alzette, Luxembourg

[2]Department of Life Sciences and Medicine, University of Luxembourg, 7, avenue des Hauts-Fourneaux, L-4362 Esch-sur-Alzette, Luxembourg

[3]Department of Internal Medicine and Psychiatry, Saarland University Medical Center, D- 66421 Homburg Saar, Germany

[4]Institute for Social Medicine, Epidemiology and Health Economics, Charité – Universitätsmedizin Berlin, corporate member of Freie Universität Berlin, Humboldt-Universität zu Berlin, and Berlin Institute of Health

[5]Department of Internal and Integrative Medicine, Immanuel Hospital Berlin, Germany

[6]Paracelsus-Elena-Klinik, Kassel, Germany

[7]University Medical Center Göttingen, Germany

[8]Department of Pediatrics, Division of Oncology and Hematology, Charité – Universitätsmedizin Berlin, Corporate Member of Freie Universität Berlin, Humboldt-Universität zu Berlin and Berlin Institute of Health, Berlin, Germany

[9]Digital Health - Connected Healthcare, Hasso Plattner Institute, University of Potsdam, Potsdam, Germany

[10]Department of Dermatology, Venereology and Allergology, Charité—Universitätsmedizin Berlin, Corporate Member of Freie Universität Berlin and Humboldt-Universität zu Berlin, Berlin, Germany

[+]contributed equally

*Correspondence to:

Jochen Schneider (jochen.schneider@uni.lu)
Paul Wilmes (paul.wilmes@uni.lu)

Luxembourg Centre for Systems Biomedicine,
University of Luxembourg, Campus Belval,
7, avenue des Hauts-Fourneaux,
L-4362 Esch-sur-Alzette, Luxembourg

Word count: 4461

For peer review only

Abstract

Introduction

Chronic inflammatory diseases like rheumatoid arthritis (RA) and neurodegenerative disorders like Parkinson's disease (PD) have recently been associated with a decreased diversity in the gut microbiome, emerging as key driver of various diseases. The specific interactions between gut-borne microorganisms and host pathophysiology remain largely unclear. The microbiome can be modulated by interventions comprising nutrition.

The aim of our clinical study is to (1) examine effects of prolonged fasting and time-restricted eating (TRE) on the outcome parameters and the immunophenotypes of RA and PD with (2) special consideration of microbial taxa and molecules associated with changes expected in (1), and (3) identify factors impacting the disease course and treatment by in-depth screening of microorganisms and molecules in personalised HuMiX gut-on-chip models, to identify novel targets for anti-inflammatory therapy.

Methods and Analysis

This trial is an open-label, multicentre, controlled clinical trial consisting of a cross-sectional and a longitudinal study. A total of 180 patients is recruited. For the cross-sectional study, 60 patients with PD, 60 patients with RA and 60 healthy controls are recruited at two different, specialized clinical sites. For the longitudinal part, 30 patients with PD and 30 patients with RA undergo 5-7 days of prolonged fasting (PF) followed by TRE (16:8) for a period of 12 months. One baseline visit takes place before the PF intervention and 10 follow-up visits will follow over a period of 12 months (April 2021 to November 2023).

Ethics and dissemination

Ethical approval was obtained to plan and conduct the trial from the institutional review board of the Charité-Universitätsmedizin Berlin (EA1/204/19), the ethics committee of the state medical association (Landesärztekammer) of Hessen (2021-2230-zvBO) and the Ethics Review Panel (ERP) of the University of Luxembourg (ERP 21-001-A ExpoBiome). The results of this study will be disseminated through peer-reviewed publications, scientific presentations and social media.

Trial registration number at clinicaltrials.gov:

NCT04847011

Key words: Microbiome, fasting therapy, intermittent fasting, time restricted eating, chronic disease, rheumatoid arthritis, Parkinson's disease, nutrition, chronic diseases, ExpoBiome, inflammation, gut on a chip, HuMiX, immunophenotype, metagenomics, metatranscriptomics, metaproteomics, metabolomics

Strengths and limitations of the study

- The participants of the longitudinal study will be closely monitored for 12 months and routine blood parameters as well as anthropometric data and questionnaires will be precisely documented.

For peer review only

- This study will identify novel microbiome-derived common and disease-associated molecules involved in immune system modulation in two major chronic diseases: RA and PD.
- This study aims at also identifying novel targeted pathways to control chronic inflammatory conditions in the future.
- A limitation is the heterogeneity of the cohorts regarding age and sex, which is due to the prevalence of the diseases: RA is more common in women, while PD is more common in men and has a later disease onset.
- A bias exists in choosing RA and PD as chronic disorders to study immunophenotypes although generalisable results are targeted.

Introduction (1339)

The human microbiome is emerging as a key driver of various diseases through its complex of distinct yet connected biomolecules (referred to as the "*expobiome*")[1, 2]. The expobiome comprises a diverse set of nucleic acids, polypeptides and metabolites which, in the gut alone, are present in substantial concentrations[1]. However, the specific interactions between gut-borne microorganisms and host (patho)physiology remain largely unknown. Although host genetics shape the composition of the gut microbiome, the latter is particularly influenced by non-genetic factors such as lifestyle and diet[3, 4]. Therefore, the microbiome is a plausible target to modify health outcomes.

Individuals suffering from chronic diseases, including autoimmune, metabolic, and neurodegenerative diseases as well as cancer, often present alterations in their gut microbiome composition. These shifts are typically characterised by an overgrowth of one or several microbial species with likely adverse effects as well as a decrease in beneficial taxa[5]. Such imbalances are referred to as dysbiosis. Although structural microbiome changes are clearly detectable, the mechanistic or functional consequences of dysbiosis are still largely unknown. However, they may result in dysregulated interactions with the immune system[6]. Considering the intricacy of the immune system, the question arises whether the observed microbiome changes are cause or consequence of disease. This implies that, in addition to the genetic predisposition of the host, the gut microbiome needs to be considered a potential pathogenic factor or major driver of disease onset and course[3, 4].

RA and PD are two specific examples representing dysregulated microbiome-immune system interactions [7, 8]. RA is a multifactorial, chronic, and systemic autoimmune disease, primarily affecting the lining of the synovial joints with a higher risk and younger age for disease onset in women and a global prevalence of 1%[9, 10]. The exact disease pathogenesis is still unclear and no cure for RA currently exists. In addition to the common local articular symptoms of RA, systemic comorbidities can affect the vasculature, metabolism and bones[11]. Besides various environmental risk factors e.g. smoking and a Western diet, the host microbiome is associated with the pathophysiology of the disease[12]. The diversity of the gut microbiome has been reported to be decreased in individuals with RA, compared with the general population, and is correlated with disease duration, activity, and autoantibody levels[13, 14]. Studies in murine models also report that autoimmune arthritis is strongly attenuated under germ-free conditions[15]. The introduction of specific bacteria, e.g. segmented filamentous bacteria, into germ-free animals or oral infection with *Porphyromonas gingivalis* drive autoimmune arthritis through activation of T helper cells[15]. Several different taxa, including *Prevotella copri*, *Lactobacillus* spp. and *Colinsella* spp. are enriched in the gut microbiome of patients with RA and correlate positively with disease markers e.g. immunoglobulins IgA and IgG , while other taxa like *Haemophilus* spp. and *Faecalibacterium* spp. are typically found at lower abundances in

For peer review only

patients with RA compared to healthy individuals[13, 16, 17]. Alterations of the gut microbiome may therefore have an important impact on RA pathophysiology[12].

PD affects 0.4-2% of the population over 65 years worldwide and is the second most common progressive neurodegenerative disease with men being 1.5 times more likely to be affected than women[18]. Cardinal symptoms are motor deficiencies such as tremor and rigidity, but also include a wide range of non-motor symptoms, such as hyposmia, depression, insomnia or cognitive impairment, severely impacting patients' quality of life[19]. Aggregations of the protein α-synuclein in the dopaminergic substantia nigra represent the main neuropathological manifestations[20]. PD-associated loss of dopaminergic neurons involves mechanisms of inflammatory and autoimmune responses with microglial activity as a major driver [21].Dysbiosis of the gut microbiome has been associated with the characteristic motor deficits and pathophysiological changes in the enteric and central nervous systems in animal studies. Increased relative abundances of the genera *Akkermansia, Bifidobacterium, Lactobacillus,* and *Methanobrevibacter* and decreased abundances in *Faecalibacterium* and *Roseburia* have been reported[22, 23]. Two recently published clinical-trials with prebiotic supplementation in PD observed a shift in gut microbiome composition, an increase in short-chain fatty acids (SCFA) and a reduction in non-motor-symptoms [24, 25]. Most patients with PD suffer from gastrointestinal symptoms such as constipation and irritable bowel syndrome (IBS) -like symptoms[26]. The gut-brain axis, e.g. by-products produced by the gut microbiome, may contribute to the production of α-synuclein aggregates in the enteric nervous system[27]. In addition, increased intestinal permeability[28] as driver for enteric inflammation occur in PD and substantiate a role of peripheral inflammation in the initiation and the progression of the disease[29].

One factor with known major impact on the gut microbiome and on chronic diseases is diet[7]. Dietary approaches as fasting have already been used by Hippocrates in the 5th century BCE and have been applied ever since by numerous medical schools to treat acute and chronic diseases [30-32]. Various practices of caloric restriction through fasting have repeatedly shown remarkable health benefits[33, 34]. Maifeld *et al.* found that a 5-day fast followed by a modified Dietary Approach to Stop Hypertension (DASH), with additional emphasis on plant-based and Mediterranean diets, reduced systolic blood pressure, BMI, and the need for antihypertensive medications at three months post intervention compared with DASH alone [35].

Furthermore, Choi *et al.* demonstrated that cycles of a fasting-mimicking diet suppress autoimmunity and stimulate remyelination via oligodendrocyte regeneration in a murine experimental autoimmune encephalomyelitis (EAE) model[36]. Jordan *et al.* described a reduction in monocyte metabolic and inflammatory activity after a short-term fast and conclude that fasting attenuates chronic inflammatory diseases without compromising monocyte capacity for mobilisation during acute infectious inflammation and tissue repair[37].

These improvements can, however, typically only be maintained for a limited period of time, and the symptoms can reappear after reintroduction of the patients' standard diet. Hence, protocols to sustain these beneficial effects are of utmost importance. In mouse models of PD, intermittent fasting (IF) has led to several improvements including decreased excitotoxicity, reduced neurodegeneration and protection against autonomic dysfunction, motor and cognitive decline[38].

IF and PF may have potent immunomodulatory effects which may partially be mediated by the gut microbiome and the fasting induced alterations of the latter[39]. These microbial shifts include upregulation of *Akkermansia muciniphila*, *Bacteroides fragilis*, other *Bacteroides* spp., Proteobacteria, and butyric acid producing *Lachnospiraceae,* but also *Odoribacter*, which is negatively associated with blood pressure[35, 40]. Interestingly, an overall decrease of the Firmicutes/Bacteroidetes ratio could be observed, a high ratio is commonly associated with several pathologies, including RA [41].

For peer review only

A potential mechanism underlying the observed beneficial effects induced by dietary interventions might be a direct gut microbiome-immune system interaction by pattern recognition. The microbiome can regulate the intestinal innate immune system by modulating toll-like receptor (TLR) expression on immunosensor cell surface through microbe-associated molecular patterns (MAMPs), which can consequently trigger cytokine production and up-regulation of molecules on antigen presenting cells, leading to activation of T cells[42]. Therefore, a change in gut microbiome composition can lead to different outcomes in immune signalling pathways and either favour or suppress inflammation and autoimmunity.

The impact and importance of the gut microbiome on human physiology and its potential modifications by nutrition and dietary patterns, have been underestimated for centuries[43]. Reasons may include missing standardised therapeutic protocols, the interindividual variability in the response to fasting, lack of knowledge about possible adverse effects, and difficulties in the interpretation of underlying mechanisms seen in clinical trials, but also in the comparably low potential for achieving economic revenue or scientific impact[8].

Modern experimental approaches and computational integration allow a multi-layer analysis of digestive processes in low caloric settings including the gut microbiome[44]. These technological developments also permit a closer investigation of the link between the immune system and severe caloric restriction.

To our knowledge no clinical trials have been investigating the connection between IF or PF and PD in humans so far[38]. Our study aims to elucidate the causal relationship between the gut microbiome and the immune system. To do so, we will use analyses of the molecular basis of human-microbiome interactions enabled by high throughput methodologies such as the combination of metagenomics, metatranscriptomics and metaproteomics. Moreover, we are aiming at identifying new genes, proteins, metabolites, and host pathways facilitating the development of novel diagnostic and therapeutic tools[45, 46].

Methods and Analysis (3122)

Study objectives

The first objective of the study is to define specific gut microbiome-derived molecules in RA and PD, compared to healthy individuals, and relate this information to the immunophenotypes of the individuals. The second objective is to identify and track common and disease-specific molecular signatures to predict the outcome of a gut microbiome-targeted therapeutic intervention, here fasting, on inflammation-driven symptoms in RA and PD. The third objective of the study is to identify and validate microbiome-derived effector molecules which downregulate pro-inflammatory innate and adaptive immune pathways.

Study design

The ExpoBiome cohort consists of 180 adult individuals, meeting the exclusion and inclusion criteria (Table 1), for the cross-sectional study (objectives 1 and 3) and 60 adult individuals for the longitudinal study (objectives 2 and 3). There are five different arms in total: (1) RA – cross-sectional arm [60 patients], (2) PD – cross-sectional arm [60 patients], and (3) healthy controls – cross-sectional arm [60 patients], (4) RA – longitudinal arm [30 patients], (5) PD – longitudinal arm [30 patients] (Figure1).

For peer review only

At the first visit (T0), patients answer several questionnaires, and blood, urine, saliva, and stool samples are obtained (Table 2).
The longitudinal arms (4) and (5) undergo a 5–7-day PF with a dietary energy supply of max. 350-400 kcal per day with vegetable or grain broths as well as fresh vegetable juices[31, 40]. After the PF, the longitudinal arms follow a dietary regimen including the concept of TRE for a period of 12 months following the 16:8 pattern[47]. This means that food intake is allowed ad libitum for 8 h, followed by 16 h of fasting where no food should be consumed. The intake of non-caloric beverages, e.g., water, unsweetened tea or coffee is, however, allowed. The participants attend one follow-up visit (T2) during the PF and 9 follow-up visits during the 12 months of TRE (Figure 1).

Patient and Public Involvement

Feedback of patients during former clinical trials at the study centre in Berlin was integrated in the planning and design of the fasting intervention of this study. Patients are not involved in the conduct, reporting, or dissemination plans of this research.

Recruitment and randomisation

Patients are recruited by the specialised sites via different sources, e.g., by direct referral from either a physician at the Immanuel Hospital Berlin and the outpatient department of the Institute of Social Medicine, Epidemiology and Health Economics at Charité-Universitätsmedizin Berlin, or the Paracelsus-Elena Clinic in Kassel, or by non-personal advertising strategies (e.g. flyers or social media).
For PD, the patients are screened by an experienced movement disorders specialist for featuring at least two of resting tremor, bradykinesia, and rigidity according to the United Kingdom Parkinson's Disease Society Brain Bank criteria[48]. Additionally, patients must show evidence of a dopaminergic deficit, either with DaTScan imaging or with a clear response to dopaminergic drugs. Motor and non-motor symptoms are assessed with the MDS-UPDRS (part I – IV) including the Hoehn and Yahr (severity) scale[49]. Additional PD-specific scales as Parkinson's Disease Sleep Scale-2, Parkinson's Disease Questionnaire-39, Non-Motor Symptoms Questionnaire and Non-Motor Symptoms Scale are used.
For patients with RA, the diagnosis has been made prior to the study by an experienced rheumatologist according to the European League Against Rheumatism (EULAR) criteria[50]. All clinical stages of RA will be included. We excluded patients with a BMI <18.5, as this indicates underweight, and fasting is not recommended. We did, however, not include an upper limit as fasting might be especially beneficial for patients with a BMI >24.9 and more than 60% of patients with RA are classified as overweight or obese[51]. For comorbidities we excluded mainly diseases which are known to interfere with the gut microbiome and might be potential confounders.
The chosen exclusion criteria will optimize the pairing process of healthy controls and patients with either RA or PD. However, as we have two diseases with different anthropometric characteristics (including age, gender, BMI) and only one control group, adding additional inclusion and exclusion criteria in the recruitment process would compromise on optimized matching. Furthermore, for the longitudinal part of the study, each patient will serve as his/her own control over time. Participants meeting all the inclusion and no exclusion criteria (Table 1) are assigned to their respective groups (RA, PD, or healthy control) (Figure 1) for the cross-sectional study after written informed consent.
Half of the patients from the RA group and half of the patients from the PD group is selected to take part in the longitudinal part of the study, including the fasting intervention according to their availability for all 11 visits and their willingness to follow TRE over 12 months. This study is an open-label trial, as blinding is not feasible in fasting interventions.

Table 1: Inclusion and exclusion criteria

Inclusion criteria	Exclusion criteria

For peer review only - http://bmjopen.bmj.com/site/about/guidelines.xhtml

• Age 18-79<br>• One of the following diagnoses: rheumatoid arthritis (first diagnosis >6 weeks ago), Parkinson's disease OR healthy volunteer<br>• Control ("healthy") individuals must be without any evidence of active known or treated RA, without any evidence of active, known or treated central nervous system disease, and without a known family history of idiopathic PD<br>• Control individuals should match the RA or PD individuals as closely as possible (sex, age, education)<br>• Present written declaration of consent<br>• Ability to understand the patient information and willingness to sign the consent form<br>• Consent to specimen collection and specimen use	• Gout or proven bacterial arthritis<br>• Participation in another study<br>• Existing/current eating disorder (bulimia nervosa, anorexia nervosa) within the past 5 years<br>• Severe internal disease (e.g. kidney deficiency with creatinine > 2mg/dl)<br>• Existing vegan diet or fasting during the last six months<br>• Presence or suspicion of atypical PD (e.g. early dementia, early autonomous dysfunction)<br>• Diagnosis of chronic inflammatory bowel diseases, celiac disease or colorectal cancer according to the guidelines of the German Society of Gastroenterology<br>• Use of anti-psychotic drugs<br>• Antibiotic use during the previous 12 months<br>• Start of novel therapy with disease-modifying anti-rheumatic drugs<br>• Pregnancy or breastfeeding women<br>• Contraindication for additional blood draws (e.g. haemoglobin <10)<br>• BMI < 18.5<br>• Psychiatric illness that limits understanding of the examination protocol (unable to consent)

Fasting dietary counselling

The fasting group is closely monitored by nutritionists trained in fasting therapy, backed up by physicians experienced in fasting, from the Charité – Universitätsmedizin Berlin and the Paracelsus-Elena Clinic to ensure a uniform implementation of the fasting guidelines and the well-being of the study participants. The monitoring consists of several in person and virtual meetings which held individually or in group settings. Five meetings including the visits T0 and T2 during the fasting week as well as a group meeting after PF to ensure a well-managed start to the TRE phase take place. Group sessions are standardised using a pre-set deck of slides to be discussed during the group meetings with only minor disease-related differences between the PD and RA groups. All longitudinal participants receive a study-specific script with information on fasting procedures. Although the adherence of the patients cannot be profoundly controlled in the ambulatory setting, the blood samples will allow us to have additional insight into the nutritional habits as well as the fasting state of the patients on the day of the visit (blood glucose levels).

For peer review only

Medication

The medical treatments of the patients are monitored and documented with every clinical visit. The fasting intervention might necessitate temporary adjustments of several medications e.g., anti-diabetic and anti-hypertensive drugs as insulin levels and hypertension will be reduced due to lack of food intake [31].

Data collection

Sample and data collection is performed at the two clinical sites, Charité – Universitätsmedizin Berlin and Paracelsus-Elena Clinic (Table 2).

Table 2: Sampling procedures.

a) Biochemical samples and procedures

Blood (123 mL at T0, 23 mL at T2-T11)
Stool collection (2 mL at T0 and T3-T11)
Saliva collection (3.5 mL at T0-T11)
Midstream urine (50 mL at T0 -T11)

b) Questionnaires

Disease specific
PD:<br>• Disease Activity Score (DAS-28) [52]<br>• Parkinson's Disease Sleep Scale-2 (PDSS-2) [53]<br>• Parkinson's Disease Questionnaire-39 (PDQ-39)[54]<br>• Simplified Disease Index Score (SDAI) [55]<br>• Funktionsfragebogen Hannover (FFbH-R) [56]<br>• Movement Disorder Society Unified PD Rating Scale (MDS-UPDRS)[57]<br>• Non-Motor Symptoms Questionnaire (NMSQ)[58]<br>• Non-Motor Symptoms Scale (NMSS)[59]<br>RA:<br>• Disease Activity Score (DAS-28) [55]

For peer review only

• Non-Motor Symptoms Questionnaire (NMSQ) [58]<br>• Funktionsfragebogen Hannover (FFbH-R) [56]
Dietary behaviour and lifestyle
• Fasting experience, expectation, and intervention<br>• Lifestyle<br>• 24H-Food-recall<br>• Food Frequency Questionnaire (FFQ)
General health and well-being
• Health Assessment Questionnaire (HAQ)[60]<br>• Bristol Stool Scale[61]<br>• Quality of Life questionnaire (WHO-5)[62]<br>• Hospital Anxiety and Depression Scale (HADS)[63]<br>• Profile of Mood States[64]

Anthropometric data and questionnaires

The electronic data capture system REDCap[65], a secure web-based application, is used to record all individual specific data. All data is stored on a secure server infrastructure at the host institution in Luxembourg. Weight, height, body mass index (BMI), heart rate and blood pressure in sitting and standing position as well as waist-hip-ratio is determined at every visit. Dietary behaviour, sociodemographic measurements (age, sex, education level, employment status, marital status), family history, current and previous illness and co-morbidities, and current medications, as well as disease-specific data, questionnaires about the well-being of the patients and data on the behavioural factors are collected at baseline, T6 (week 3), T9 (month 6) and T11 (month 12) (Table 2). Questionnaires (24h-Food Recall, Bristol Stool Scale) are answered at all visits by the study participants. Data storage, analysis and exchange are done only in pseudonymised fashion. The nutritional data is analysed using the Nutrilog 3.20 software (Nutrilog SAS, Marans).

Blood samples and parameters

Blood samples are collected at each visit, and immediately used for peripheral blood mononuclear cell (PBMC) isolation (T0), analysis by the study laboratory and centrifugation to freeze plasma samples at -80°C (T0-T11). A clinical standard laboratory report is generated after every visit for each study participant (Table 3). In addition to routine blood parameters, anti-citrullinated protein antibody

For peer review only

(ACPA), zonulin, fatty-acid binding protein 2 (FABP2), and calprotectin levels are measured. Aliquots are securely stored to account for novel observations and testing of hypotheses.

Table 3: Routine blood parameters measured at each timepoint (T0 for cross-sectional study, T0-T11 for longitudinal study)

**Haematology – EDTA-blood**	**Clinical Chemistry – Serum**
Basophils, %	Albumin
Basophils, abs.	ALT, 37°C
Eosinophils, %	Alkaline Phosphatase, 37°C
Eosinophils, abs.	AST, 37°C
Erythrocytes	Bilirubin, total
Haematocrit	Cholinesterase
Haemoglobin	Cholesterol
HbA1c	Creatinine
Leucocytes	hs-CRP
Lymphocytes, %	Glucose, serum
Lymphocytes, abs.	Gamma-GT, 37°C
MCH	HDL-Cholesterol
MCHC	LDL-Cholesterol
MCV	Potassium
Monocytes, %	Sodium
Monocytes, abs.	Total Protein
Neutrophils, %	Triglycerides
Neutrophils, abs.	Uric Acid
Platelets	Urea/BUN
RDW	**Proteins – Serum**
Reticulocytes	Rheumatoid factor H 35.9
Reticulocytes	**Hormones – Serum**
Reticulocytes, abs.	Insulin
	TSH (basal)

Stool, urine and saliva samples

The samples listed in Table 2 are collected at each visit, except for stool samples on T2 (fasting week) and immediately frozen and stored at -80°C. Stool characteristics are recorded at the time of the sampling. Faecal samples represent the main sample type for resolving the dynamic processes driven by microbiome in the gut. Also, as the gut microbiome is prone to diurnal fluctuations, the stool samples are collected in the morning, as far as possible.

For peer review only

Methods applied to samples

Biomolecular extractions

The collected stool samples undergo a biomolecular extraction procedure to allow isolation of concomitant DNA, RNA, proteins, peptides and metabolites from single, unique faecal water samples; this process involves cryo-milling the samples in liquid nitrogen, disassociating metabolites from membrane and cell wall components in a solvent mixture of methanol, chloroform and water and lastly proteins and RNA extraction by a methanol/chloroform and phenol buffer [66, 67]. Faecal water is recovered following centrifugation and filtration, at low-speed or low-flow, respectively, to avoid cell lysis. Nucleic acids are preserved by the addition of ribonuclease inhibitors and isolated by silica-column-based techniques. This protocol involves the use of a robotic platform, ensuring a higher level of standardisation and reproducibility[2].

Coupled metagenomic and metatranscriptomic analyses

Prior to sequencing library preparation, internal standards are introduced to obtain quantitative sequencing data[68]. Contamination-free metagenomic (MG) and metatranscriptomic (MT) data is generated, processed and analysed using the Integrated Meta-omics Pipeline (IMP)[45], which incorporates pre-processing, assembly, gene annotation, mapping of reads, single nucleotide polymorphism calling, data normalisation as well as analyses of community structure and function in a fully reproducible software framework based on Docker. The MG and MT data is specifically screened for enrichments in genes and pathways with known immunogenic properties[69]. The extracellular biomolecules are linked to specific microbial populations based on the intracellular metagenomic data [70]. In addition, the sequencing data is mapped against genomes of food components[44]. The quantitative data is also related to microbial population sizes to determine the contribution of the resolved microbial populations in stool to the extracellular DNA and RNA complements[71].

Metaproteomics

For the metaproteomic analyses, filtration is used to separate extracellular peptides from the obtained (poly)peptides. The resulting smaller fractions are then desalted and analysed without proteolytic digestion via liquid chromatography (LC) and mass spectrometry (MS) on an EasyNano-LC coupled online to a QExactive-Plus mass spectrometer (ThermoScientific, Waltham, USA). The identification of ribosomal peptides is done with an integrated catalogue of MG and MT data, while the non-ribosomal peptides are identified using different tools, i.e., MyriMatch, DirecTag as well as CycloBranch[45, 72, 73]. The metaproteomic data also allows identification of extracellular (poly)peptides with possible pathogenic functions including protein misfolding and molecular mimicry[74, 75].

Metabolomics

Metabolomic data is analysed using a combination of targeted and untargeted approaches [44, 67, 76]. This highlights the major metabolite classes produced by the gut microbiome with an effect on human physiology including organic acids, SCFA, lipids, branched-chain fatty acids, branched-chain amino acids, vitamins, bile acids and neurotransmitters. Besides external compound calibration series for quantification and quality control samples to ensure data normalisation and data acquisition quality assessment, the metabolite extraction fluid is fortified with multiple internal standards to improve method precision and accuracy[77, 78]. The data is compared to in-house databases and public mass spectral libraries to identify known metabolites. The metabolomic data complements the

For peer review only

metagenomic and metatranscriptomic data and thus allows further establishments of conclusive links to metabolic properties in the gut.

Deep immune profiling

Deep immune profiling is done using a recently established and optimised panel of metal-labelled antibodies together with cytometry coupled to mass spectrometry (MS), the Maxpar Direct Immune Profiling System (MDIPA). This approach allows the simultaneous quantification of 38 parameters on single cells. Whole blood is stained with the MDIPA kit and stabilised with Proteomic stabiliser Prot-1 (501351694, Smart Tube Inc., Las Vegas) before storage at -80°C. The quantified immune cells included in the MDIPA panel are CD3+, CD4+, CD8+, monocytes, dendritic cells, granulocytes, MAIT, T cells, NK and B cells[79]. Cytokine expression profiles is analysed on blood plasma using the Human Luminex performance Cytokine Panel (R&D Systems Europe, Abingdon), measuring CCL3, CCL4, CCL5, GM-CSF, IL-1β, IL-2, IL-4, IL-5, IL-6, IL-8, IL-10, IL-12p70, IL-13, IL-15, IL-18, IL-21, IL-27, IL-33, IFN-β, Galectin-1, IFN-γ and TNF-α [69].

Gut-on-a-chip models

PBMCs isolated from T0 blood samples are co-cultured with gut-derived microbes under physiologically representative conditions using the gut-on-a-chip model HuMiX[80]. This model of the human gastrointestinal interface allows the investigation of the interactions between immune, epithelial and bacterial cells and specifically the response to fasting in personalised in vitro models.

The Expobiome Map

The Expobiome Map (https://expobiome.lcsb.uni.lu) illustrates the diverse complex of microbial immunogenic molecules, including nucleic acids, (poly)peptides, structural molecules, and metabolites. The interactions between this "expobiome" and human immune pathways are encoded in the context of chronic diseases[1]. The ExpoBiome Map is visualised using the MINERVA Platform[81]. Clicking on different elements on the map reveals factors they affect and are affected by, allowing an easier navigation through the complex relationships between individual microbiome components in relation to human disease. The multi-omics data generated in the present study will be integrated with the Map.

Exploratory analysis of novel host-microbiome interactions

Unknown non-ribosomal peptides or metabolite features are associated through correlation with transcripts, proteins, and metabolites. Extracellular DNA fragments, as well as transcripts, proteins and ribosomal peptides are linked to their genomic context by using IMP[45]. The data generated by the project will be connected and collated to existing, publicly available datasets.

Outcome parameters

Primary Outcome

The primary endpoint of the study is the characterisation of the gut microbiome. The evaluation includes both between-group and within-group differences in the longitudinal study arms with the fasting intervention.

Secondary Outcome Measures

Secondary outcomes include the identification of common and disease-specific molecular signatures and the characterisation of microbiome-derived effector molecules impacting the innate and adaptive

For peer review only

immune pathways. Furthermore, several additional parameters mentioned in *Anthropometric data and questionnaires* are assessed over a period of 12 months.

Sample size and power calculation

A power calculation using pilot metatranscriptomic data based on faecal extracellular RNA samples was performed to determine the number of subjects to be recruited for the ExpoBiome project. The obtained relative abundances of genera were used for the calculation of the required sample size per group. The power calculation was based on the algorithm as described by Tusher, Tibshirani, and Chu[82]. To achieve a power of 90% (at $\alpha = 0.05$), a total of 50 individuals per group (RA, PD, healthy controls) must be analysed. Considering any possible dropouts, 20% additional subjects are recruited, resulting in a total number of 180 individuals, i.e., 60 per group. For the longitudinal part, a subset of 60 adult individuals (30 patients with Parkinson's disease and 30 patients with rheumatoid arthritis) are selected, based on their ability and willingness to participate in the longitudinal part of the study (12 months follow-up). The selected number of participants for the longitudinal study is based on feasibility due to the complexity and high costs of the clinical trial. The total number of subjects in the longitudinal study can be smaller, as each individual serves as their own control.

Adverse events

There are no major risks expected for participants. Minor common adverse effects of PF might include headaches, nausea, insomnia, back pain, dyspepsia and fatigue[83]. Any occurring adverse events are recorded at each visit in REDCap[65]. Serious adverse events are communicated to the study coordinator and principal investigator within 24 h of their report.

Data management, monitoring, analysis, and evaluation of data

The study participants receive a study ID (pseudonym) which is used for all collected data. Self-administered questionnaires are directly recorded in REDCap. Participant files are kept for at least 10 years at the respective clinical sites.

Weekly meetings between the study team, the different clinical partners, and the principal investigator, ensure a close monitoring of the data. Any occurring adverse events or other issues are thus handled immediately.

Different statistical tests are performed according to the nature of the data. A premature termination of the study is not envisaged; therefore, no interim analysis is done. Different correlation measures are applied, including Spearman correlation, mutual information on discretised data, distance correlation, maximum information criterion, local similarity analysis and the bioenv approach. Comparison across all omic levels allows identification of common and disease-specific signatures. Multivariate machine learning is used to link different data features to observed patterns. For additional confounding factors, especially in the cross-sectional study, multivariate statistical analysis will be performed. These factors will be accounted for by including confounders in the analysis, e.g., as covariate in the statistical models.

The longitudinal part of the study continues for a period of 12 months. After finalisation of this period, there is no follow-up of the participants. Interesting findings will be further validated using the existing sample set and analyses may be performed on additionally collected samples.

The SPIRIT - checklist (Standard Protocol Items: Recommendations for Interventional Trials) was used to write this protocol [84].

For peer review only - http://bmjopen.bmj.com/site/about/guidelines.xhtml

Trial status

The recruitment for the ExpoBiome study started in April 2021 and is currently ongoing. All study participants should be recruited by the end of 2022. The sample collection will take place from April 2021 to November 2023.

Ethics and dissemination

Ethical approval was obtained to plan and conduct the trial from the institutional review board of the Charité-Universitätsmedizin Berlin (EA1/204/19), the ethics committee of the state medical association (Landesärztekammer) of Hessen (2021-2230-zvBO) and the Ethics Review Panel (ERP) of the University of Luxembourg (ERP 21-001-A ExpoBiome). The results of this study will be disseminated through peer-reviewed publications, scientific presentations, as well as press releases and social media postings (Twitter, LinkedIn). Study participants will be contacted and informed by the respective clinical sites about the outcome and results of the study, once the data analysis has been completed (dissemination phase).

Acknowledgements

We thank Dr. Catharina Delebinski, Melanie Dell'Oro, Grit Langhans, Ursula Reuß, Maik Schröder and Nadine Sylvester for their support during the study.

Author contributions:

Study design and protocol were done by Bérénice Hansen, Cédric C. Laczny, Jochen G. Schneider, Paul Wilmes; the interventional concept was drawn by Etienne Hanslian, Daniela Liebscher, Andreas Michalsen, Anika Hartmann, Brit Mollenhauer, Sebastian Schade, Nico Steckhan, Jochen G. Schneider, Paul Wilmes; the clinical trial was designed and is conducted by Etienne Hanslian, Daniela Liebscher, Andreas Michalsen, Anika Hartmann, Brit Mollenhauer, Sebastian Schade; the procured funding was provided by Paul Wilmes; the planning of high-throughput applications, statistical planning, sample size calculation and randomisation were defined by Cédric C. Laczny, Jochen G. Schneider, Paul Wilmes, Kirsten Roomp; the initial draft of the manuscript and coordination of the editing process were performed by Bérénice Hansen; the protocol preparation has been done by Bérénice Hansen, Audrey Frachet-Bour, Janine Habier; the planning of the data analysis was done by Cédric C. Laczny, Jochen G. Schneider, Paul Wilmes, Kirsten Roomp, Velma T.E. Aho, Marek Ostaszewski; all authors contributed equally with edits, comments and feedback, read and approved the final manuscript.

Funding statement: This project has received funding from the European Research Council (ERC) under the European Union's Horizon 2020 research and innovation program (grant agreement No. 863664). This work was supported by the Luxembourg National Research Fund (FNR) under grant PRIDE/11823097.

Competing interests statement

None declared.

Supplements

The SPIRIT checklist was used to write our report[84].

For peer review only

References

1. Wilmes, P., et al., *The gut microbiome molecular complex in human health and disease.* Cell Host Microbe, 2022. **30**(9): p. 1201-1206.
2. De Saedeleer, B., et al., *Systematic characterization of human gut microbiome-secreted molecules by integrated multi-omics.* ISME Communications, 2021. **1**(1): p. 82.
3. Greenhalgh, K., et al., *The human gut microbiome in health: establishment and resilience of microbiota over a lifetime.* Environ Microbiol, 2016. **18**(7): p. 2103-16.
4. Hall, A.B., A.C. Tolonen, and R.J. Xavier, *Human genetic variation and the gut microbiome in disease.* Nature Reviews Genetics, 2017. **18**(11): p. 690-699.
5. Baldini, F., et al., *Parkinson's disease-associated alterations of the gut microbiome predict disease-relevant changes in metabolic functions.* BMC Biol, 2020. **18**(1): p. 62.
6. Yoo, J.Y., et al., *Gut Microbiota and Immune System Interactions.* Microorganisms, 2020. **8**(10).
7. Sonnenburg, J.L. and F. Bäckhed, *Diet–microbiota interactions as moderators of human metabolism.* Nature, 2016. **535**(7610): p. 56-64.
8. Zmora, N., J. Suez, and E. Elinav, *You are what you eat: diet, health and the gut microbiota.* Nat Rev Gastroenterol Hepatol, 2019. **16**(1): p. 35-56.
9. Guo, Q., et al., *Rheumatoid arthritis: pathological mechanisms and modern pharmacologic therapies.* Bone Res, 2018. **6**: p. 15.
10. Healthline, V.L. *Rheumatoid Arthritis by the Numbers: Facts, Statistics, and You*. 2021.
11. Scherer, H.U., T. Häupl, and G.R. Burmester, *The etiology of rheumatoid arthritis.* Journal of Autoimmunity, 2020. **110**: p. 102400.
12. Bodkhe, R., B. Balakrishnan, and V. Taneja, *The role of microbiome in rheumatoid arthritis treatment.* Ther Adv Musculoskelet Dis, 2019. **11**: p. 1759720x19844632.
13. Chen, J., et al., *An expansion of rare lineage intestinal microbes characterizes rheumatoid arthritis.* Genome Medicine, 2016. **8**(1): p. 43.
14. Kitamura, K., et al., *Oral and Intestinal Bacterial Substances Associated with Disease Activities in Patients with Rheumatoid Arthritis: A Cross-Sectional Clinical Study.* J Immunol Res, 2022. **2022**: p. 6839356.
15. Wu, H.J., et al., *Gut-residing segmented filamentous bacteria drive autoimmune arthritis via T helper 17 cells.* Immunity, 2010. **32**(6): p. 815-27.
16. Scher, J.U., et al., *Expansion of intestinal Prevotella copri correlates with enhanced susceptibility to arthritis.* eLife, 2013. **2**: p. e01202.
17. Zhang, X., et al., *The oral and gut microbiomes are perturbed in rheumatoid arthritis and partly normalized after treatment.* Nature Medicine, 2015. **21**(8): p. 895-905.
18. Lubomski, M., et al., *Parkinson's disease and the gastrointestinal microbiome.* J Neurol, 2020. **267**(9): p. 2507-2523.
19. Opara, J., et al., *Motor assessment in Parkinson`s disease.* Ann Agric Environ Med, 2017. **24**(3): p. 411-415.
20. Tysnes, O.B. and A. Storstein, *Epidemiology of Parkinson's disease.* J Neural Transm (Vienna), 2017. **124**(8): p. 901-905.

For peer review only

21. Garcia, P., et al., *Neurodegeneration and neuroinflammation are linked, but independent of alpha-synuclein inclusions, in a seeding/spreading mouse model of Parkinson's disease.* Glia, 2022. **70**(5): p. 935-960.
22. Heintz-Buschart, A. and P. Wilmes, *Human Gut Microbiome: Function Matters.* Trends Microbiol, 2018. **26**(7): p. 563-574.
23. Romano, S., et al., *Meta-analysis of the Parkinson’s disease gut microbiome suggests alterations linked to intestinal inflammation.* npj Parkinson's Disease, 2021. **7**(1): p. 27.
24. Becker, A., et al., *Effects of Resistant Starch on Symptoms, Fecal Markers, and Gut Microbiota in Parkinson's Disease - The RESISTA-PD Trial.* Genomics Proteomics Bioinformatics, 2022. **20**(2): p. 274-287.
25. Hall, D.A., et al., *An open label, non-randomized study assessing a prebiotic fiber intervention in a small cohort of Parkinson's disease participants.* Nat Commun, 2023. **14**(1): p. 926.
26. Mertsalmi, T.H., et al., *More than constipation - bowel symptoms in Parkinson's disease and their connection to gut microbiota.* European journal of neurology, 2017. **24**(11): p. 1375-1383.
27. Dogra, N., R.J. Mani, and D.P. Katare, *The Gut-Brain Axis: Two Ways Signaling in Parkinson's Disease.* Cell Mol Neurobiol, 2022. **42**(2): p. 315-332.
28. Forsyth, C.B., et al., *Increased intestinal permeability correlates with sigmoid mucosa alpha-synuclein staining and endotoxin exposure markers in early Parkinson's disease.* PLoS One, 2011. **6**(12): p. e28032.
29. Devos, D., et al., *Colonic inflammation in Parkinson's disease.* Neurobiol Dis, 2013. **50**: p. 42-8.
30. Britannica, *The Editors of Encyclopaedia. "fasting".* Encyclopedia Britannica,, Accessed 3 October 2022. https://www.britannica.com/topic/fasting.
31. Hartmann, A.M., et al., *Efficacy of therapeutic fasting and plant-based diet in patients with rheumatoid arthritis (NutriFast): study protocol for a randomised controlled clinical trial.* BMJ Open, 2021. **11**(8): p. e047758.
32. Michalsen, A., *Prolonged fasting as a method of mood enhancement in chronic pain syndromes: a review of clinical evidence and mechanisms.* Curr Pain Headache Rep, 2010. **14**(2): p. 80-7.
33. Vargas-Molina, S., et al., *Effects of a low-carbohydrate ketogenic diet on health parameters in resistance-trained women.* Eur J Appl Physiol, 2021. **121**(8): p. 2349-2359.
34. Mattison, J.A., et al., *Caloric restriction improves health and survival of rhesus monkeys.* Nature Communications, 2017. **8**(1): p. 14063.
35. Maifeld, A., et al., *Fasting alters the gut microbiome reducing blood pressure and body weight in metabolic syndrome patients.* Nature Communications, 2021. **12**(1): p. 1970.
36. Choi, I.Y., et al., *A Diet Mimicking Fasting Promotes Regeneration and Reduces Autoimmunity and Multiple Sclerosis Symptoms.* Cell Rep, 2016. **15**(10): p. 2136-2146.
37. Jordan, S., et al., *Dietary Intake Regulates the Circulating Inflammatory Monocyte Pool.* Cell, 2019. **178**(5): p. 1102-1114.e17.
38. Neth, B.J., et al., *The Role of Intermittent Fasting in Parkinson's Disease.* Front Neurol, 2021. **12**: p. 682184.

For peer review only

39. Cignarella, F., et al., *Intermittent Fasting Confers Protection in CNS Autoimmunity by Altering the Gut Microbiota.* Cell Metab, 2018. **27**(6): p. 1222-1235.e6.
40. Mesnage, R., et al., *Changes in human gut microbiota composition are linked to the energy metabolic switch during 10 d of Buchinger fasting.* J Nutr Sci, 2019. **8**: p. e36.
41. Magne, F., et al., *The Firmicutes/Bacteroidetes Ratio: A Relevant Marker of Gut Dysbiosis in Obese Patients?* Nutrients, 2020. **12**(5).
42. Purchiaroni, F., et al., *The role of intestinal microbiota and the immune system.* Eur Rev Med Pharmacol Sci, 2013. **17**(3): p. 323-33.
43. Leeming, E.R., et al., *Effect of Diet on the Gut Microbiota: Rethinking Intervention Duration.* Nutrients, 2019. **11**(12): p. 2862.
44. Heintz-Buschart, A., et al., *Integrated multi-omics of the human gut microbiome in a case study of familial type 1 diabetes.* Nature Microbiology, 2016. **2**(1): p. 16180.
45. Narayanasamy, S., et al., *IMP: a pipeline for reproducible reference-independent integrated metagenomic and metatranscriptomic analyses.* Genome Biology, 2016. **17**(1): p. 260.
46. Wilmes, P., A. Heintz-Buschart, and P.L. Bond, *A decade of metaproteomics: where we stand and what the future holds.* Proteomics, 2015. **15**(20): p. 3409-17.
47. Gabel, K., et al., *Effects of 8-hour time restricted feeding on body weight and metabolic disease risk factors in obese adults: A pilot study.* Nutr Healthy Aging, 2018. **4**(4): p. 345-353.
48. Hughes, A.J., et al., *A clinicopathologic study of 100 cases of Parkinson's disease.* Arch Neurol, 1993. **50**(2): p. 140-8.
49. Hoehn, M.M. and M.D. Yahr, *Parkinsonism.* onset, progression, and mortality, 1967. **17**(5): p. 427-427.
50. Kay, J. and K.S. Upchurch, *ACR/EULAR 2010 rheumatoid arthritis classification criteria.* Rheumatology (Oxford), 2012. **51 Suppl 6**: p. vi5-9.
51. Feng, X., et al., *Body Mass Index and the Risk of Rheumatoid Arthritis: An Updated Dose-Response Meta-Analysis.* Biomed Res Int, 2019. **2019**: p. 3579081.
52. Wells G, B.J., Teng J, et al, *Validation of the 28-joint Disease Activity Score (DAS28) and European League Against Rheumatism response criteria based on C-reactive protein against disease progression in patients with rheumatoid arthritis, and comparison with the DAS28 based on erythrocyte sedimentation rate.* Annals of the Rheumatic Diseases 2009. **68**: p. 954-960.
53. Trenkwalder, C., et al., *Parkinson's disease sleep scale--validation of the revised version PDSS-2.* Mov Disord, 2011. **26**(4): p. 644-52.
54. Bushnell, D.M. and M.L. Martin, *Quality of life and Parkinson's disease: translation and validation of the US Parkinson's Disease Questionnaire (PDQ-39).* Qual Life Res, 1999. **8**(4): p. 345-50.
55. Smolen, J.S., et al., *A simplified disease activity index for rheumatoid arthritis for use in clinical practice.* Rheumatology (Oxford), 2003. **42**(2): p. 244-57.
56. Raspe, H.H., Hagedorn, U., Kohlmann, T., & Mattussek, S., *Der Funktionsfragebogen Hannover (FFbH): Ein Instrument zur Funktionsdiagnostik bei polyartikulären Gelenkerkrankungen*, in *Ergebnisse sozialwissenschaftlicher Evaluation eines Modellversuchs (pp. 164-182)*. 1990, Schattauer Verlag.
57. Goetz, C.G., et al., *Movement Disorder Society-sponsored revision of the Unified Parkinson's Disease Rating Scale (MDS-UPDRS): Process, format, and clinimetric testing plan.* Mov Disord, 2007. **22**(1): p. 41-7.

For peer review only

58. Chaudhuri, K.R., et al., *International multicenter pilot study of the first comprehensive self-completed nonmotor symptoms questionnaire for Parkinson's disease: the NMSQuest study.* Mov Disord, 2006. **21**(7): p. 916-23.

59. Chaudhuri, K.R., et al., *The metric properties of a novel non-motor symptoms scale for Parkinson's disease: Results from an international pilot study.* Mov Disord, 2007. **22**(13): p. 1901-11.

60. Wolfe, F., *A brief clinical health assessment instrument clinhaq.* Arthritis and Rheumatism, 1989. **32**(4 Suppl): p. S99-S99.

61. Lewis, S.J. and K.W. Heaton, *Stool form scale as a useful guide to intestinal transit time.* Scand J Gastroenterol, 1997. **32**(9): p. 920-4.

62. Topp, C.W., et al., *The WHO-5 Well-Being Index: a systematic review of the literature.* Psychother Psychosom, 2015. **84**(3): p. 167-76.

63. Zigmond, A.S. and R.P. Snaith, *The hospital anxiety and depression scale.* Acta Psychiatr Scand, 1983. **67**(6): p. 361-70.

64. McNair DM, L.M., Droppleman LF, *Edits Manual for the Profile of Mood States (Poms). Rev ed.* . San Diego: Educational and Industrial Testing Service, 1992.

65. Harris, P.A., et al., *Research electronic data capture (REDCap)--a metadata-driven methodology and workflow process for providing translational research informatics support.* J Biomed Inform, 2009. **42**(2): p. 377-81.

66. Wilmes, P., Roume, H., Hiller, K. & Cordes, T., *Method and kit for the isolation of genomic DNA, RNA, proteins and metabolites from a single biological sample.*, in *World Intellectual Property Organization*, C.D.R.P.-G.L. Université Du Luxembourg, Editor. 2014: Switzerland.

67. Roume, H., et al., *A biomolecular isolation framework for eco-systems biology.* The ISME Journal, 2013. **7**(1): p. 110-121.

68. Locati, M.D., et al., *Improving small RNA-seq by using a synthetic spike-in set for size-range quality control together with a set for data normalization.* Nucleic Acids Res, 2015. **43**(14): p. e89.

69. Wampach, L., et al., *Birth mode is associated with earliest strain-conferred gut microbiome functions and immunostimulatory potential.* Nature Communications, 2018. **9**(1): p. 5091.

70. Albanese, D. and C. Donati, *Strain profiling and epidemiology of bacterial species from metagenomic sequencing.* Nature Communications, 2017. **8**(1): p. 2260.

71. Vandeputte, D., et al., *Quantitative microbiome profiling links gut community variation to microbial load.* Nature, 2017. **551**(7681): p. 507-511.

72. Tang, H., S. Li, and Y. Ye, *A Graph-Centric Approach for Metagenome-Guided Peptide and Protein Identification in Metaproteomics.* PLoS Comput Biol, 2016. **12**(12): p. e1005224.

73. Tabb, D.L., C.G. Fernando, and M.C. Chambers, *MyriMatch: highly accurate tandem mass spectral peptide identification by multivariate hypergeometric analysis.* J Proteome Res, 2007. **6**(2): p. 654-61.

74. Heintz-Buschart, A., et al., *The nasal and gut microbiome in Parkinson's disease and idiopathic rapid eye movement sleep behavior disorder.* Mov Disord, 2018. **33**(1): p. 88-98.

75. Chen, S.G., et al., *Exposure to the Functional Bacterial Amyloid Protein Curli Enhances Alpha-Synuclein Aggregation in Aged Fischer 344 Rats and Caenorhabditis elegans.* Scientific Reports, 2016. **6**(1): p. 34477.

For peer review only

76. Wilmes, P., et al., *Metabolome-proteome differentiation coupled to microbial divergence.* mBio, 2010. **1**(5).
77. Kim, D.H., et al., *LC-MS-based absolute metabolite quantification: application to metabolic flux measurement in trypanosomes.* Metabolomics, 2015. **11**(6): p. 1721-1732.
78. Lei, Z., D.V. Huhman, and L.W. Sumner, *Mass spectrometry strategies in metabolomics.* J Biol Chem, 2011. **286**(29): p. 25435-42.
79. *Immunophenotyping assessment in a COVID-19 cohort (IMPACC): A prospective longitudinal study.* Sci Immunol, 2021. **6**(62).
80. Shah, P., et al., *A microfluidics-based in vitro model of the gastrointestinal human–microbe interface.* Nature Communications, 2016. **7**(1): p. 11535.
81. Aho, V.T.E., et al., *SnapShot: The Expobiome Map.* Cell Host Microbe, 2022. **30**(9): p. 1340-1340.e1.
82. Tusher, V.G., R. Tibshirani, and G. Chu, *Significance analysis of microarrays applied to the ionizing radiation response.* Proc Natl Acad Sci U S A, 2001. **98**(9): p. 5116-21.
83. Finnell, J.S., et al., *Is fasting safe? A chart review of adverse events during medically supervised, water-only fasting.* BMC Complementary and Alternative Medicine, 2018. **18**(1): p. 67.
84. Chan, A.-W., et al., *SPIRIT 2013 explanation and elaboration: guidance for protocols of clinical trials.* BMJ : British Medical Journal, 2013. **346**: p. e7586.

Figure Legends

Figure 1. Study design. This figure illustrates the study design with five different arms in total, two of which continue with the longitudinal part of the study. Visits take place at the clinical sites at each timepoint and include the collection of the displayed samples. This image was generated using Biorender software (http://www.biorender.com). T, timepoint; W, week; D, day; M, month.

For peer review only

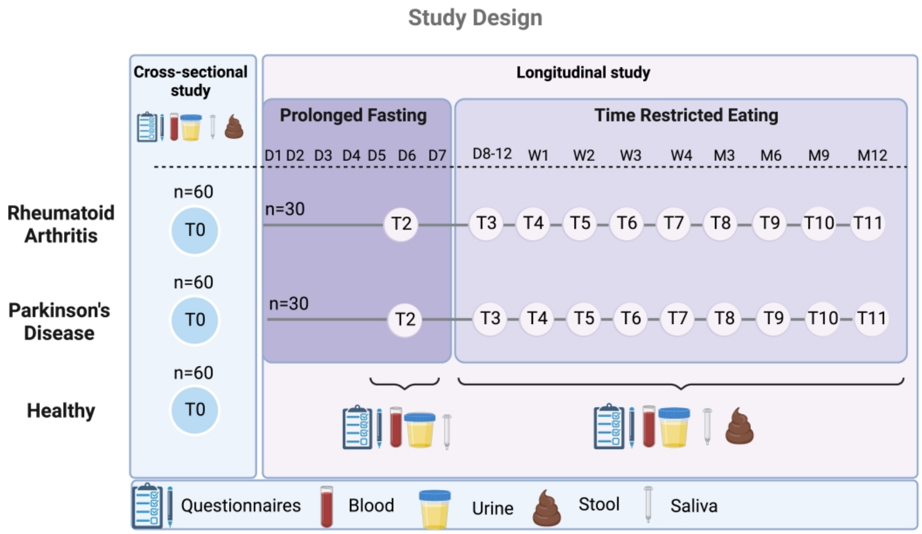

Figure 1. Study design. This figure illustrates the study design with five different arms in total, two of which continue with the longitudinal part of the study. Visits take place at the clinical sites at each timepoint and include the collection of the displayed samples. This image was generated using Biorender software (http://www.biorender.com). T, timepoint; W, week; D, day; M, month.

279x177mm (600 x 600 DPI)

Reporting checklist for protocol of a clinical trial.

Based on the SPIRIT guidelines.

Instructions to authors

Complete this checklist by entering the page numbers from your manuscript where readers will find each of the items listed below.

Your article may not currently address all the items on the checklist. Please modify your text to include the missing information. If you are certain that an item does not apply, please write "n/a" and provide a short explanation.

Upload your completed checklist as an extra file when you submit to a journal.

In your methods section, say that you used the SPIRITreporting guidelines, and cite them as:

Chan A-W, Tetzlaff JM, Gøtzsche PC, Altman DG, Mann H, Berlin J, Dickersin K, Hróbjartsson A, Schulz KF, Parulekar WR, Krleža-Jerić K, Laupacis A, Moher D. SPIRIT 2013 Explanation and Elaboration: Guidance for protocols of clinical trials. BMJ. 2013;346:e7586

		Reporting Item	Page Number
**Administrative information**			
Title	#1	Descriptive title identifying the study design, population, interventions, and, if applicable, trial acronym	1
Trial registration	#2a	Trial identifier and registry name. If not yet registered, name of intended registry	2
Trial registration: data set	#2b	All items from the World Health Organization Trial Registration Data Set	n/a
Protocol version	#3	Date and version identifier	n/a
Funding	#4	Sources and types of financial, material, and other support	13
Roles and responsibilities: contributorship	#5a	Names, affiliations, and roles of protocol contributors	1

For peer review only

Roles and responsibilities: sponsor contact information	#5b	Name and contact information for the trial sponsor	1
Roles and responsibilities: sponsor and funder	#5c	Role of study sponsor and funders, if any, in study design; collection, management, analysis, and interpretation of data; writing of the report; and the decision to submit the report for publication, including whether they will have ultimate authority over any of these activities	1, 13
Roles and responsibilities: committees	#5d	Composition, roles, and responsibilities of the coordinating centre, steering committee, endpoint adjudication committee, data management team, and other individuals or groups overseeing the trial, if applicable (see Item 21a for data monitoring committee)	1, 13
**Introduction**			
Background and rationale	#6a	Description of research question and justification for undertaking the trial, including summary of relevant studies (published and unpublished) examining benefits and harms for each intervention	5
Background and rationale: choice of comparators	#6b	Explanation for choice of comparators	5
Objectives	#7	Specific objectives or hypotheses	5
Trial design	#8	Description of trial design including type of trial (eg, parallel group, crossover, factorial, single group), allocation ratio, and framework (eg, superiority, equivalence, non-inferiority, exploratory)	5
**Methods: Participants, interventions, and outcomes**			
Study setting	#9	Description of study settings (eg, community clinic, academic hospital) and list of countries where data will be collected. Reference to where list of study sites can be obtained	6

For peer review only

Eligibility criteria	#10	Inclusion and exclusion criteria for participants. If applicable, eligibility criteria for study centres and individuals who will perform the interventions (eg, surgeons, psychotherapists)	6
Interventions: description	#11a	Interventions for each group with sufficient detail to allow replication, including how and when they will be administered	5,7
Interventions: modifications	#11b	Criteria for discontinuing or modifying allocated interventions for a given trial participant (eg, drug dose change in response to harms, participant request, or improving / worsening disease)	5,7
Interventions: adherance	#11c	Strategies to improve adherence to intervention protocols, and any procedures for monitoring adherence (eg, drug tablet return; laboratory tests)	7
Interventions: concomitant care	#11d	Relevant concomitant care and interventions that are permitted or prohibited during the trial	6,7
Outcomes	#12	Primary, secondary, and other outcomes, including the specific measurement variable (eg, systolic blood pressure), analysis metric (eg, change from baseline, final value, time to event), method of aggregation (eg, median, proportion), and time point for each outcome. Explanation of the clinical relevance of chosen efficacy and harm outcomes is strongly recommended	10f
Participant timeline	#13	Time schedule of enrolment, interventions (including any run-ins and washouts), assessments, and visits for participants. A schematic diagram is highly recommended (see Figure)	5
Sample size	#14	Estimated number of participants needed to achieve study objectives and how it was determined, including clinical and statistical assumptions supporting any sample size calculations	12
Recruitment	#15	Strategies for achieving adequate participant enrolment to reach target sample size	5
**Methods: Assignment of interventions (for controlled trials)**			
Allocation: sequence generation	#16a	Method of generating the allocation sequence (eg, computer-generated random numbers), and list of any factors for stratification. To reduce predictability of a random sequence, details of any planned restriction (eg, blocking) should be	n/a

For peer review only

		provided in a separate document that is unavailable to those who enrol participants or assign interventions	
Allocation concealment mechanism	#16b	Mechanism of implementing the allocation sequence (eg, central telephone; sequentially numbered, opaque, sealed envelopes), describing any steps to conceal the sequence until interventions are assigned	n/a
Allocation: implementation	#16c	Who will generate the allocation sequence, who will enrol participants, and who will assign participants to interventions	n/a
Blinding (masking)	#17a	Who will be blinded after assignment to interventions (eg, trial participants, care providers, outcome assessors, data analysts), and how	n/a
Blinding (masking): emergency unblinding	#17b	If blinded, circumstances under which unblinding is permissible, and procedure for revealing a participant's allocated intervention during the trial	n/a
**Methods: Data collection, management, and analysis**			
Data collection plan	#18a	Plans for assessment and collection of outcome, baseline, and other trial data, including any related processes to promote data quality (eg, duplicate measurements, training of assessors) and a description of study instruments (eg, questionnaires, laboratory tests) along with their reliability and validity, if known. Reference to where data collection forms can be found, if not in the protocol	10f
Data collection plan: retention	#18b	Plans to promote participant retention and complete follow-up, including list of any outcome data to be collected for participants who discontinue or deviate from intervention protocols	7,12
Data management	#19	Plans for data entry, coding, security, and storage, including any related processes to promote data quality (eg, double data entry; range checks for data values). Reference to where details of data management procedures can be found, if not in the protocol	12
Statistics: outcomes	#20a	Statistical methods for analysing primary and secondary outcomes. Reference to where other details of the statistical analysis plan can be found, if not in the protocol	12f

For peer review only

Statistics: additional analyses	#20b	Methods for any additional analyses (eg, subgroup and adjusted analyses)	12f
Statistics: analysis population and missing data	#20c	Definition of analysis population relating to protocol non-adherence (eg, as randomised analysis), and any statistical methods to handle missing data (eg, multiple imputation)	12f
**Methods: Monitoring**			
Data monitoring: formal committee	#21a	Composition of data monitoring committee (DMC); summary of its role and reporting structure; statement of whether it is independent from the sponsor and competing interests; and reference to where further details about its charter can be found, if not in the protocol. Alternatively, an explanation of why a DMC is not needed	12f
Data monitoring: interim analysis	#21b	Description of any interim analyses and stopping guidelines, including who will have access to these interim results and make the final decision to terminate the trial	n/a
Harms	#22	Plans for collecting, assessing, reporting, and managing solicited and spontaneously reported adverse events and other unintended effects of trial interventions or trial conduct	12
Auditing	#23	Frequency and procedures for auditing trial conduct, if any, and whether the process will be independent from investigators and the sponsor	n/a
**Ethics and dissemination**			
Research ethics approval	#24	Plans for seeking research ethics committee / institutional review board (REC / IRB) approval	2
Protocol amendments	#25	Plans for communicating important protocol modifications (eg, changes to eligibility criteria, outcomes, analyses) to relevant parties (eg, investigators, REC / IRBs, trial participants, trial registries, journals, regulators)	2, 13
Consent or assent	#26a	Who will obtain informed consent or assent from potential trial participants or authorised surrogates, and how (see Item 32)	6

For peer review only

Consent or assent: ancillary studies	#26b	Additional consent provisions for collection and use of participant data and biological specimens in ancillary studies, if applicable	6
Confidentiality	#27	How personal information about potential and enrolled participants will be collected, shared, and maintained in order to protect confidentiality before, during, and after the trial	12
Declaration of interests	#28	Financial and other competing interests for principal investigators for the overall trial and each study site	13
Data access	#29	Statement of who will have access to the final trial dataset, and disclosure of contractual agreements that limit such access for investigators	12f
Ancillary and post trial care	#30	Provisions, if any, for ancillary and post-trial care, and for compensation to those who suffer harm from trial participation	n/a
Dissemination policy: trial results	#31a	Plans for investigators and sponsor to communicate trial results to participants, healthcare professionals, the public, and other relevant groups (eg, via publication, reporting in results databases, or other data sharing arrangements), including any publication restrictions	2, 13
Dissemination policy: authorship	#31b	Authorship eligibility guidelines and any intended use of professional writers	13
Dissemination policy: reproducible research	#31c	Plans, if any, for granting public access to the full protocol, participant-level dataset, and statistical code	n/a
**Appendices**			
Informed consent materials	#32	Model consent form and other related documentation given to participants and authorised surrogatess	6
Biological specimens	#33	Plans for collection, laboratory evaluation, and storage of biological specimens for genetic or molecular analysis in the current trial and for future use in ancillary studies, if applicable	7

The SPIRIT Explanation and Elaboration paper is distributed under the terms of the Creative Commons Attribution License CC-BY-NC. This checklist was completed on 07. November 2022 using https://www.goodreports.org/, a tool made by the EQUATOR Network in collaboration with Penelope.ai

For peer review only
